# Genome-first determination of the prevalence and penetrance of eight germline myeloid malignancy predisposition genes: a study of two population-based cohorts

**DOI:** 10.1038/s41375-024-02436-y

**Published:** 2024-11-06

**Authors:** Rachel M. Hendricks, Jung Kim, Jeremy S. Haley, Mark Louie Ramos, Uyenlinh L. Mirshahi, David J. Carey, Douglas R. Stewart, Lisa J. McReynolds

**Affiliations:** 1 Clinical Genetics Branch, Division of Cancer Epidemiology and Genetics, National Cancer Institute, Bethesda, MD USA; 2 Department of Genomic Health, Weis Center for Research, Geisinger Medical Center, Danville, PA USA; 3 Biostatistics Branch, Division of Cancer Epidemiology and Genetics, National Cancer Institute, Bethesda, MD USA

**Keywords:** Risk factors, Cancer genetics

## Abstract

It is estimated that 10% of individuals with a myeloid malignancy carry a germline susceptibility. Using the genome-first approach, in which individuals were ascertained on genotype alone, rather than clinical phenotype, we quantified the prevalence and penetrance of pathogenic germline variants in eight myeloid malignancy predisposition (gMMP) genes. *ANKRD26*, *CEBPA, DDX41, MECOM, SRP72, ETV6, RUNX1* and *GATA2*, were analyzed from the Geisinger MyCode DiscovEHR (*n* = 170,503) and the United Kingdom Biobank (UKBB, *n* = 469,595). We identified a high risk of myeloid malignancies (MM) (odds ratio[OR] all genes: DiscovEHR, 4.6 [95% confidential interval (CI) 2.1–9.7], *p* < 0.0001; UKBB, 6.0 [95% CI 4.3–8.2], *p* = 3.1 × 10^-27^), and decreased overall survival (hazard ratio [HR] DiscovEHR, 1.8 [95% CI 1.3–2.6], *p* = 0.00049; UKBB, 1.4 [95% CI 1.2–1.8], *p* = 8.4 × 10^-5^) amongst heterozygotes. Pathogenic *DDX41* variants were the most commonly identified, and in UKBB showed a significantly increased risk of MM (OR 5.7 [95% CI 3.9–8.3], *p* = 6.0 × 10^-20^) and increased all-cause mortality (HR 1.35 [95% CI 1.1–1.7], *p* = 0.0063). Through a genome-first approach, this study genetically ascertained individuals with a gMMP and determined their MM risk and survival.

## Introduction

Myelodysplastic syndrome and acute myeloid leukemia (MDS/AML) are most commonly sporadic (90%), however heritable predisposition to myeloid malignancies is increasingly recognized to be 6–10% in the literature [1]. Historically, myeloid malignancies and bone marrow failure were thought to be rare and isolated to the classical inherited bone marrow failure syndromes and the pediatric population. Recent genomic investigations have uncovered multiple genes with variable penetrance and expressivity that are associated with an increased risk of a hematological malignancy. These genes include hematopoietic transcription factors such as *RUNX1*, *CEBPA*, *GATA2*, and *ETV6*, but also genes important in other biological pathways, such as *ANKRD26* and *SRP72*. Several of these genes are also somatically mutated in sporadic MDS (i.e., *ETV6*, *DDX41*, *GATA2*) and AML (i.e., *RUNX1*, *CEPBA*, *GATA2*), while others appear enriched or exclusive to the germline inherited forms of myeloid malignancy predisposition (i.e., *ANKRD26*).

Advances in the understanding of myeloid malignancies were made possible by the introduction of next-generation sequencing technologies, both for germline as well as for sporadic disease, prompting re-evaluation of approaches to the diagnosis and management of individuals with germline risk of MDS/AML [2]. Recent International Consensus Classification and World Health Organization guidelines introduced new entities and refined criteria for germline myeloid malignancy diagnostic categories [3, 4]. These new guidelines divide the myeloid neoplasms with germline predisposition into three groups: 1) those without a platelet disorder or other organ system involvement, 2) those with a platelet disorder, and 3) those affecting multiple organ systems. The increasing awareness of heritable forms of myeloid disease has revealed that 6–33% of individuals with a hematologic malignancy may carry a germline susceptibility [1, 5–10]. However, the precise incidence and prevalence are not known since these estimates are from clinically ascertained cohorts from a targeted population, such as cases referred to specialty clinics. These cohorts may be potentially biased in their recruitment and ascertainment. Additionally, despite having detailed acquired somatic genetic information on large numbers of cases, a deep understanding of the role of germline genetics in MDS/AML is lacking [11].

The genome-first approach, in which individuals with disease-causing variants are ascertained based on genotype rather than a clinically identified phenotype [12], provides a unique opportunity to reduce ascertainment bias, accurately quantify prevalence and risk, and help inform findings from cancer-based cohorts. Large studies from a genome-first approach using population-scale data complements work done using a phenotype-first approach. To our knowledge, there are no published analyses that determine the prevalence and penetrance of deleterious variants in multiple myeloid malignancy predisposition genes using the genome-first approach. Therefore, we sought to quantify pathogenic/likely pathogenic (P/LP) germline variants from myeloid malignancy predisposition (gMMP) genes and determine the prevalence and penetrance of these cancer predispositions using exome-sequencing datasets from two large population-based cohorts.

Eight genes were included in the study- *ANKRD26*, *CEBPA*, *DDX41*, *ETV6*, *GATA2*, *MECOM*, *RUNX1*, and *SRP72*. *ANKRD26*, *GATA2*, *DDX41*, *CEBPA* and *RUNX1* are well-established myeloid malignancy predisposition genes, but the population prevalence is unknown since prior studies included cohorts ascertained on phenotype such as cancer registries or clinically identified cases series. Patients with germline *MECOM* pathogenic variants have been described as having a phenotype of bone marrow failure with radioulnar synostosis, although other case reports have noted patients without this skeletal abnormality as well. In addition to the thrombocytopenia observed, it has been suggested that these patients also have a predisposition to myeloid malignancies [1, 13–16]. *SRP72* has been identified in two families with bone marrow failure and myeloid dysplasia [17]. However, no other families have been identified with disease causing variants in this gene, and mouse studies do not show an associated phenotype [18]. These genes were included to determine if additional cases might be identified and give evidence to support the inclusion of them as myeloid malignancy predisposition genes. Early identification of gMMP is integral to providing optimal clinical care; thus in this study we used a genome-first approach to determine the prevalence and penetrance of these eight hereditary myeloid predispositions [2].

## Methods

### Myeloid malignancy predisposition genes

Germline variants from eight gMMP genes were assessed for this study: *ANKRD26* (OMIM: 610855), Thrombocytopenia 2 [19]; *CEBPA* (OMIM: 116897), Familial AML [20]; *DDX41* (OMIM: 608170), adult onset MDS [21]; *ETV6* (OMIM: 600618), Thrombocytopenia 5 [22]; *GATA2* (OMIM: 137295), GATA2 Deficiency [23]; *RUNX1* (OMIM: 151385), Familial Platelet disorder with propensity to AML [24]; *SRP72* (OMIM: 602122), Bone marrow failure syndrome 1 [17]; *MECOM* (OMIM: 165215), radioulnar synostosis with amegakaryocytic thrombocytopenia 2 [25].

### Study cohorts

#### Geisinger MyCode - DiscovEHR cohort

The MyCode Community Initiative is an unselected clinical population recruited from outpatient and inpatient sites within Geisinger Health System (GHS), a large regional medical system headquartered in Danville, PA, USA [26]. The DiscovEHR cohort includes 170,503 individuals who have consented to undergo exome sequencing and array genotyping as part of the collaboration between GHS and the Regeneron Genetics Center [27, 28]. The cohort characteristics have been described comprehensively elsewhere [28, 29]. All individuals are de-identified, and their genotyping information is linked to electronic health records (EHR) and the Geisinger Tumor Registry. Relevant clinical phenotypes were determined from GHS EHR or tumor registry data collected from January 1, 1996, to October 31, 2022. The MyCode age distribution and disease frequency is similar to the general adult population within GHS [26, 30]. This investigation was reviewed by the GHS Institutional Review Board and determined not to be human subject research.

#### United Kingdom Biobank (UKBB)

United Kingdom Biobank (UKBB) is a large-scale, population-based prospective study with exome sequencing data from 469,595 individuals [31]. Participants were recruited and assessed between 2006 and 2010 in assessment centers throughout the UK. The cohort characteristics have been described comprehensively elsewhere [32, 33]. Participants consented to provide self-completed questionnaires, interviews, physical and functional measures, linked inpatient EHR, cancer and death registry, as well as collection of blood and other samples. All genetic data for this study were generated using DNA samples from peripheral blood. The work described in this study was approved by UKBB under application number 54389.

#### Genome Aggregation Database (gnomAD)

The Genome Aggregation Database (gnomAD) consortium is a resource that contains both exome and genome sequencing data from a wide variety of large-scale sequencing projects, with the summary level data publicly available [34]. This study used the v3.1.2 data set (GRCh38) of 76,156 genomes (https://gnomad.broadinstitute.org/).

### Ethical compliance and study approval

This study was approved by all population (not including gnomAD) cohorts’ respective Institutional Review Boards, and informed consent was obtained for all patients. All clinical data from individuals in this study were de-identified. Samples from these different studies were shared with the National Cancer Institute through data transfer agreements with approvals as noted above. All methods were performed in accordance with the relevant guidelines and regulations of the respective cohorts.

### Germline variant calling and annotation

Available exome data from blood-derived samples were retrieved and analyzed from the DiscovEHR cohort to identify rare germline variants in our genes of interest. Details of the bioinformatics pipeline for variant alignment and calling used in this study have been previously published [35, 36]. Predetermined filtering criteria applied to UKBB exome data were used as described [30, 32, 33]. Exome data from gnomAD v3.1.2 was obtained directly through the consortium website with variant level detail only.

### Variant filtering and curation (Supplementary Fig. S1)

Variant curation was restricted to exonic nonsynonymous or splice single nucleotide variants (SNV) and small insertions/deletions with MAF < 0.01 in gnomAD_exome non-cancer for any subpopulation. Only variants with a VAF ≥ 0.35 and ≤ 0.65 were included to minimize the risk of including somatic variants, and all variants had a genotype quality (GQ) > 30. No homozygous or compound heterozygous variants were identified. For *ANKRD26*, the 5’UTR variants was sufficiently covered, but no variants in this region identified. For *GATA2*, the known intronic regulatory variants were not sufficiently covered for variant calling.

Variant annotation with in silico prediction tools have been published previously [37, 38]. Here, variants were annotated with five in silico tools that predict deleteriousness, including CADD ( > 20) [39], MetaSVM (D) [40], REVEL ( > 0.5) [41], BayesDel (D) [42], and Eigen ( > 0) [43]. Variants with a ClinVar (https://www.ncbi.nlm.nih.gov/clinvar/, downloaded 2/27/2022) or InterVar (https://wintervar.wglab.org) designation of uncertain significance (VUS) or pathogenic and likely pathogenic (P/LP) that had in silico values that suggest deleteriousness in ≥ 3 of 5 tools were further curated as described below. VUS with no evidence of deleteriousness by in silico tools were excluded.

For all cohorts, we defined pathogenic (P) and likely pathogenic (LP) variants as variants predicted to be P/LP by ClinVar or InterVar. Deleterious variants of uncertain significance (dVUS) are defined as VUS in ClinVar, and/or predicted to affect splicing by two or more of: Spidex (https://www.openbioinformatics.org/annovar/spidex_download_form.php), Splice AI (https://spliceailookup.broadinstitute.org), and Human Splicing Finder (https://www.genomnis.com/access-hsf), and/or missense variants predicted as deleterious by three or more in silico prediction tools (Supplementary Figure S1). The remaining VUSs plus ClinVar or InterVar benign (B) and likely benign (LB) variants were not used in the analyses.

From DiscovEHR, dVUS variants were manually curated involving review of databases including the ClinGen registry [44], dbSNP (https://www.ncbi.nlm.nih.gov/snp/), PubMed (https://pubmed.ncbi.nlm.nih.gov), HGMD [45], VarSome [46], LitVar [47], and COSMIC [48] to determine if any dVUS could be upgraded to P/LP. No dVUS had literature or database evidence which would re-classify it to P/LP. Thus, ultimately, only P/LP variants were included in the phenotype analysis; dVUSs were investigated for prevalence only. See Supplementary Table S1 for variant details.

### Population-based cohorts phenotype analysis

For individuals with a P/LP variant within DiscovEHR or UKBB, International Classification of Diseases (ICD) editions 10-CM, nine and ten codes for HM were reviewed from the electronic health record (EHR) and tumor registry. All ICD codes for hematological malignancies were included in the HM group. A subset of the HM codes was designated with myeloid malignancy (MM) codes for analyses. Supplementary Table S2 shows the ICD-9 and ICD-10 codes included. Individuals with more than one hematological malignancy-associated ICD code were manually reviewed and reconciled. Diagnoses of the same malignancy within five years were considered a single incidence of disease. If multiple diagnoses were present greater than five years apart and not coded as relapse, the individual was considered to have two separate malignancies. The GHS tumor registry has been maintained since the 1940s and contains curated malignant tumor diagnoses for all patients diagnosed at GHS facilities, and for patients who were diagnosed outside the GHS, but then had treatment within GHS. The UKBB cancer registry data is abstracted from the UK national cancer registry from information received from separate regional cancer centers (https://biobank.ndph.ox.ac.uk/showcase/label.cgi?id=100092). The completeness of this data is dependent on the individual centers.

### Statistical and survival analysis

gMMP individuals in DiscovEHR and UKBB were defined as those having a P/LP variant in one of the eight gMMP genes meeting quality metrics and are referred to as heterozygotes. All other individuals in these cohorts were considered controls (non-heterozygotes), excluding those with a gMMP variant of poor VAF and/or GQ; these were removed entirely from the analyses.

Confidence intervals for the prevalence and penetrance frequencies were generated using the Test of Equal or Given Proportions (prop.test) in RStudio v 4.1.2 for gMMP (https://www.rdocumentation.org/packages/stats/versions/3.6.2/topics/prop.test). Penetrance estimated by odds ratios were determined by logistic regression comparing those in the gMMP group to the non-heterozygote group and adjusted for age, sex, body mass index (BMI), and smoking status using SAS Enterprise Guide v 8.3 (8.3.0.103). Adjusted odds ratios are shown. The Kaplan-Meier survival analyses was used to estimate overall survival and the age-dependent penetrance of HM or MM in gMMP heterozygotes. Log-rank test for equality was used to compare statistical differences between the groups. Cox regression models were used to calculate hazard ratios for univariate and multivariate analyses adjusting for reported race, sex, smoking history, BMI, and the earliest recorded age of encounter in EHR was used (DiscovEHR) or age (UKBB). Adjusted Cox proportional hazard ratios are reported. Follow-up time started at the date of study entry and ended at death, censoring at date of last follow-up or end of study on January 1, 2023. For UKBB Biobank Kaplan-Meier (KM) time-to-event analyses, age at cancer diagnosis was obtained from field 40008. For DiscovEHR KM, only diagnoses three months after the first encounter in EHR were included in KM analyses. The last age of encounter was used as event age for censored subjects.

## Results

The population-based cohort characteristics for heterozygotes (individuals with a germline P/LP variant who may be at increased risk for a myeloid or hematologic malignancy) and non-heterozygotes (controls, individuals without a germline P/LP variant) with a hematological malignancy (HM) and myeloid malignancy (MM) were identified in the Geisinger MyCode DiscovEHR cohort (*n* = 170,503) and United Kingdom BioBank (UKBB) (*n* = 469,595) (see Methods, Supplementary Fig. S1 and Table 1). These eight genes have an autosomal dominant inheritance pattern. All variants identified are listed in Supplementary Table S1 and shown in Supplementary Fig. S2. There were 283 and 1049 heterozygotes in DiscovEHR and UKBB, respectively, and 68 and 157 P/LP variants, respectively (Supplementary Table S3). When comparing heterozygotes and non-heterozygotes, individuals from both groups in DiscovEHR and in UKBB with an HM and/or MM were significantly more likely to be deceased (Table 1). We identified gMMP variants that were present in DiscovEHR and UKBB and determined if they were also present in gnomAD (Supplementary Table S1). Current mean age of participants of both cohorts is older, 58 years for DiscovEHR and 71 years for UKBB. For DiscovEHR, the EHR data is both retrospective and prospective from the time of sample draw; all health data is accessible from the time the person entered the Geisinger Health System, irrespective of the time of sample draw.

**Table 1:** Demographics of the population-based cohorts, (A) DiscovEHR and (B) UKBB.

A	DiscovEHR								
	All Individuals			Individuals with a Hematological Malignancy			Individuals with a Myeloid Malignancy		
Risk Factors	Non-heterozygote	Heterozygote	*p*	Non-heterozygote	Heterozygote	*p*	Non-heterozygote	Heterozygote	*p*
	*n* = 170,147	*n* = 283		*n* = 4215	*n* = 11		*n* = 1018	*n* = 7	
Male, *n* (%)	67004 (39)	99 (35)	0.13	2230 (53)	8 (73)	0.19	545 (54)	5 (71)	0.46
Smoking, *n* (%)	96998 (57)	166 (59)	0.56	2623 (62)	7 (64)	1	660 (65)	5 (71)	1
Deceased, *n* (%)	18848 (11)	43 (15)	0.03	1403 (33)	8 (73)	**0.0088**	436 (43)	6 (87)	**0.047**
Current Age, mean (sd)	58.4 (19)	56.9 (19)	0.16	69.7 (15)	70.1 (13)	0.93	71.2 (14)	70.2 (15)	0.86
BMI, mean (sd)	31.0 (7.8)	31.8 (8.5)	0.11	29.2 (7.3)	28.3 (6.3)	0.68	28.6 (7.3)	29.6 (6.6)	0.72

B	UK Biobank								
	All Individuals			Individuals with a Hematological Malignancy			Individuals with a Myeloid Malignancy		
Risk Factors	Non-heterozygote	Heterozygote	*p*	Non-heterozygote	Heterozygote	*p*	Non-heterozygote	Heterozygote	*p*
	*n* = 468,265	*n* = 1049		*n* = 9497	*n* = 55		*n* = 2995	*n* = 39	
Male, *n* (%)	214613 (46)	491 (47)	0.53	5315 (56)	35 (64)	0.31	1645 (55)	29 (74)	0.024
Smoking, *n* (%)	281163 (60)	614 (58)	0.35	5969 (62)	34 (62)	0.99	1982 (66)	25 (64)	0.92
Deceased, *n* (%)	34978 (7.4)	112 (11)	**0.000098**	2960 (31)	30 (54)	**0.00034**	1084 (36)	28 (72)	**0.000010**
Current Age, mean (sd)	70.9 (8.0)	71.1 (8.0)	0.59	73.1 (7.4)	73.2 (6.9)	0.87	73.0 (7.5)	72.9 (6.3)	0.91
BMI, mean (sd)	27.4 (4.8)	27.3 (4.4)	0.40	27.8 (4.8)	27.6 (4.6)	0.72	27.9 (4.9)	26.7 (4.2)	0.09

Information is grouped for all heterozygotes and non-heterozygotes, and those with a hematological malignancy and those with a myeloid malignancy. Bolded *p*-values denote significant differences between heterozygotes and non-heterozygotes, *p* < 0.05 by Fisher’s exact test. *n* number, *BMI* Body mass index, *sd* standard deviation.

**Fig. 1:**
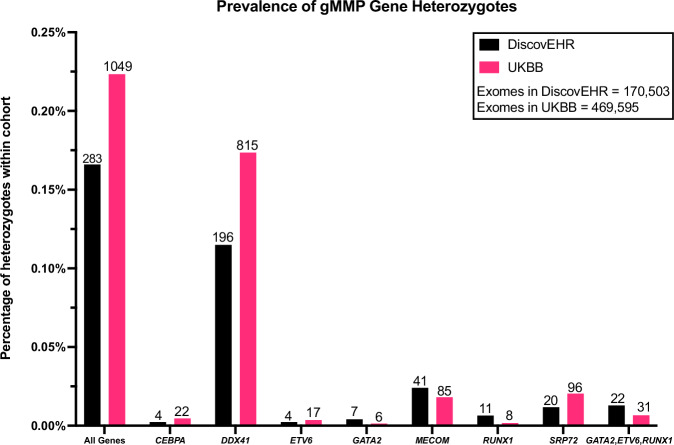
Estimated prevalence of germline myeloid malignancy predisposition genes in DiscovEHR (black) and UKBB (pink). Absolute number of heterozygotes listed per gene in each cohort above bar. Ratios, 95% confidence intervals and *p*-values are listed in Supplementary Table S4 and frequencies in Supplementary Table S5.

**Fig. 2:**
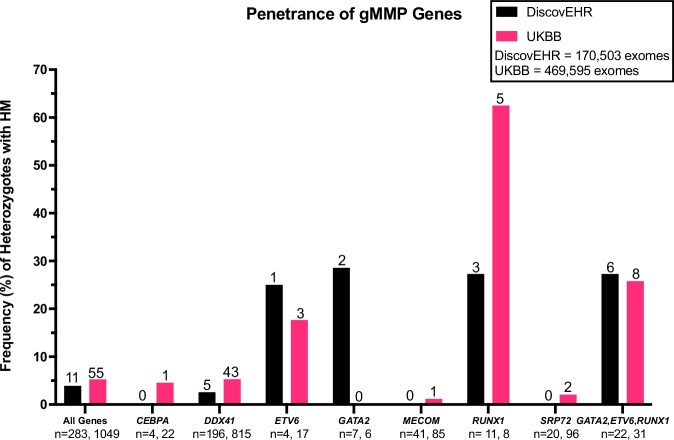
Estimated penetrance for hematological malignancies in heterozygotes of germline pathogenic/likely pathogenic myeloid malignancy predisposition gene variants in DiscovEHR (black) and UKBB (pink) is shown. Penetrance was estimated as the frequency (percentage) of HM ever recorded in heterozygote individuals in the electronic health record and/or the tumor registry. Absolute number of heterozygotes with a hematological malignancy listed per gene in each cohort above the bar. Below each bar is the number of heterozygotes for each cohort per gene/gene group. Ratios, 95% confidence intervals and *p*-values are listed in Supplementary Table S4.

Prevalence of P/LP variants in gMMP genes (Fig. 1) and HM penetrance (Fig. 2) were determined for both DiscovEHR and UKBB cohorts. Overall, there was a 1 in 448 to 1 in 602 chance of being a heterozygote for any of these gMMP P/LP variants (Supplementary Table S4). Malignancies were studied in two groups: all hematological malignancies (HM), and as well as myeloid malignancies (MM) as a subgroup which included myelodysplastic syndromes and acute myeloid leukemias (Supplementary Table S2). It was known that these genes increased MM, but we were also interested to determine if risk of other HMs was elevated in heterozygotes of these genes. Overall, grouping all genes together, the prevalence was low: 0.17–0.22% across both cohorts. However, the penetrance of the gMMPs was high, with between 1 in 19 to 1 in 26 heterozygotes having a hematological malignancy (3.8–5.2%) (Supplementary Tables S4 and S5). If the hematopoietic transcription factors genes (*GATA2*, *RUNX1*, *ETV6*, *CEBPA*, *MECOM*) are grouped together, the penetrance is higher (1 in 11 to 1 in 14). Limiting to *GATA2*, *ETV6*, and *RUNX1* – penetrance is high at 1 in 4 heterozygotes having a HM. The hematological malignancies identified are listed in Supplementary Table S6 and are categorized by gene.

Three genes show low prevalence but high penetrance in DiscovEHR and UKBB: *GATA2*, *ETV6* and *RUNX1* (Figs. 1 and 2). Conversely, *DDX41* shows higher prevalence but lower, albeit still elevated, penetrance in both DiscovEHR and UKBB (heterozygotes: DiscovEHR *n* = 196, UKBB *n* = 815; heterozygotes with HM: DiscovEHR *n* = 5, UKBB *n* = 43; prevalence: DiscovEHR 1:870, UKBB 1:576; penetrance: DiscovEHR 1:39 [2.6%], UKBB 1:19 [5.2%]) (See Supplementary Table S4 for 95% CIs and p values). In DiscovEHR there were 196 *DDX41* heterozygotes, and of these 72 (37%) were male, and in UKBB 367 of 815 were male (45%). None of the *DDX41* variants typically enriched in populations of Japanese, Korean and Chinese ancestry (p.E256K, p.Y259C, p.A500fs, p. V152G, p.E7Ter, c.935+4A>T, p.T360Ifs) were observed in either cohort. There were 67 *DDX41* P/LP variants identified, and the majority (60/67) were truncating. In DiscovEHR, of the *DDX41* heterozygotes, five had a hematological malignancy and four (80%) of those were male. Of the five *DDX41* heterozygotes with HM, three were lymphomas (non-Hodgkin, Mantle cell, diffuse large B cell).

Notably, *CEBPA* shows low prevalence and penetrance in both population cohorts. There were 13 *CEBPA* P/LP variants identified, seven in the N terminus and five in the C terminus. No *CEBPA* heterozygous individuals in DiscovEHR had HM, and in UKBB 1 in 22 heterozygotes had HM. No *ANKRD26* P/LP variants were identified within the 5’ UTR regulatory region, which is the previously described mechanism of disease for this gene. Thus, no phenotype analyses were done for *ANKRD26*. *MECOM* and *SRP72* heterozygotes were more common than the hematopoietic transcription factor group, but less common than *DDX41*. While HM has been reported (albeit rarely) in patients with *MECOM* germline P/LP variants, in these cohorts only one individual in UKBB had an HM (an AML) and none in DiscovEHR. *SRP72* was similar with only two individuals having non-myeloid HM in UKBB and none in DiscovEHR.

We also determined the number of individuals in DiscovEHR harboring VUSs with some level of deleteriousness based on in silico and literature/database evidence (dVUS, Supplementary Figure S1), but not meeting ClinVar or InterVar criteria (see Methods for details). Interestingly, there are a substantial number of individuals in DiscovEHR with those variants (DiscovEHR *n* = 2896, 1:59, 1.7% of the cohort) (Supplementary Table S2). No DiscovEHR variants’ classification was promoted to P/LP upon manual curation; all remained dVUS.

**Fig. 3:**
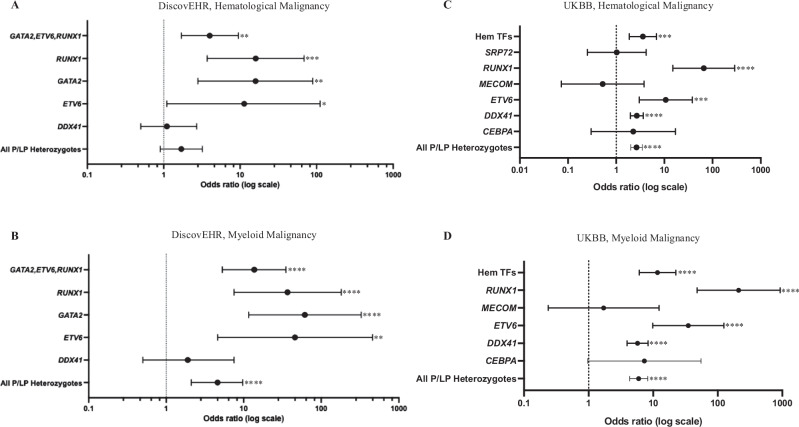
Risk of hematological and myeloid malignancy development in heterozygotes of germline pathogenic/likely pathogenic myeloid malignancy predisposition gene variants in both cohorts. The DiscovEHR cohort is shown in **A**, **B** and UKBB cohort in **C**, **D**. Logistic regression odds ratios (OR) and 95% confidence intervals were adjusted for age, sex, body mass index (BMI) and smoking. For MM in DiscovEHR *ETV6* is adjusted for sex, BMI and smoking only. Hem TFs, hematological transcription factors: *RUNX1*, *GATA2*, *CEBPA*, *ETV6*, *MECOM*. No *MECOM* heterozygotes in DiscovEHR had hematological malignancy, and only 1 in UKBB. *p ≤ 0.05; **p ≤ 0.01; ***p ≤ 0.001; and ****p ≤ 0.0001.

Cross-sectional analyses showed an increased risk of MM in both DiscovEHR and UKBB and an increased risk of HM in UKBB across all P/LP heterozygotes, demonstrated by adjusted odds ratios (ORs) (Fig. 3). For all genes combined, the adjusted ORs for HM in DiscovEHR were 1.7 [95%CI 0.90–3.2, *p* = 0.084]. Additionally, in DiscovEHR, significantly increased adjusted ORs were observed for risk of HM and MM development of G*ATA2*, *ETV6* and *RUNX1* heterozygotes individually and combined (HM OR 4.0 [95%CI 1.7–9.5, *p* = 0.0016], MM OR 14 [95%CI 5.3–35, *p* < 0.0001]) (Fig. 3A, B). In DiscovEHR, *DDX41* did not have an increased OR for HM or MM (Fig. 3B).

In UKBB, a significantly increased risk for developing an HM and MM exists for heterozygotes of all genes (HM OR 2.6 [95%CI 2.0–3.5, *p* = 3.6 × 10^−^^12^]) (Fig. 3C, D). *DDX41, RUNX1*, and *ETV6* heterozygotes were also identified as having an increased risk of HM and MM development (HM OR 2.7, 66, 11 and MM OR 5.7, 210, 35 respectively). Similar to DiscovEHR, the group of all hematopoietic transcription factors have an increased OR of 3.6 for HM and 12 for MM.

**Fig. 4:**
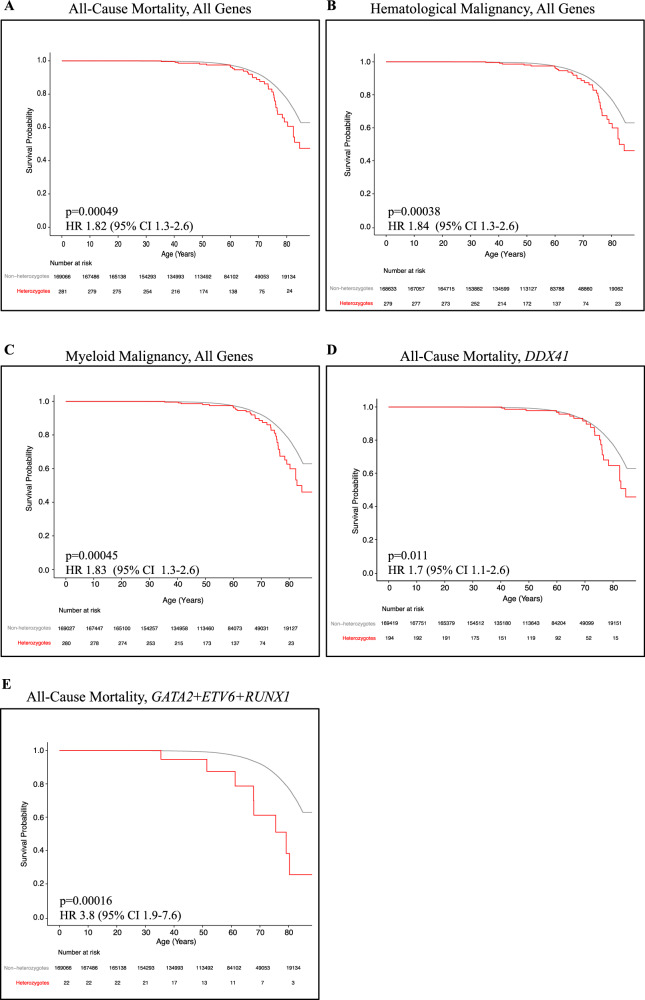
Kaplan-Meier overall survival of heterozygotes (red) and non-heterozygotes (gray) in DiscovEHR. Curves for heterozygotes and non-heterozygotes of all genes for all-cause mortality (**A**), all genes for individuals with hematological malignancy (**B**), all genes for individuals with a myeloid malignancy (**C**), *DDX41* heterozygotes for all-cause mortality (**D**) and all-cause mortality for the combined group *GATA2*, *ETV6* and *RUNX1* (**E**). Adjusted Cox proportional hazards and log-rank *p*-values compared the heterozygote curve to non-heterozygote curve. *p* < 0.05. HR Hazard ratio, CI Confidence interval.

**Fig. 5:**
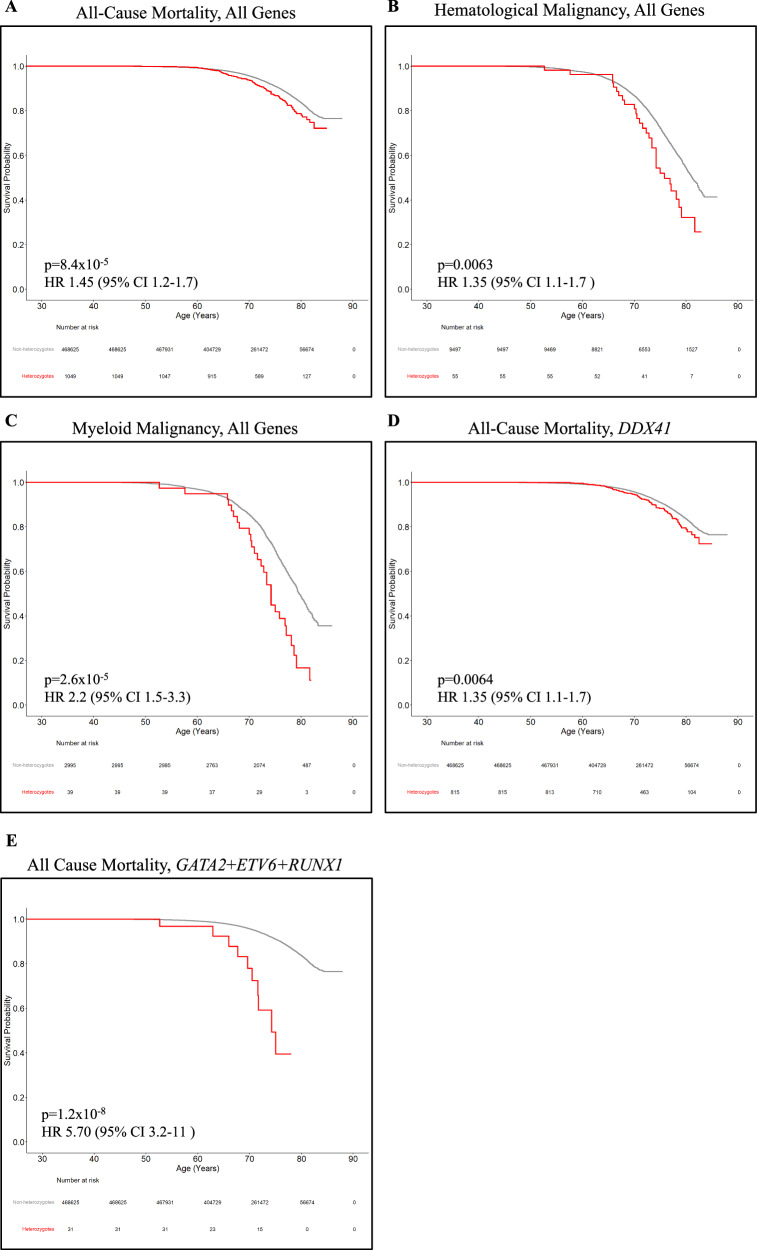
**Kaplan-Meier overall survival of heterozygotes (red) and non-heterozygotes (gray) in UKBB.** Curves for heterozygotes and non-heterozygotes of all genes for all-cause mortality (**A**), all genes for individuals with hematological malignancy (**B**), all genes for individuals with a myeloid malignancy (**C**), *DDX41* heterozygotes for all-cause mortality (**D**) and all-cause mortality for the combined group *GATA2*, *ETV6* and *RUNX1* (**E**). Adjusted Cox proportional hazards and log-rank *p*-values compared the heterozygote curve to non-heterozygote curve. *p* < 0.05. HR hazard ratio, CI confidence interval.

Time-dependent Kaplan-Meier survival analyses showed heterozygotes of P/LP germline variants in the gMMP genes of interest were statistically less likely to survive compared to non-heterozygotes (Figs. 4A, and 5A; adjusted hazard ratios (HR) are shown). This includes those with HM and MM, of which heterozygotes were statistically less likely to survive compared to non-heterozygotes in the DiscovEHR cohort (Fig. 4B, C) and in UKBB (Fig. 5B, C). In DiscovEHR, age-dependent penetrance (cumulative risk) analyses of gMMP heterozygotes yielded a significantly earlier onset for MM when combing all genes (Supplementary Figure S3). For the more penetrant hematopoietic transcription factor genes (*GATA2*, *ETV6* and *RUNX1*), there is significantly increased risk and earlier onset of HM (HR 12 [95%CI 1.9–28, *p* = 3.7 × 10^−^^8^]) and MM (HR 32 [95%CI 12–86, *p* = 4.7 × 10^−^^12^]). In UKBB, time to cancer in heterozygotes was significantly earlier for all cancers, HM, and MM (Supplementary Figure S4).

For *DDX41* heterozygotes in both cohorts, there is a significant increase in all-cause mortality (Figs. 4D, 5D), despite the lower penetrance observed. Interestingly, *DDX41* heterozygotes alone did not show an early onset of cancer in DiscovEHR (Supplementary Figure S3D), but a small increase in time to cancer in UKBB (Supplementary Figure S4D). Given the increased all-cause mortality for *DDX41* heterozygotes, we investigated the prevalence of non-hematological cancers comparing *DDX41* heterozygotes to non-heterozygote controls within the DiscovEHR. We identified a small number of excess cancers: uterine, bladder, and thyroid (heterozygotes *n* = 3, 3, 2), with a heterozygote/non-heterozygote frequency of 1.81, 1.29, 1.12, respectively.

Cumulative risk for all-cause mortality was determined across nine decades of life. Table 2A shows the cumulative risks of all-cause mortality for gMMP heterozygotes in both cohorts. In DiscovEHR, gMMP heterozygotes had a 37% (95% CI 23–48) probability of death by age 80. The probability of death from all-cause mortality for gMMP heterozygotes in the UKBB cohort was lower at 25% (95% CI 19–30) by 80 years of age. Average age-specific cumulative risk for developing a HM or MM increases at 70 years in both cohorts (Table 2B). Overall, these genetically ascertained heterozygote individuals are at higher risk for development of myeloid malignancy and all-cause mortality across all gMMP genes of interest compared to non-heterozygotes.

**Table 2:** Time-dependent cumulative risk for heterozygotes with P/LP variants in all germline myeloid malignancy predisposition genes within DiscovEHR and UKBB.

A	Cumulative Risk of Death in Heterozygotes (%) (95%CI)									
Age (years)	0	10	20	30	40	50	60	70	80	90
DiscovEHR All-cause Mortality	0 (0 to 0)	0 (0 to 0)	0 (0 to 0)	0 (0 to 0)	0.40 (0–1.3)	0.70 (0.70–0.70)	3.2 (0.60–5.7)	11 (5.4–17)	37 (23–48)	53 (35–66)
UKBB All-cause Mortality	-	-	-	0 (0 to 0)	0 (0 to 0)	0.095 (0–0.028)	0.61 (0.12–1.1)	6.5 (4.7–8.4)	25 (19–30)	-
**B**	**Cumulative Risk of Malignancy Types for Heterozygotes (%) (95%CI)**									
Age (years)	0	10	20	30	40	50	60	70	80	90
DiscovEHR HM	0 (0 to 0)	0 (0 to 0)	0 (0 to 0)	0 (0 to 0)	0 (0 to 0)	0.70 (0.70–0.70)	0.50 (0–1.5)	5.2 (0.90–9.2)	5.8 (3.1–17)	10.4 (3.1–17)
DiscovEHR MM	0 (0 to 0)	0 (0 to 0)	0 (0 to 0)	0 (0 to 0)	0 (0 to 0)	0.70 (0.70–0.70)	0.50 (0–1.5)	4.4 (0.40–0.60)	5.8 (0.80–12)	6.5 (0.80–12)
UKBB HM	0 (0 to 0)	0 (0 to 0)	0 (0 to 0)	0 (0 to 0)	0 (0 to 0)	0.29 (0–0.61)	0.68 (0.18–1.2)	3.5 (2.1–4.8)	8.0 (5.4–11)	-
UKBB MM	0 (0 to 0)	0 (0 to 0)	0 (0 to 0)	0 (0 to 0)	0 (0 to 0)	0.095 (0–0.28)	0.19 (0–0.46)	2.4 (1.3–3.5)	5.6 (3.4–7.8)	-

Total risk is stratified by decade of life for (A) all-cause mortality (death) and (B) hematological malignancies (HM), and myeloid malignancies (MM). CI, confidence interval.

## Discussion

This study represents the first use of the genome-first approach to comprehensively estimate the population-level risk for multiple gMMP genes. We demonstrated the wide variation in prevalence that exists for eight gMMP genes. Through the use of less biased cohorts, we observed significant penetrance for *GATA2*, *ETV6*, and *RUNX1*. We note the surprising low frequencies and few hematological manifestations for *CEBPA*, a gene reported to have high germline penetrance causing early-onset AML [20, 49]. Importantly, we provide additional evidence that *DDX41* is a major driver in myeloid malignancy germline predisposition.

This study quantified the risk for hematological malignancies and overall survival in heterozygotes with a germline P/LP variant in eight gMMP genes. Notably, heterozygotes for germline P/LP variants in *GATA2, ETV6*, and *RUNX1* (both cohorts), and *DDX41* (UKBB only) have significantly increased risk for developing hematological malignancies. Overall, gMMP heterozygotes are common in the population (1:448–602), have a 1:19–26 chance of developing a hematological malignancy, and are at increased mortality risk compared to the general population. *DDX41* heterozygotes were prevalent in the population (1:576–870), with high likelihood to develop a hematological malignancy (1:19–1:39), and had an increased overall all-cause mortality. While *GATA2*, *ETV6*, and *RUNX1* variants are rare in the cohorts, heterozygotes of those variants were at increased risk of developing a hematological malignancy. For the 19 *RUNX1* heterozygotes identified, the risk is 1:2–1:4; for the 21 *ETV6* heterozygotes, the risk is 1:4–1:6; and, for the 13 *GATA2* heterozygotes, the risk is 0–1:4.

Similarities for *DDX41* frequency and penetrance values in both DiscovEHR and UKBB provide noteworthy evidence supporting *DDX41* as a major driver in myeloid malignancy germline predisposition. Recently, Kovilakam and colleagues reported on UKBB participants with *DDX41* variants [50]. They identified a higher frequency of *DDX41* heterozygotes. This difference is likely due to stricter variant classification assignment and variant allele frequency (VAF) used in our study. They similarly found a significantly increased risk for *DDX41* heterozygotes to develop a HM or MM. Further, other recent studies have indicated that *DDX41* should be considered a distinct clinical entity with distinct biology and management [21, 51–53]. Similar to those studies, we observe high frequency of genetically ascertained individuals with *DDX41* germline P/LP variants, an average older age of symptom manifestation, and the presence of well-described germline variants (e.g., p.M1I). However, in our study, we observe a low number of unique *DDX41* germline variants, and we do not observe a male predominance in heterozygotes with P/LP variants. This may be due to increased number of women who typically enrolled in clinical trials. We identified an increased frequency of uterine, bladder, and thyroid cancer in *DDX41*; however, the case numbers were small, and this finding warrants further follow-up. Most interestingly, the adjusted hazard ratio for all-cause mortality, not just hematological and myeloid malignancy mortality, was remarkably consistent between the UKBB and DiscovEHR *DDX41* heterozygotes. This novel finding suggests the need for clinically unaffected *DDX41* heterozygotes to be brought to medical attention proactively, given the increased risk of mortality. The if and how to monitor these heterozygotes is not clear and a subject of debate amongst experts. Prospective natural history studies of these patients in the future will help clarify this risk, as has been done successfully for inherited bone marrow failure syndromes.

*SRP72* has been proposed as a bone marrow failure and gMMP predisposition gene, however, we did not find evidence in these cohorts. In the original families described [17], one family had a missense variant and the other was a truncating variant that removed the 7SL RNA binding domain. In the current two cohorts, the P/LP heterozygotes identified all had truncating variants, which removed all or some of that domain. Of the 116 heterozygotes we identified, none had a myeloid malignancy. Further research and family discovery will be needed to discern if germline P/LP variants in *SRP72* truly cause myeloid malignancy predisposition. Similarly, the risk of MM amongst the previously described *MECOM* heterozygotes is small; here, we observed only a very small risk of MM.

Important strengths of our study include the use of automated tools for annotation with secondary manual curation, allowing for a stringent and conservative curation. There were 68 and 157 P/LP variants in DiscovEHR and UKBB, respectively, as well as 620 dVUSs in DiscovEHR (Supplementary Table S3). These values are drastically different and indicate a need for improved variant curation strategies and more functional evidence to classify variants of uncertain significance (VUS) as pathogenic or benign. Variants with at least three positive in silico scores were further investigated to see if literature evidence existed to change any particular variant’s classification. Interestingly, none of these variants were promoted to P/LP. Functional laboratory evidence and family segregation data or additional families would be required. These numbers provide support that our methodology prevented the overestimation of the prevalence of these disorders. It also highlights the importance of the ACMG/AMP variant curation guidelines [54]. Inclusion of the dVUS would result in a frequency of 1.9% for myeloid malignancy germline predisposition (DiscovEHR P/LP+dVUS), which is much higher than sickle cell disease (0.27%), a considerably more common hematological disorder than myeloid malignancy predisposition (Supplementary Table S2).

The age of the individuals studied leads to a critical bias in this study. The low penetrance observed for *CEBPA* heterozygotes is notable and likely represents some heterozygotes being deceased before the mean age of enrollment. This likely underestimates the prevalence and penetrance for this gene, and other genes studied here. Further, there are very few differences observed in the current age or age at HM or MM diagnosis in both cohorts (Supplementary Table S7) indicating that we may only be sampling those with HM later in life as these individuals have had time to enroll and develop disease. In both DiscovEHR and UKBB, the age range of HM or MM diagnoses is slightly higher in heterozygotes than in non-heterozygotes. This similarly reflects a suspected left truncation of individuals with germline disease and the documented healthy volunteer bias in UKBB. In the time to cancer analysis (Supplementary Fig. S3C and S4C), there is a significantly earlier onset of myeloid malignancy when grouping all genes together. Of note *DDX41*, which uniquely predisposes to MDS at the typical time of onset for sporadic MDS, does not have an earlier onset in the time to all cancer analysis performed for DiscovEHR (Supplementary Fig. S3D). This bias leads to inclusion of more heterozygotes without disease or those with lower penetrance variants in this study, thus underestimating the contribution of germline disease to myeloid predisposition. The data presented here strengthens the growing understanding that there is a sizeable contribution of germline predisposition to myeloid malignancy both in the pediatric and adult populations.

There are additional limitations to this study. These eight genes were chosen from the literature, but there are more genes that convey hematological malignancy risk, including newly described genes which will be important for future research. As previously described, DiscovEHR is a clinical population cohort while UKBB is a volunteer population cohort with a documented healthy volunteer bias [55]. Additionally, samples from both population cohorts are peripheral blood, which raises concerns of clonal hematopoiesis. To account for this, we limited all variants to very strict VAF values and cross-referenced variants in COSMIC (https://cancer.sanger.ac.uk/cosmic) to call germline variants more confidently and reduce the probability of somatic variant contamination. However, VAF is a poor tool for determining germline status, thus it is possible that heterozygote frequencies reported are an overestimate, if somatic variants have inadvertently been included. These large cohorts were ascertained before the current understanding of clonal hematopoiesis’ influence on germline calling was appreciated. In the future, it will be very informative to repeat these analyses using skin or hair bulb samples collected on similarly large cohorts.

Additionally, the variant interpretation used here was modified from the original American College of Medical Genetics/Association for Molecular Pathology (ACMG/AMP) guidelines for sequence variant interpretation [55] to be appropriate for a population-level data study, which lacks information on family history and inheritance pattern. While we did evaluate the 5’UTR of *ANKRD26*, intragenic insertions and deletions, copy number variants (CNV), and intronic variants were not assessed. This is a limitation particularly for *GATA2*, in which there are known pathogenic noncoding variants in an intronic enhancer region, which account for an estimated 10% of P/LP variants [56]. This region was not sufficiently covered by the exome sequencing used; thus, it is likely that our estimation of *GATA2* heterozygosity prevalence is underestimated. Germline CNVs have also been reported for *DDX41* [57], *ETV6* [22]*, MECOM* [58–60], and *RUNX1* [61]; thus these prevalences may also be underestimated. Lastly, cancer diagnoses were taken from EHR and cancer registry data which can have gaps, and thus may underestimate the cancers rates observed in the heterozygotes.

The data presented here provides evidence to better define this increasingly acknowledged group of syndromes and inform surveillance recommendations for myeloid malignancies. Population-level predisposition data could allow clinicians to optimally manage patients and at-risk family members with P/LP germline variants, notably when screening family members of a proband and planning an allogeneic hematopoietic cell transplant. The choice of transplant preparative regimens, proper genetic counseling and cancer surveillance for affected family members, and possible variant-informed therapeutic interventions are all advised by germline genetics and could define how MDS/AML are treated through precision medicine.

In conclusion, we utilized the genome-first approach to identify genetically-ascertained individuals with an gMMP P/LP variant within population cohorts. Germline P/LP variants in *GATA2*, *ETV6* and *RUNX1* predispose heterozygotes to increased risk for developing hematological malignancies, early malignancy (as a group), and increased all-cause mortality for *RUNX1*. This increased risk of malignancy and death adds support to the proposed addition of *RUNX1* to the ACMG/AMP secondary findings list, as identification of these variants in patients would lead to increased surveillance and better clinical management. We feel that inclusion of *GATA2* should also be considered as this data is likely underestimating the risk of myeloid malignancy and death in individuals with heterozygous *GATA2*, given the age bias present. This study represents the first attempt to comprehensively estimate the population-level hematological predisposition risk for multiple gMMP genes, demonstrating that the genome-first approach presents a unique opportunity to effectively determine hereditary myeloid malignancy risk in the general population.

## Supplementary information

Supplementary File A

Supplementary File B

## Data Availability

Variant level data is provided in Supplementary Table S1. The data generated through the DiscovEHR cohort are available upon request from the corresponding author as allowed by institutional review board. Data from gnomAD used in this study are publicly available at https://gnomad.broadinstitute.org. The data used from the United Kingdom BioBank used in this study are available at https://www.ukbiobank.ac.uk.
