# Supplementary material for: Genome-first determination of the prevalence and penetrance of eight germline myeloid malignancy predisposition genes: a study of two population-based cohorts
Source: Leukemia. 2024 Nov 6;39(2):400–11. doi: 10.1038/s41375-024-02436-y (PMC11794151; doi:10.1038/s41375-024-02436-y)
Supplement: Supplementary file 1 — Supplementary File A [file 41375_2024_2436_MOESM1_ESM.pdf]

CEBPA P/LP	ENST00000498907.2				Number of heterozygotes / Found in gnomAD		
**Unique ID**	**HGVS c.**	**HGVS p.**	**Pathogenicity**	**Reason for Pathogenicity Call**	**DiscovEHR**	**UKBB**	**gnomAD (Y/-)**
chr19_33301481_G_A	c.934C>T	p.Gln312Ter	LP	InterVar Likely Pathogenic	2	0	-
chr19_33301523_T_A	c.892A>T	p.Lys298Ter	LP	InterVar Likely Pathogenic	0	1	-
chr19_33301946_GCCGC_G	c.465_468del	p.Pro157TrpfsTer2	LP	InterVar Likely Pathogenic	0	3	-
chr19_33302091_G_C	c.324C>G	p.Tyr108Ter	P	ClinVar Pathogenic	0	1	-
chr19_33302411_C_A	c.4G>T|	p.Glu2Ter	LP	InterVar Likely Pathogenic	0	1	-
chr19_33302423_T_TA	c.96dup	p.Asn33Ter	LP	InterVar Likely Pathogenic	1	15	-
chr19_33302432_C_A	c.88G>T	p.Glu30Ter	LP	InterVar Likely Pathogenic	0	1	-
chr19_33302477_G_A	c.43C>T	p.Arg15Ter	LP	InterVar Likely Pathogenic	1	0	-

DDX41 P/LP	ENST00000330503.11				Number of heterozygotes / Found in gnomAD		
Unique ID	HGVS c.	HGVS p.	Pathogenicity	Reason for Pathogenicity Call	DiscovEHR	UKBB	gnomAD (Y/-)
chr5_177511822_TCC_T	c.1836_1837del	p.Asp613fs	LP	InterVar Likely Pathogenic	0	1	-
chr5_177512162_T_A	c.1720A>T	p.Lys574Ter	P	InterVar Pathogenic	0	1	-
chr5_177512207_C_CT	c.1622-2dup	-	LP	InterVar Likely Pathogenic	0	4	-
chr5_177512355_CTG_C	c.1640_1641delCA	p.Thr547fs	P	ClinVar Pathogenic	0	20	-
chr5_177512362_T_TC	c.1634dupG	p.Asn546fs	LP	InterVar Likely Pathogenic	0	1	-
chr5_177512570_G_GC	c.1528dupG	p.Ala510fs	LP	ClinVar Likely Pathogenic	0	1	-
chr5_177512647_T_C	c.1400-2A>G	-	P	InterVar Pathogenic	0	1	-
chr5_177512784_GC_G	c.1394del	p.Gly465AlafsTer22	LP	InterVar Likely Pathogenic	1	0	-
chr5_177512824_AG_A	c.1354del	p.Leu452CysfsTer9	LP	InterVar Likely Pathogenic	2	1	-
chr5_177513014_CG_C	c.1352delC	p.Pro451fs	LP	InterVar Likely Pathogenic	0	1	-
chr5_177513078_A_ACC	c.1233_1234dup	p.Val412GlyfsTer2	LP	InterVar Likely Pathogenic	0	1	-
chr5_177513355_G_A	c.1228C>T	p.Gln410Ter	P	InterVar Pathogenic	0	2	-
chr5_177513396_A_G	c.1241T>C	p.Ile414Thr	LP	ClinVar Likely Pathogenic	0	41	Y
chr5_177513401_CA_C	c.1181del	p.Val394GlyfsTer19	LP	InterVar Likely Pathogenic	1	0	-
chr5_177513407_CTT_C	c.1174_1175del	p.Lys392AlafsTer66	LP	InterVar Likely Pathogenic	1	0	-
chr5_177513442_T_A	c.1195A>T	p.Lys399Ter	P	ClinVar Pathogenic	0	1	-
chr5_177513471_GTC_G	c.1164_1165delGA	p.Gln388fs	LP	InterVar Likely Pathogenic	0	1	-
chr5_177513475_G_A	c.1108C>T	p.Gln370Ter	P	InterVar Pathogenic	1	1	-
chr5_177513715_AC_A	c.1067del	p.Gly356ValfsTer36	LP	InterVar Likely Pathogenic	0	1	-
chr5_177513796_CT_C	c.986del	p.Gln329ArgfsTer7	LP	InterVar Likely Pathogenic	1	0	-
chr5_177513798_G_A	c.1039C>T	p.Gln347Ter	P	InterVar Pathogenic	0	4	-
chr5_177513835_CAT_C	c.1000_1001delAT	p.Met334fs	P	ClinVar Pathogenic	0	73	Y
chr5_177513848_C_G	c.936-1G>C	-	P	InterVar Pathogenic	0	1	-
chr5_177514705_G_A	c.931C>T	p.Arg311Ter	P	InterVar Pathogenic	3	0	Y
chr5_177514720_G_A	c.916C>T	p.Gln306Ter	P	ClinVar Pathogenic	1	0	-
chr5_177514749_AG_A	c.940delC	p.Leu314fs	LP	InterVar Likely Pathogenic	0	1	-
chr5_177514776_T_TC	c.913dupG	p.Asp305fs	LP	InterVar Likely Pathogenic	0	1	-
chr5_177514831_GC_G	c.804del	p.Glu268AspfsTer36	LP	InterVar Likely Pathogenic	1	0	-
chr5_177514918_A_AG	c.795dup	p.Ser266LeufsTer82	LP	InterVar Likely Pathogenic	0	3	-
chr5_177514941_G_A	c.773C>T	p.Pro258Leu	LP	ClinVar Likely Pathogenic	0	1	Y
chr5_177514978_GT_G	c.789delA	p.Glu263fs	LP	InterVar Likely Pathogenic	0	1	-
chr5_177514994_G_GC	c.719_720insG	p.Ile240MetfsTer108	LP	InterVar Likely Pathogenic	0	1	-
chr5_177515050_TC_T	c.663del	p.Met221IlefsTer2	LP	InterVar Likely Pathogenic	0	1	-
chr5_177515065_A_G	c.649T>C	p.Ser217Pro	LP	ClinVar Likely Pathogenic	0	19	-
chr5_177515066_T_TA	c.647dup	p.Ser217IlefsTer4	LP	InterVar Likely Pathogenic	0	2	Y
chr5_177515185_C_T	c.644+1G>A	-	P	InterVar Pathogenic	0	1	Y
chr5_177515202_G_A	c.628C>T	p.Gln210Ter	P	InterVar Pathogenic	1	0	-
chr5_177515208_G_A	c.622C>T	p.Gln208Ter	P	InterVar Pathogenic	0	1	-
chr5_177515238_T_A	c.646A>T	p.Lys216Ter	P	InterVar Pathogenic	0	1	-
chr5_177515685_C_T	c.625G>A	p.Ala209Thr	LP	ClinVar Likely pathogenic	0	2	-
chr5_177515739_C_T	c.517G>A	p.Gly173Arg	LP	ClinVar Likely Pathogenic	0	37	Y
chr5_177515781_G_A	c.529C>T	p.Arg177Ter	P	ClinVar Pathogenic	0	4	-
chr5_177515807_CG_C	c.502delC	p.Arg168fs	LP	InterVar Likely Pathogenic	0	1	-
chr5_177515822_177515823delinsTG	c.435-2_435-1delAGinsCA	-	P	ClinVar Pathogenic	0	14	

chr5_177515822_C_T	c.435-1G>A	-	P	InterVar Pathogenic	0	3	-
chr5_177515928_C_G	c.434+1G>C	-	LP	ClinVar Likely Pathogenic	0	5	-
chr5_177515944_T_TCATC	c.415_418dup	p.Asp140GlyfsTer2	P	ClinVar Pathogenic	71	222	Y
chr5_177515953_G_GT	c.409dup	p.Thr137AsnfsTer3	LP	InterVar Likely Pathogenic	0	13	-
chr5_177516122_G_A	c.424C>T	p.Arg142Ter	P	InterVar Pathogenic	0	4	-
chr5_177516128_C_A	c.364G>T	p.Glu122Ter	P	ClinVar Pathogenic	10	0	Y
chr5_177516154_T_TCTTC	c.334_337dup	p.Glu113GlyfsTer23	LP	InterVar Likely Pathogenic	1	0	-
chr5_177516167_G_A	c.379C>T	p.Gln127Ter	P	InterVar Pathogenic	0	2	-
chr5_177516168_CT_C	c.323del	p.Lys108SerfsTer3	P	ClinVar Pathogenic	2	14	-
chr5_177516179_CAG_C	c.365_366delCT	p.Ser104CysfsTer30	LP	InterVar Likely Pathogenic	0	1	-
chr5_177516182_ACT_A	c.362_363delAG	p.Glu121fs	LP	InterVar Likely Pathogenic	0	1	-
chr5_177516185_CTT_C	c.305_306del	p.Lys102ArgfsTer32	P	ClinVar Pathogenic	3	5	Y
chr5_177516429_G_A	c.211C>T	p.Arg71Ter	P	InterVar Pathogenic	0	3	-
chr5_177516430_C_CT	c.155dup	p.Arg53AlafsTer16	P	InterVar Pathogenic	1	15	-
chr5_177516444_G_A	c.142C>T	p.Gln48Ter	P	InterVar Pathogenic	2	9	Y
chr5_177516733_G_A	c.130C>T	p.Gln44Ter	P	InterVar Pathogenic	0	4	-
chr5_177516742_G_A	c.121C>T	p.Gln41Ter	P	ClinVar Pathogenic	12	32	Y
chr5_177516755_A_T	c.108T>A	p.Tyr36Ter	LP	InterVar Likely Pathogenic	0	3	-
chr5_177516808_C_A	c.55G>T	p.Gly19Ter	LP	InterVar Likely Pathogenic	1	1	-
chr5_177516933_C_A	c.13G>T	p.Glu5Ter	P	InterVar Pathogenic	1	4	Y
chr5_177516935_G_T	c.11C>A	p.Ser4Ter	P	InterVar Pathogenic	0	9	Y
chr5_177516943_C_T	c.3G>A	p.Met1Ile	P	ClinVar Pathogenic/Likely Pathogenic	79	216	Y
chr5_177516944_A_C	c.2T>G	p.Met1Arg	LP	InterVar Likely Pathogenic	0	1	-

ETV6 P/LP	ENST00000396373.8				Number of heterozygotes / Found in gnomAD		
**Unique ID**	**HGVS c.**	**HGVS p.**	**Pathogenicity**	**Reason for Pathogenicity Call**	**DiscovEHR**	**UKBB**	**gnomAD (Y/-)**
chr12_11752456_C_T	c.40C>T	p.Arg14Ter	P	InterVar Pathogenic	1	2	-
chr12_11752531_C_T	c.115C>T	p.Arg39Ter	P	InterVar Pathogenic	0	3	-
chr12_11752580_G_A	c.163+1G>A	-	P	InterVar Pathogenic	0	2	-
chr12_11853483_CT_C	c.391delT	p.Ser131fs	LP	InterVar Likely Pathogenic	0	1	-
chr12_11853525_C_T	c.427C>T	p.Gln143Ter	P	InterVar Pathogenic	0	1	-
chr12_11869567_T_TC	c.613dupC	p.Leu205fs	LP	InterVar Likely Pathogenic	0	1	-
chr12_11869689_T_TCCCCCGA	c.730_insCCCCGA	p.His244Pro*58	P	InterVar Pathogenic	0	1	
chr12_11869779_CA_C	c.820del	p.Met274CysfsTer4	LP	InterVar Likely Pathogenic	1	0	-
chr12_11884471_T_TATCA	c.1037_1040dupATCA	p.Leu348fs	LP	InterVar Likely Pathogenic	0	1	-
chr12_11884487_AC_A	c.1053delC	p.Asp351fs	LP	InterVar Likely Pathogenic	0	1	-
chr12_11884510_C_T	c.1075C>T	p.Arg359Ter	P	InterVar Pathogenic	1	0	-
chr12_11884533_A_AAT	c.1101_1102dupAT	p.Phe368fs	LP	InterVar Likely Pathogenic	0	1	-
chr12_11884541_G_A	c.1106G>A	p.Arg369Gln	P	ClinVar Pathogenic	0	2	Y
chr12_11884573_T_TG	c.1142dup	p.Asn382LysfsTer3	LP	InterVar Likely Pathogenic	1	0	-
chr12_11885926_AACAGAACAAACATG_A	c.1156_1169delAGAACAAACATGAC	p.Arg386fs	LP	InterVar Likely Pathogenic	0	1	-

GATA2 P/LP	ENST00000341105.6				Number of heterozygotes / Found in gnomAD		
**Unique ID**	**HGVS c.**	**HGVS p.**	**Pathogenicity**	**Reason for Pathogenicity Call**	**DiscovEHR**	**UKBB**	**gnomAD (Y/-)**
chr3_128481119_TG_T	c.1342del	p.His448ThrfsTer29	LP	InterVar Likely Pathogenic	1	0	-
chr3_128481188_G_T	c.1274C>A	p.Ser425Ter	LP	InterVar Likely Pathogenic	3	0	-
chr3_128481276_G_A	c.1186C>T	p.Arg396Trp	LP	InterVar Likely Pathogenic	1	0	-
chr3_128481901_G_A	c.1061C>T	p.Thr354Met	P	ClinVar Pathogenic	1	2	-
chr3_128485776_C_CGGTCCCCCCA	c.812_821dup	p.Ala275GlyfsTer10	LP	InterVar Likely Pathogenic	1	0	-
chr3_128481269_C_T	c.1193G>A	p.Arg398Gln	P	ClinVar Pathogenic	0	1	-
chr3_128481300_T_C	c.1162A>G	p.Met388Val	LP	InterVar Likely Pathogenic	0	1	-
chr3_128481901_G_C	c.1061C>T	p.Thr354Met	P	ClinVar Pathogenic	0	1	-
chr3_128483889_G_A	c.988C>T	p.Arg330Ter	P	ClinVar Pathogenic	0	1	-

MECOM P/LP	ENST00000651503.2				Number of heterozygotes / Found in gnomAD		
Unique ID	HGVS c.	HGVS p.	Pathogenicity	Reason for Pathogenicity Call	DiscovEHR	UKBB	gnomAD (Y/-)
chr3_169085042_G_A	c.3587C>T	p.Ala1196Val	LP	InterVar Likely Pathogenic	0	1	Y
chr3_169085043_C_G	c.3586G>C	p.Ala1196Pro	LP	InterVar Likely Pathogenic	0	8	-
chr3_169089183_C_A	c.3402G>T	p.Arg1134Ser	LP	InterVar Likely Pathogenic	0	2	-
chr3_169090113_AATATCATT_AC	c.3280_3287delAATGATATinsG	p.Asn1094ValfsTer10	LP	InterVar Likely Pathogenic	0	1	-
chr3_169095076_C_T	c.3019G>A	p.Gly1007Ser	LP	InterVar Likely Pathogenic	4	5	Y
chr3_169095148_A_C	c.2947T>G	p.Cys983Gly	LP	InterVar Likely Pathogenic	1	0	-
chr3_169095148_A_G	c.2947T>C	p.Cys983Arg	LP	InterVar Likely Pathogenic	1	0	-
chr3_169107927_C_G	c.2603G>C	p.Trp868Ser	LP	InterVar Likely Pathogenic	0	2	-
chr3_169107931_TTCTCTGA_T	c.2592_2598del	p.Asp864GlufsTer14	LP	InterVar Likely Pathogenic	1	7	-
chr3_169107952_A_C	c.2578T>G	p.Phe860Val	LP	InterVar Likely Pathogenic	0	1	-
chr3_169112873_C_T	c.1519G>A	p.Val507Ile	LP	InterVar Likely Pathogenic	0	1	-
chr3_169115383_C_T	c.2489G>A	p.Arg830Lys	LP	InterVar Likely Pathogenic	0	1	-
chr3_169121056_A_T	c.1132T>A	p.Ser378Thr	LP	InterVar Likely Pathogenic	0	1	-
chr3_169121214_T_C	c.979-5A>G	-	P	InterVar Pathogenic	0	2	Y
chr3_169122597_A_G	c.961T>C	p.Cys321Arg	LP	InterVar Likely Pathogenic	2	8	-
chr3_169122619_A_C	c.939T>G	p.His313Gln	LP	InterVar Likely Pathogenic	0	1	-
chr3_169122716_T_A	c.842A>T	p.His281Leu	LP	InterVar Likely Pathogenic	0	1	-
chr3_169127872_A_G	c.802T>C	p.Cys268Arg	LP	InterVar Likely Pathogenic	0	1	-
chr3_169131427_A_C	c.613+2T>G	-	P	InterVar Pathogenic	0	1	-
chr3_169131484_CA_C	c.557del	p.Leu186ArgfsTer4	LP	InterVar Likely Pathogenic	0	1	-
chr3_169131495_C_G	c.547G>C	p.Glu183Gln	LP	InterVar Likely Pathogenic	0	9	-
chr3_169131498_C_T	c.544G>A	p.Gly182Arg	LP	InterVar Likely Pathogenic	0	1	-
chr3_169131501_G_A	c.541C>T	p.Pro181Ser	LP	InterVar Likely Pathogenic	1	0	-
chr3_169131507_T_C	c.535A>G	p.Ile179Val	LP	InterVar Likely Pathogenic	0	1	-
chr3_169131524_T_C	c.518A>G	p.Tyr173Cys	LP	InterVar Likely Pathogenic	0	1	Y
chr3_169131533_T_C	c.511-2A>G	-	P	InterVar Pathogenic	0	1	-
chr3_169143711_T_C	c.497A>G	p.Gln166Arg	LP	InterVar Likely Pathogenic	1	0	-
chr3_169143730_G_T	c.478C>A	p.His160Asn	LP	InterVar Likely Pathogenic	1	0	-
chr3_169143748_C_T	c.460G>A	p.Ala154Thr	LP	InterVar Likely Pathogenic	2	1	-
chr3_169143759_T_C	c.449A>G	p.Tyr150Cys	LP	InterVar Likely Pathogenic	0	2	-
chr3_169144995_A_G	c.376-1163T>C	-	P	InterVar Pathogenic	0	1	-
chr3_169381231_C_T	c.331G>A	p.Gly111Arg	LP	InterVar Likely Pathogenic	0	2	-
chr3_169381239_G_A	c.323C>T	p.Pro108Leu	LP	InterVar Likely Pathogenic	1	0	-
chr3_169381249_T_G	c.313A>C	p.Lys105Gln	LP	InterVar Likely Pathogenic	1	1	-
chr3_169381270_T_A	c.292A>T	p.Arg98Trp	LP	InterVar Likely Pathogenic	5	0	-
chr3_169381270_T_C	c.292A>G	p.Arg98Gly	LP	InterVar Likely Pathogenic	0	1	-
chr3_169381279_A_G	c.283T>C	p.Trp95Arg	LP	InterVar Likely Pathogenic	0	5	Y
chr3_169381311_T_C	c.251A>G	p.Glu84Gly	LP	InterVar Likely Pathogenic	3	11	Y
chr3_169381320_T_C	c.242A>G	p.Glu81Gly	LP	InterVar Likely Pathogenic	17	0	-
chr3_169381438_TGAGG_T	c.120_123del	p.Leu41IlefsTer51	LP	InterVar Likely Pathogenic	0	1	-
chr3_169663338_G_A	c.35C>T	p.Thr12Ile	LP	InterVar Likely Pathogenic	0	2	-

RUNX1 P/LP	ENST00000300305.7				Number of heterozygotes / Found in gnomAD		
**Unique ID**	**HGVS c.**	**HGVS p.**	**Pathogenicity**	**Reason for Pathogenicity Call**	**DiscovEHR**	**UKBB**	**gnomAD (Y/-)**
chr21_34799295_GCTTA_G	c.967+2_967+5del	-	P	ClinVar Pathogenic	1	0	-
chr21_34799300_C_T	c.967+1G>A	-	LP	InterVar Likely Pathogenic	0	1	-
chr21_34799360_GA_G	c.907del	p.Ser303HisfsTer8	LP	InterVar Likely Pathogenic	1	0	-
chr21_34821671_A_T	c.805+12739T>A	-	LP	InterVar Likely Pathogenic	1	0	-
chr21_34859477_G_A	c.610C>T	p.Arg204Ter	P	ClinVar Pathogenic	1	0	-
chr21_34859485_C_T	c.602G>A	p.Arg201Gln	P	ClinVar Pathogenic	0	1	-
chr21_34859556_GA_G	c.530delT	p.Ile177fs	LP	InterVar Likely Pathogenic	0	1	-
chr21_34859579_C_T	c.509-1G>A	-	P	InterVar Pathogenic	0	1	-
chr21_34880556_C_T	c.508+1G>A	-	LP	InterVar Likely Pathogenic	2	0	-
chr21_34880568_C_T	c.497G>A	p.Arg166Gln	P	ClinVar Pathogenic	2	1	-
chr21_34880569_G_A	c.496C>T	p.Arg166Ter	P	InterVar Pathogenic	0	1	Y
chr21_34880581_T_C	c.484A>G	p.Arg162Gly	LP	ClinVar Likely Pathogenic	1	0	-
chr21_34886874_C_T	c.320G>A	p.Arg107His	LP	InterVar Likely Pathogenic	1	1	-
chr21_34886901_AG_A	c.292delC	p.Leu98fs	P	ClinVar Pathogenic	0	1	-
chr21_34886928_A_ATGGT	c.265_266insACCA	p.Leu89HisfsTer50	LP	InterVar Likely Pathogenic	1	0	-

SRP72 P/LP	ENST00000342756.9				Number of heterozygotes / Found in gnomAD		
Unique ID	HGVS c.	HGVS p.	Pathogenicity	Reason for Pathogenicity Call	DiscovEHR	UKBB	gnomAD (Y/-)
chr4_56467664_C_G	c.29C>G	p.Ser10Ter	P	InterVar Pathogenic	1	1	-
chr4_56467741_A_T	c.106A>T	p.Lys36Ter	P	InterVar Pathogenic	3	2	Y
chr4_56469681_TG_T	c.139delG	p.Ala47fs	LP	InterVar Likely Pathogenic	0	5	-
chr4_56469687_G_GC	c.145dupC	p.His49fs	LP	InterVar Likely Pathogenic	0	1	-
chr4_56469775_T_G	c.230+2T>G	-	P	InterVar Pathogenic	0	1	-
chr4_56471722_ACT_A	c.241_242del	p.Ser81LeufsTer2	LP	InterVar Likely Pathogenic	0	1	-
chr4_56471722_ACTCT_A	c.239_242del	p.Leu80ProfsTer11	LP	InterVar Likely Pathogenic	1	7	Y
chr4_56471776_A_AT	c.288dup	p.Ala97CysfsTer30	LP	InterVar Likely Pathogenic	1	0	-
chr4_56471820_CT_C	c.332del	p.Leu111ArgfsTer19	LP	InterVar Likely Pathogenic	1	0	-
chr4_56474055_T_G	c.356T>G	p.Leu119Ter	P	InterVar Pathogenic	0	1	Y
chr4_56474108_C_T	c.409C>T	p.Arg137Ter	P	InterVar Pathogenic	1	4	Y
chr4_56474150_C_CT	c.454dup	p.Ser152PhefsTer26	LP	InterVar Likely Pathogenic	0	1	-
chr4_56474171_C_T	c.472C>T	p.Gln158Ter	P	InterVar Pathogenic	0	1	-
chr4_56474183_G_T	c.484G>T	p.Glu162Ter	P	InterVar Pathogenic	0	1	-
chr4_56474280_G_T	c.499G>T	p.Glu167Ter	P	InterVar Pathogenic	0	1	-
chr4_56474342_AGG_A	c.562_563delGG	p.Gly188fs	LP	InterVar Likely Pathogenic	0	2	-
chr4_56476694_G_T	c.634G>T	p.Glu212Ter	P	InterVar Pathogenic	0	1	-
chr4_56476701_CTG_C	c.642+1_642+2delGT	-	LP	InterVar Likely Pathogenic	0	1	-
chr4_56478427_C_T	c.691C>T	p.Gln231Ter	P	InterVar Pathogenic	3	1	-
chr4_56478483_CAATCAAAT_C	c.751_758delCAAATAAT	p.Gln251fs	LP	InterVar Likely Pathogenic	0	1	-
chr4_56483142_C_T	c.829C>T	p.Gln277Ter	P	InterVar Pathogenic	0	1	-
chr4_56483157_TC_T	c.846delC	p.Lys283fs	LP	InterVar Likely Pathogenic	0	1	-
chr4_56483234_A_AT	c.924dup	p.Asn309Ter	LP	InterVar Likely Pathogenic	0	2	-
chr4_56483269_AGG_A	c.957_957+1delGG	p.Ala320fs	LP	InterVar Likely Pathogenic	0	1	-
chr4_56484734_AGGC_A	c.958-1_959delGGC	p.Ala320fs	LP	InterVar Likely Pathogenic	0	7	-
chr4_56484784_G_T	c.1006G>T	p.Glu336Ter	P	InterVar Pathogenic	0	1	-
chr4_56484792_C_CT	c.1016dup	p.Leu339PhefsTer13	LP	InterVar Likely Pathogenic	0	3	Y
chr4_56484794_T_TA	c.1017dup	p.Pro340ThrfsTer12	LP	InterVar Likely Pathogenic	2	0	-
chr4_56484797_C_CTG	c.1023_1024dup	p.Leu342CysfsTer2	LP	InterVar Likely Pathogenic	1	0	-
chr4_56484817_C_T	c.1039C>T	p.Gln347Ter	P	InterVar Pathogenic	0	1	-
chr4_56484862_C_T	c.1084C>T	p.Gln362Ter	P	InterVar Pathogenic	0	1	-
chr4_56486398_G_T	c.1159+1G>T	-	P	InterVar Pathogenic	0	1	-
chr4_56487994_T_A	c.1205T>A	p.Leu402Ter	P	InterVar Pathogenic	0	3	-
chr4_56488001_TAA_T	c.1214_1215del	p.Lys405ThrfsTer12	LP	InterVar Likely Pathogenic	3	0	Y
chr4_56489395_C_CA	c.1233dup	p.Leu412IlefsTer6	LP	InterVar Likely Pathogenic	1	0	-
chr4_56489398_T_G	c.1235T>G	p.Leu412Ter	P	InterVar Pathogenic	0	1	-
chr4_56489468_G_A	c.1305G>A	p.Trp435Ter	P	InterVar Pathogenic	0	1	-
chr4_56490411_A_AT	c.1401dupT	p.Ser468fs	LP	InterVar Likely Pathogenic	0	1	-
chr4_56490646_G_A	c.1502+1G>A	-	P	InterVar Pathogenic	0	31	Y
chr4_56491432_CTTAGTA_CTAGT	c.1506delCTTAGTAinsCTAGT	p.Ser503fs	LP	InterVar Likely Pathogenic	0	3	-
chr4_56491454_CAGAT_C	c.1529_1532del	p.Asp510ValfsTer4	LP	InterVar Likely Pathogenic	2	0	-
chr4_56491513_T_TA	c.1586dupA	p.Tyr529fs	LP	InterVar Likely Pathogenic	0	2	-
chr4_56500565_GT_G	c.1710del	p.Thr571ProfsTer49	LP	InterVar Likely Pathogenic	0	2	-

***ANKRD26* dVUS**

Unique ID	HGVS c.	HGVS p.	Pathogenicity	Reason for Pathogenicity Call	DiscovEHR (# of heterozygotes)	Found in gnomAD (Y/-)
chr10_27005622_CAT_C	c.5099_5100del	p.Tyr1700CysfsTer31	dVUS	≥3 of 5 in silico tools	2	-
chr10_27005685_GA_G	c.5037del	p.Leu1680Ter	dVUS	≥3 of 5 in silico tools	3	-
chr10_27006917_CTTCT_C	c.4995_4998del	p.Glu1666LeufsTer10	dVUS	≥3 of 5 in silico tools	1	-
chr10_27012881_C_A	c.4953+1G>T	-	dVUS	≥3 of 5 in silico tools	1	-
chr10_27012882_C_T	c.4953G>A	p.Lys1651=	dVUS	≥3 of 5 in silico tools	1	-
chr10_27013046_A_AAG	c.4787_4788dup	p.Phe1598CysfsTer23	LP	InterVar Likely Pathogenic	2	-
chr10_27013120_T_C	c.4725-10A>G	-	dVUS	≥3 of 5 in silico tools	5	-
chr10_27014489_C_G	c.4724+5G>C	-	dVUS	≥3 of 5 in silico tools	1	-
chr10_27014492_A_T	c.4724+2T>A	-	dVUS	≥3 of 5 in silico tools	1	-
chr10_27014542_T_TA	c.4675dup	p.Tyr1559LeufsTer8	LP	InterVar Likely Pathogenic	7	-
chr10_27014561_C_A	c.4657G>T	p.Glu1553Ter	P	InterVar Pathogenic	4	-
chr10_27014633_T_C	c.4585A>G	p.Arg1529Gly	dVUS	≥3 of 5 in silico tools	1	-
chr10_27017502_C_A	c.4506G>T	p.Gln1502His	dVUS	≥3 of 5 in silico tools	3	-
chr10_27017565_CT_C	c.4442del	p.Glu1481GlyfsTer10	LP	InterVar Likely Pathogenic	6	-
chr10_27017626_A_G	c.4382T>C	p.Leu1461Pro	dVUS	≥3 of 5 in silico tools	13	-
chr10_27017665_TG_T	c.4342del	p.Gln1448ArgfsTer11	LP	InterVar Likely Pathogenic	2	-
chr10_27017691_TG_T	c.4316del	p.Thr1439LysfsTer20	LP	InterVar Likely Pathogenic	2	-
chr10_27017755_G_A	c.4253C>T	p.Ser1418Leu	dVUS	≥3 of 5 in silico tools	1	-
chr10_27017757_AC_A	c.4250del	p.Gly1417ValfsTer21	LP	InterVar Likely Pathogenic	1	-
chr10_27017794_T_G	c.4216-2A>C	-	dVUS	≥3 of 5 in silico tools	13	Y
chr10_27022652_A_C	c.4121T>G	p.Leu1374Ter	P	InterVar Pathogenic	1	-
chr10_27024459_CT_C	c.4072del	p.Arg1358GlufsTer3	LP	InterVar Likely Pathogenic	1	-
chr10_27024471_AC_A	c.4060del	p.Val1354LeufsTer3	LP	InterVar Likely Pathogenic	4	-
chr10_27024523_C_A	c.4009G>T	p.Glu1337Ter	LP	InterVar Likely Pathogenic	3	Y
chr10_27024533_CT_C	c.3998del	p.Lys1333ArgfsTer6	LP	InterVar Likely Pathogenic	1	-
chr10_27024544_CCTTTT_C	c.3983_3987del	p.Glu1328GlyfsTer16	LP	InterVar Likely Pathogenic	1	-
chr10_27024550_C_A	c.3982G>T	p.Glu1328Ter	LP	InterVar Likely Pathogenic	1	-
chr10_27028901_T_G	c.3923A>C	p.Gln1308Pro	dVUS	≥3 of 5 in silico tools	1	-
chr10_27029338_G_A	c.3826C>T	p.Arg1276Ter	P	InterVar Pathogenic	2	Y
chr10_27033263_G_A	c.3769C>T	p.Gln1257Ter	P	InterVar Pathogenic	1	-
chr10_27033275_C_T	c.3757G>A	p.Glu1253Lys	dVUS	≥3 of 5 in silico tools	2	-
chr10_27033287_G_A	c.3745C>T	p.Arg1249Cys	dVUS	≥3 of 5 in silico tools	70	Y
chr10_27033329_GT_G	c.3699del	p.Lys1233AsnfsTer19	LP	InterVar Likely Pathogenic	1	-
chr10_27033346_G_A	c.3686C>T	p.Ala1229Val	dVUS	≥3 of 5 in silico tools	1	-
chr10_27034863_A_T	c.3587T>A	p.Leu1196Ter	P	InterVar Pathogenic	1	-
chr10_27035010_C_G	c.3440G>C	p.Arg1147Pro	dVUS	≥3 of 5 in silico tools	2	-
chr10_27035011_G_A	c.3439C>T	p.Arg1147Ter	dVUS	≥3 of 5 in silico tools	2	Y
chr10_27035013_A_G	c.3437T>C	p.Leu1146Pro	dVUS	≥3 of 5 in silico tools	1	-
chr10_27035058_GA_G	c.3391del	p.Ser1131LeufsTer2	LP	InterVar Likely Pathogenic	1	Y
chr10_27035164_G_GT	c.3285dup	p.Arg1096ThrfsTer23	LP	InterVar Likely Pathogenic	1	-

chr10_27035174_CA_C	c.3275del	p.Leu1092TrpfsTer3	LP	InterVar Likely Pathogenic	1	-
chr10_27035180_C_CTT	c.3268_3269dup	p.Thr1091ArgfsTer5	dVUS	≥3 of 5 in silico tools	12	-
0_27035261_A_ATTCTCAACT[illegible]	[illegible]3189insAAATAGT[illegible]	p.Gln1064AsnfsTer3	LP	InterVar Likely Pathogenic	1	-
chr10_27035265_A_T	c.3185T>A	p.Leu1062His	dVUS	≥3 of 5 in silico tools	1	-
chr10_27035467_C_A	c.2983G>T	p.Glu995Ter	P	InterVar Pathogenic	1	-
chr10_27035620_C_A	c.2830G>T	p.Glu944Ter	dVUS	≥3 of 5 in silico tools	1	Y
chr10_27035624_ACATTT_A	c.2821_2825del	p.Lys941PhefsTer2	LP	InterVar Likely Pathogenic	4	-
chr10_27037284_G_A	c.2599C>T	p.Arg867Ter	LP	InterVar Likely Pathogenic	1	-
chr10_27037299_G_A	c.2584C>T	p.Gln862Ter	P	InterVar Pathogenic	1	-
chr10_27037311_G_A	c.2572C>T	p.Arg858Ter	dVUS	≥3 of 5 in silico tools	2	Y
chr10_27037936_G_A	c.2494C>T	p.Gln832Ter	dVUS	≥3 of 5 in silico tools	5	Y
chr10_27038020_TTCTTC_T	c.2405_2409del	p.Arg802LysfsTer3	dVUS	≥3 of 5 in silico tools	1	-
chr10_27038038_C_A	c.2392G>T	p.Glu798Ter	P	InterVar Pathogenic	4	Y
chr10_27038057_G_T	c.2376-3C>A	-	dVUS	≥3 of 5 in silico tools	1	-
chr10_27039984_G_A	c.2356C>T	p.Arg786Ter	dVUS	≥3 of 5 in silico tools	3	Y
chr10_27040007_TCTAA_T	c.2329_2332del	p.Leu777SerfsTer14	dVUS	≥3 of 5 in silico tools	8	-
chr10_27040071_C_A	c.2269G>T	p.Glu757Ter	LP	InterVar Likely Pathogenic	1	-
chr10_27040080_T_A	c.2260A>T	p.Lys754Ter	dVUS	≥3 of 5 in silico tools	6	Y
chr10_27040101_C_A	c.2239G>T	p.Glu747Ter	LP	InterVar Likely Pathogenic	1	-
chr10_27040108_A_AT	c.2231dup	p.Asn744LysfsTer4	dVUS	≥3 of 5 in silico tools	1	-
chr10_27043423_T_TA	c.2161+2dup	-	dVUS	≥3 of 5 in silico tools	1	-
chr10_27043459_AC_A	c.2127del	p.Met709IlefsTer18	dVUS	≥3 of 5 in silico tools	21	-
chr10_27043568_C_T	c.2020-1G>A	-	dVUS	≥3 of 5 in silico tools	2	-
chr10_27044192_T_A	c.1986-2A>T	-	dVUS	≥3 of 5 in silico tools	3	-
chr10_27046526_T_C	c.1815-3A>G	-	dVUS	≥3 of 5 in silico tools	9	-
chr10_27046536_C_T	c.1815-13G>A	-	dVUS	≥3 of 5 in silico tools	3	-
chr10_27048799_AC_A	c.1814+1del	-	dVUS	≥3 of 5 in silico tools	1	-
chr10_27048887_AT_A	c.1727del	p.Asp576ValfsTer6	dVUS	≥3 of 5 in silico tools	10	-
chr10_27048954_CTG_C	c.1659_1660del	p.His553GlnfsTer3	LP	InterVar Likely Pathogenic	4	-
chr10_27048980_C_T	c.1636-1G>A	-	dVUS	≥3 of 5 in silico tools	1	-
chr10_27053323_T_TG	c.1631dup	p.Gln545ThrfsTer3	LP	InterVar Likely Pathogenic	3	-
chr10_27053346_C_A	c.1609G>T	p.Glu537Ter	LP	InterVar Likely Pathogenic	1	-
chr10_27060395_GA_G	c.1513del	p.Ser505LeufsTer9	dVUS	≥3 of 5 in silico tools	2	-
chr10_27060397_ATCTT_A	c.1508_1511del	p.Lys503IlefsTer10	LP	InterVar Likely Pathogenic	3	-
chr10_27061242_T_A	c.1364A>T	p.Asp455Val	dVUS	≥3 of 5 in silico tools	1	-
chr10_27064054_AC_A	c.1443delG	p.Lys481fs	LP	InterVar Likely Pathogenic	1	-
chr10_27064060_G_A	c.1291C>T	p.Gln431Ter	dVUS	≥3 of 5 in silico tools	1	-
chr10_27066484_T_C	c.1269+3A>G	-	dVUS	≥3 of 5 in silico tools	2	-
chr10_27066512_T_C	c.1244A>G	p.Asp415Gly	dVUS	≥3 of 5 in silico tools	3	-
chr10_27067214_G_A	c.1150C>T	p.Gln384Ter	P	InterVar Pathogenic	3	-
chr10_27067253_C_CT	c.1110dup	p.Glu371ArgfsTer5	dVUS	≥3 of 5 in silico tools	1	-
chr10_27077379_T_TA	c.1035_1036insT	p.Lys346Ter	dVUS	≥3 of 5 in silico tools	35	Y

chr10_27077461_AAC_A	c.952_953del	p.Val318CysfsTer3	LP	InterVar Likely Pathogenic	1	Y
chr10_27077628_C_T	c.874+5G>A	-	dVUS	≥3 of 5 in silico tools	2	-
chr10_27077695_T_C	c.814-2A>G	-	dVUS	≥3 of 5 in silico tools	1	-
chr10_27079088_C_T	c.813+1G>A	-	dVUS	≥3 of 5 in silico tools	1	-
chr10_27079129_C_T	c.773G>A	p.Trp258Ter	P	InterVar Pathogenic	3	-
chr10_27082804_T_A	c.739A>T	p.Arg247Trp	dVUS	≥3 of 5 in silico tools	32	Y
chr10_27092488_C_A	c.556G>T	p.Ala186Ser	dVUS	≥3 of 5 in silico tools	4	Y
chr10_27092488_C_T	c.556G>A	p.Ala186Thr	dVUS	≥3 of 5 in silico tools	1	Y
chr10_27092500_G_A	c.544C>T	p.Pro182Ser	dVUS	≥3 of 5 in silico tools	2	-
chr10_27092500_G_T	c.544C>A	p.Pro182Thr	dVUS	≥3 of 5 in silico tools	1	-
chr10_27092515_G_C	c.532-3C>G	-	dVUS	≥3 of 5 in silico tools	2	-
chr10_27093384_G_A	c.496C>T	p.Leu166Phe	dVUS	≥3 of 5 in silico tools	1	-
chr10_27093437_G_A	c.443C>T	p.Thr148Ile	dVUS	≥3 of 5 in silico tools	1	-
chr10_27093452_T_C	c.428A>G	p.Asp143Gly	dVUS	≥3 of 5 in silico tools	1	-
chr10_27093470_G_C	c.410C>G	p.Ala137Gly	dVUS	≥3 of 5 in silico tools	11	-
chr10_27093685_C_A	c.357G>T	p.Lys119Asn	dVUS	≥3 of 5 in silico tools	1	-
chr10_27093782_G_A	c.260C>T	p.Ala87Val	dVUS	≥3 of 5 in silico tools	1	-
chr10_27093792_G_C	c.250C>G	p.Leu84Val	dVUS	≥3 of 5 in silico tools	4	-
chr10_27093797_G_A	c.245C>T	p.Thr82Met	dVUS	≥3 of 5 in silico tools	8	Y
chr10_27100137_G_A	c.190C>T	p.Gln64Ter	LP	InterVar Likely Pathogenic	1	-
chr10_27100142_A_G	c.185T>C	p.Val62Ala	dVUS	≥3 of 5 in silico tools	2	-
chr10_27100194_G_A	c.133C>T	p.Arg45Ter	LP	InterVar Likely Pathogenic	1	-
chr10_27100220_G_T	c.107C>A	p.Ser36Ter	dVUS	≥3 of 5 in silico tools	1	-
chr10_27100222_G_C	c.105C>G	p.Tyr35Ter	dVUS	≥3 of 5 in silico tools	42	Y
chr10_27100232_TC_T	c.94del	p.Glu32ArgfsTer42	LP	InterVar Likely Pathogenic	6	-
chr10_27100269_G_A	c.58C>T	p.Gln20Ter	LP	InterVar Likely Pathogenic	1	-
chr10_27100290_AG_A	c.36del	p.Leu13TrpfsTer61	LP	InterVar Likely Pathogenic	1	-

***CEBPA* dVUS**

Unique ID	HGVS c.	HGVS p.	Pathogenicity	Reason for Pathogenicity Call	DiscovEHR (# of heterozygotes)	Found in gnomAD (Y/-)
chr19_33301345_C_T	c.1070G>A	p.Cys357Tyr	dVUS	≥3 of 5 in silico tools	2	-
chr19_33301346_A_G	c.1069T>C	p.Cys357Arg	dVUS	≥3 of 5 in silico tools	1	-
chr19_33301456_T_A	c.959A>T	p.Asp320Val	dVUS	≥3 of 5 in silico tools	2	-
chr19_33301498_C_T	c.917G>A	p.Arg306His	dVUS	≥3 of 5 in silico tools	1	-
chr19_33301508_C_T	c.907G>A	p.Ala303Thr	dVUS	≥3 of 5 in silico tools	1	-
chr19_33301523_T_G	c.892A>C	p.Lys298Gln	dVUS	≥3 of 5 in silico tools	4	Y
chr19_33301525_C_G	c.890G>C	p.Arg297Pro	dVUS	≥3 of 5 in silico tools	1	-
chr19_33301543_C_T	c.872G>A	p.Arg291His	dVUS	≥3 of 5 in silico tools	1	-
chr19_33302220_G_C	c.195C>G	p.Ser65Arg	dVUS	≥3 of 5 in silico tools	1	-
chr19_33302240_C_T	c.175G>A	p.Glu59Lys	dVUS	≥3 of 5 in silico tools	1	-

***DDX41* dVUS**

Unique ID	HGVS c.	HGVS p.	Pathogenicity	Reason for Pathogenicity Call	DiscovEHR (# of heterozygotes)	Found in gnomAD (Y/-)
chr5_177511861_G_C	c.1799C>G	p.Ala600Gly	dVUS	≥3 of 5 in silico tools	1	-
chr5_177511880_C_G	c.1780G>C	p.Asp594His	dVUS	≥3 of 5 in silico tools	2	Y
chr5_177512085_TCTGCAGTCACCTCC_T	c.1729_1732+10del	-	dVUS	≥3 of 5 in silico tools	8	-
chr5_177512149_G_A	c.1679C>T	p.Pro560Leu	dVUS	≥3 of 5 in silico tools	1	-
chr5_177512149_G_C	c.1679C>G	p.Pro560Arg	dVUS	≥3 of 5 in silico tools	1	-
chr5_177512165_C_T	c.1663G>A	p.Ala555Thr	dVUS	≥3 of 5 in silico tools	2	-
chr5_177512197_A_G	c.1631T>C	p.Val544Ala	dVUS	≥3 of 5 in silico tools	1	-
chr5_177512198_C_T	c.1630G>A	p.Val544Met	dVUS	≥3 of 5 in silico tools	10	-
chr5_177512343_T_C	c.1600A>G	p.Thr534Ala	dVUS	≥3 of 5 in silico tools	7	-
chr5_177512349_C_T	c.1594G>A	p.Ala532Thr	dVUS	≥3 of 5 in silico tools	3	Y
chr5_177512355_CTG_C	c.1586_1587del	p.Thr529ArgfsTer12	dVUS	≥3 of 5 in silico tools	1	-
chr5_177512358_T_C	c.1585A>G	p.Thr529Ala	dVUS	≥3 of 5 in silico tools	1	-
chr5_177512498_T_C	c.1547A>G	p.Tyr516Cys	dVUS	≥3 of 5 in silico tools	4	Y
chr5_177512507_A_G	c.1538T>C	p.Ile513Thr	dVUS	≥3 of 5 in silico tools	2	Y
chr5_177512517_G_A	c.1528C>T	p.Pro510Ser	dVUS	≥3 of 5 in silico tools	7	Y
chr5_177512528_T_C	c.1517A>G	p.Asn506Ser	dVUS	≥3 of 5 in silico tools	3	-
chr5_177512535_C_T	c.1510G>A	p.Val504Ile	dVUS	≥3 of 5 in silico tools	4	Y
chr5_177512597_T_C	c.1448A>G	p.Lys483Arg	dVUS	≥3 of 5 in silico tools	1	-
chr5_177512610_G_A	c.1435C>T	p.Arg479Trp	dVUS	≥3 of 5 in silico tools	1	Y
chr5_177512644_G_C	c.1401C>G	p.Asp467Glu	dVUS	≥3 of 5 in silico tools	2	-
chr5_177512810_C_A	c.1369G>T	p.Val457Phe	dVUS	≥3 of 5 in silico tools	5	-
chr5_177512858_T_C	c.1321A>G	p.Lys441Glu	dVUS	≥3 of 5 in silico tools	1	-
chr5_177513012_G_A	c.1301C>T	p.Pro434Leu	dVUS	≥3 of 5 in silico tools	3	Y
chr5_177513015_G_A	c.1298C>T	p.Pro433Leu	dVUS	≥3 of 5 in silico tools	1	-
chr5_177513027_T_C	c.1286A>G	p.Gln429Arg	dVUS	≥3 of 5 in silico tools	1	-
chr5_177513030_A_G	c.1283T>C	p.Leu428Pro	dVUS	≥3 of 5 in silico tools	1	-
chr5_177513031_G_T	c.1282C>A	p.Leu428Met	dVUS	≥3 of 5 in silico tools	2	-
chr5_177513075_T_C	c.1238A>G	p.Glu413Gly	dVUS	≥3 of 5 in silico tools	1	Y
chr5_177513078_A_C	c.1235T>G	p.Val412Gly	dVUS	≥3 of 5 in silico tools	2	-
chr5_177513396_A_G	c.1187T>C	p.Ile396Thr	dVUS	≥3 of 5 in silico tools	15	Y
chr5_177513442_T_A	c.1141A>T	p.Lys381Ter	dVUS	≥3 of 5 in silico tools	1	-
chr5_177513478_G_A	c.1105C>T	p.Arg369Ter	dVUS	≥3 of 5 in silico tools	6	Y
chr5_177513478_G_C	c.1105C>G	p.Arg369Gly	dVUS	≥3 of 5 in silico tools	5	-
chr5_177513704_G_A	c.1079C>T	p.Thr360Ile	dVUS	≥3 of 5 in silico tools	2	Y
chr5_177513725_C_T	c.1058G>A	p.Gly353Asp	dVUS	≥3 of 5 in silico tools	1	-
chr5_177513732_C_T	c.1051G>A	p.Asp351Asn	dVUS	≥3 of 5 in silico tools	2	Y
chr5_177513737_A_T	c.1046T>A	p.Met349Lys	dVUS	≥3 of 5 in silico tools	7	Y
chr5_177513740_C_T	c.1043G>A	p.Arg348His	dVUS	≥3 of 5 in silico tools	1	-
chr5_177513741_G_A	c.1042C>T	p.Arg348Cys	dVUS	≥3 of 5 in silico tools	1	-
chr5_177513750_C_T	c.1033G>A	p.Glu345Lys	dVUS	≥3 of 5 in silico tools	4	Y
chr5_177513753_C_A	c.1030G>T	p.Asp344Tyr	dVUS	≥3 of 5 in silico tools	3	-
chr5_177513765_A_T	c.1018T>A	p.Tyr340Asn	dVUS	≥3 of 5 in silico tools	1	-
chr5_177513815_C_T	c.968G>A	p.Arg323His	dVUS	≥3 of 5 in silico tools	1	-
chr5_177513816_G_A	c.967C>T	p.Arg323Cys	dVUS	≥3 of 5 in silico tools	1	Y

chr5_177513824_G_A	c.959C>T	p.Thr320Ile	dVUS	≥3 of 5 in silico tools	2	-
chr5_177513825_T_A	c.958A>T	p.Thr320Ser	dVUS	≥3 of 5 in silico tools	1	-
chr5_177513835_CAT_C	c.946_947del	p.Met316AspfsTer31	dVUS	≥3 of 5 in silico tools	10	Y
chr5_177513845_C_T	c.938G>A	p.Gly313Asp	dVUS	≥3 of 5 in silico tools	1	-
chr5_177513846_C_G	c.937G>C	p.Gly313Arg	dVUS	≥3 of 5 in silico tools	2	-
chr5_177513846_C_T	c.937G>A	p.Gly313Ser	dVUS	≥3 of 5 in silico tools	4	Y
chr5_177514700_C_A	c.935+1G>T	-	dVUS	≥3 of 5 in silico tools	1	-
chr5_177514758_C_T	c.878G>A	p.Arg293His	dVUS	≥3 of 5 in silico tools	2	-
chr5_177514759_G_A	c.877C>T	p.Arg293Cys	dVUS	≥3 of 5 in silico tools	1	Y
chr5_177514759_G_C	c.877C>G	p.Arg293Gly	dVUS	≥3 of 5 in silico tools	2	-
chr5_177514797_T_C	c.839A>G	p.Tyr280Cys	dVUS	≥3 of 5 in silico tools	5	-
chr5_177514827_G_A	c.809C>T	p.Ala270Val	dVUS	≥3 of 5 in silico tools	2	Y
chr5_177514828_C_T	c.808G>A	p.Ala270Thr	dVUS	≥3 of 5 in silico tools	2	-
chr5_177514837_G_A	c.799C>T	p.Arg267Trp	dVUS	≥3 of 5 in silico tools	8	Y
chr5_177514945_C_G	c.769G>C	p.Gly257Arg	dVUS	≥3 of 5 in silico tools	4	-
chr5_177514980_T_A	c.734A>T	p.Glu245Val	dVUS	≥3 of 5 in silico tools	2	-
chr5_177515037_A_G	c.677T>C	p.Phe226Ser	dVUS	≥3 of 5 in silico tools	2	-
chr5_177515041_C_T	c.673G>A	p.Ala225Thr	dVUS	≥3 of 5 in silico tools	9	-
chr5_177515059_G_A	c.655C>T	p.Arg219Cys	dVUS	≥3 of 5 in silico tools	35	Y
chr5_177515061_C_T	c.653G>A	p.Gly218Asp	dVUS	≥3 of 5 in silico tools	2	Y
chr5_177515207_T_C	c.623A>G	p.Gln208Arg	dVUS	≥3 of 5 in silico tools	13	-
chr5_177515228_A_G	c.602T>C	p.Ile201Thr	dVUS	≥3 of 5 in silico tools	1	-
chr5_177515232_C_T	c.598G>A	p.Gly200Ser	dVUS	≥3 of 5 in silico tools	1	-
chr5_177515781_G_A	c.475C>T	p.Arg159Ter	dVUS	≥3 of 5 in silico tools	1	-
chr5_177515928_C_T	c.434+1G>A	-	dVUS	≥3 of 5 in silico tools	2	-
chr5_177516173_C_T	c.319G>A	p.Glu107Lys	dVUS	≥3 of 5 in silico tools	1	-
chr5_177516286_A_T	c.298+2T>A	-	dVUS	≥3 of 5 in silico tools	1	-
chr5_177516318_G_A	c.268C>T	p.Gln90Ter	dVUS	≥3 of 5 in silico tools	3	Y
chr5_177516724_C_G	c.138+1G>C	-	dVUS	≥3 of 5 in silico tools	1	-
chr5_177516750_G_A	c.113C>T	p.Pro38Leu	dVUS	≥3 of 5 in silico tools	3	Y
chr5_177516750_G_C	c.113C>G	p.Pro38Arg	dVUS	≥3 of 5 in silico tools	4	Y
chr5_177516759_G_A	c.104C>T	p.Pro35Leu	dVUS	≥3 of 5 in silico tools	2	Y
chr5_177516836_C_G	c.28-1G>C	-	dVUS	≥3 of 5 in silico tools	1	-

ETV6 dVUS

Unique ID	HGVS c.	HGVS p.	Pathogenicity	Reason for Pathogenicity Call	DiscovEHR (# of heterozygotes)	Found in gnomAD (Y/-)
chr12_11752540_A_G	c.124A>G	p.Arg42Gly	dVUS	≥3 of 5 in silico tools	2	-
chr12_11752541_G_C	c.125G>C	p.Arg42Thr	dVUS	≥3 of 5 in silico tools	1	-
chr12_11839140_G_T	c.164G>T	p.Arg55Leu	dVUS	≥3 of 5 in silico tools	1	Y
chr12_11839147_G_T	c.171G>T	p.Gln57His	dVUS	≥3 of 5 in silico tools	1	-
chr12_11839176_C_A	c.200C>A	p.Ala67Asp	dVUS	≥3 of 5 in silico tools	1	-
chr12_11839266_T_C	c.290T>C	p.Leu97Pro	dVUS	≥3 of 5 in silico tools	1	-
chr12_11839283_C_G	c.307C>G	p.Arg103Gly	dVUS	≥3 of 5 in silico tools	1	-
chr12_11839283_C_T	c.307C>T	p.Arg103Cys	dVUS	≥3 of 5 in silico tools	1	-
chr12_11839284_G_A	c.308G>A	p.Arg103His	dVUS	≥3 of 5 in silico tools	2	Y
chr12_11839286_T_A	c.310T>A	p.Tyr104Asn	dVUS	≥3 of 5 in silico tools	1	-
chr12_11839290_G_A	c.314G>A	p.Arg105Gln	dVUS	≥3 of 5 in silico tools	1	-
chr12_11839293_C_T	c.317C>T	p.Ser106Phe	dVUS	≥3 of 5 in silico tools	2	-
chr12_11839304_G_A	c.328G>A	p.Gly110Ser	dVUS	≥3 of 5 in silico tools	1	-
chr12_11853447_C_T	c.349C>T	p.Leu117Phe	dVUS	≥3 of 5 in silico tools	12	Y
chr12_11853484_T_C	c.386T>C	p.Leu129Pro	dVUS	≥3 of 5 in silico tools	1	Y
chr12_11853484_T_G	c.386T>G	p.Leu129Arg	dVUS	≥3 of 5 in silico tools	1	-
chr12_11853499_T_G	c.401T>G	p.Phe134Cys	dVUS	≥3 of 5 in silico tools	1	-
chr12_11869645_C_G	c.685C>G	p.His229Asp	dVUS	≥3 of 5 in silico tools	2	-
chr12_11869658_A_G	c.698A>G	p.Tyr233Cys	dVUS	≥3 of 5 in silico tools	1	-
chr12_11869664_T_C	c.704T>C	p.Leu235Pro	dVUS	≥3 of 5 in silico tools	1	-
chr12_11869750_C_T	c.790C>T	p.Arg264Cys	dVUS	≥3 of 5 in silico tools	1	-
chr12_11869796_T_C	c.836T>C	p.Leu279Pro	dVUS	≥3 of 5 in silico tools	1	-
chr12_11869840_G_A	c.880G>A	p.Glu294Lys	dVUS	≥3 of 5 in silico tools	1	Y
chr12_11869885_C_T	c.925C>T	p.Arg309Trp	dVUS	≥3 of 5 in silico tools	4	Y
chr12_11884438_TCTGTAGA_T	c.1014_1020del	p.Arg339PhefsTer30	dVUS	≥3 of 5 in silico tools	1	-
chr12_11884508_T_C	c.1073T>C	p.Ile358Thr	dVUS	≥3 of 5 in silico tools	1	-
chr12_11884511_G_A	c.1076G>A	p.Arg359Gln	dVUS	≥3 of 5 in silico tools	1	-
chr12_11884537_T_G	c.1102T>G	p.Phe368Val	dVUS	≥3 of 5 in silico tools	1	-
chr12_11884558_G_A	c.1123G>A	p.Gly375Arg	dVUS	≥3 of 5 in silico tools	1	-
chr12_11884568_G_T	c.1133G>T	p.Arg378Leu	dVUS	≥3 of 5 in silico tools	1	-
chr12_11890985_G_A	c.1298G>A	p.Arg433His	dVUS	≥3 of 5 in silico tools	1	Y
chr12_11890993_C_A	c.1306C>A	p.His436Asn	dVUS	≥3 of 5 in silico tools	1	-
chr12_11891043_C_A	c.1356C>A	p.Cys452Ter	dVUS	≥3 of 5 in silico tools	2	Y

GATA2 dVUS

Unique ID	HGVS c.	HGVS p.	Pathogenicity	Reason for Pathogenicity Call	DiscovEHR (# of heterozygotes)	Found in gnomAD (Y/-)
chr3_128481030_C_T	c.1432G>A	p.Ala478Thr	dVUS	≥3 of 5 in silico tools	3	-
chr3_128481035_A_G	c.1427T>C	p.Val476Ala	dVUS	≥3 of 5 in silico tools	1	-
chr3_128481039_T_C	c.1423A>G	p.Met475Val	dVUS	≥3 of 5 in silico tools	2	-
chr3_128481047_G_T	c.1415C>A	p.Pro472Gln	dVUS	≥3 of 5 in silico tools	1	Y
chr3_128481071_C_A	c.1391G>T	p.Ser464Ile	dVUS	≥3 of 5 in silico tools	25	Y
chr3_128481083_T_A	c.1379A>T	p.His460Leu	dVUS	≥3 of 5 in silico tools	1	-
chr3_128481086_A_T	c.1376T>A	p.Ile459Asn	dVUS	≥3 of 5 in silico tools	2	Y
chr3_128481092_G_A	c.1370C>T	p.Thr457Met	dVUS	≥3 of 5 in silico tools	28	-
chr3_128481095_G_A	c.1367C>T	p.Pro456Leu	dVUS	≥3 of 5 in silico tools	1	Y
chr3_128481099_T_C	c.1363A>G	p.Thr455Ala	dVUS	≥3 of 5 in silico tools	2	-
chr3_128481102_G_A	c.1360C>T	p.Pro454Ser	dVUS	≥3 of 5 in silico tools	14	-
chr3_128481131_G_A	c.1331C>T	p.Pro444Leu	dVUS	≥3 of 5 in silico tools	10	Y
chr3_128481146_G_A	c.1316C>T	p.Pro439Leu	dVUS	≥3 of 5 in silico tools	2	-
chr3_128481162_C_G	c.1300G>C	p.Ala434Pro	dVUS	≥3 of 5 in silico tools	1	-
chr3_128481176_C_G	c.1286G>C	p.Ser429Thr	dVUS	≥3 of 5 in silico tools	7	Y
chr3_128481182_G_C	c.1280C>G	p.Pro427Arg	dVUS	≥3 of 5 in silico tools	2	-
chr3_128481239_T_C	c.1223A>G	p.Lys408Arg	dVUS	≥3 of 5 in silico tools	1	-
chr3_128481245_T_A	c.1217A>T	p.Lys406Met	dVUS	≥3 of 5 in silico tools	9	Y
chr3_128481247_C_A	c.1215G>T	p.Lys405Asn	dVUS	≥3 of 5 in silico tools	5	-
chr3_128481269_C_G	c.1193G>C	p.Arg398Pro	dVUS	≥3 of 5 in silico tools	1	-
chr3_128481314_T_C	c.1148A>G	p.Asn383Ser	dVUS	≥3 of 5 in silico tools	3	-
chr3_128481866_C_A	c.1096G>T	p.Gly366Trp	dVUS	≥3 of 5 in silico tools	1	-
chr3_128481867_G_T	c.1095C>A	p.Asn365Lys	dVUS	≥3 of 5 in silico tools	1	-
chr3_128481872_C_T	c.1090G>A	p.Ala364Thr	dVUS	≥3 of 5 in silico tools	2	Y
chr3_128481889_G_A	c.1073C>T	p.Thr358Ile	dVUS	≥3 of 5 in silico tools	1	-
chr3_128481926_C_T	c.1036G>A	p.Gly346Ser	dVUS	≥3 of 5 in silico tools	9	-
chr3_128481937_G_A	c.1025C>T	p.Ala342Val	dVUS	≥3 of 5 in silico tools	4	-
chr3_128481938_C_T	c.1024G>A	p.Ala342Thr	dVUS	≥3 of 5 in silico tools	15	Y
chr3_128483861_A_G	c.1016T>C	p.Leu339Pro	dVUS	≥3 of 5 in silico tools	1	-
chr3_128483876_T_C	c.1001A>G	p.Lys334Arg	dVUS	≥3 of 5 in silico tools	1	-
chr3_128483894_T_A	c.983A>T	p.Gln328Leu	dVUS	≥3 of 5 in silico tools	1	-
chr3_128483906_T_C	c.971A>G	p.Lys324Arg	dVUS	≥3 of 5 in silico tools	1	Y
chr3_128483909_T_C	c.968A>G	p.His323Arg	dVUS	≥3 of 5 in silico tools	5	-
chr3_128483958_G_A	c.919C>T	p.Arg307Trp	dVUS	≥3 of 5 in silico tools	1	-
chr3_128483978_G_A	c.899C>T	p.Ala300Val	dVUS	≥3 of 5 in silico tools	7	-

chr3_128485732_C_T	c.866G>A	p.Cys289Tyr	dVUS	≥3 of 5 in silico tools	1	-
chr3_128485759_G_C	c.839C>G	p.Pro280Arg	dVUS	≥3 of 5 in silico tools	1	-
chr3_128485766_A_T	c.832T>A	p.Phe278Ile	dVUS	≥3 of 5 in silico tools	4	Y
chr3_128485780_C_G	c.818G>C	p.Gly273Ala	dVUS	≥3 of 5 in silico tools	1	-
chr3_128485789_A_C	c.809T>G	p.Phe270Cys	dVUS	≥3 of 5 in silico tools	5	-
chr3_128485796_C_G	c.802G>C	p.Gly268Arg	dVUS	≥3 of 5 in silico tools	2	-
chr3_128485800_G_T	c.798C>A	p.His266Gln	dVUS	≥3 of 5 in silico tools	4	-
chr3_128485812_G_C	c.786C>G	p.Ser262Arg	dVUS	≥3 of 5 in silico tools	5	-
chr3_128485816_C_T	c.782G>A	p.Ser261Asn	dVUS	≥3 of 5 in silico tools	1	-
chr3_128485820_A_C	c.778T>G	p.Tyr260Asp	dVUS	≥3 of 5 in silico tools	1	-
chr3_128485822_T_C	c.776A>G	p.Asp259Gly	dVUS	≥3 of 5 in silico tools	2	-
chr3_128485837_G_C	c.761C>G	p.Pro254Arg	dVUS	≥3 of 5 in silico tools	2	-
chr3_128485843_T_C	c.755A>G	p.Tyr252Cys	dVUS	≥3 of 5 in silico tools	3	Y
chr3_128485850_G_A	c.748C>T	p.Pro250Ser	dVUS	≥3 of 5 in silico tools	2	Y
chr3_128485865_G_A	c.733C>T	p.Pro245Ser	dVUS	≥3 of 5 in silico tools	1	-
chr3_128485871_G_A	c.727C>T	p.His243Tyr	dVUS	≥3 of 5 in silico tools	1	-
chr3_128485891_A_G	c.707T>C	p.Met236Thr	dVUS	≥3 of 5 in silico tools	4	Y
chr3_128485892_T_C	c.706A>G	p.Met236Val	dVUS	≥3 of 5 in silico tools	64	Y
chr3_128485916_G_A	c.682C>T	p.Pro228Ser	dVUS	≥3 of 5 in silico tools	2	-
chr3_128485916_G_T	c.682C>A	p.Pro228Thr	dVUS	≥3 of 5 in silico tools	17	Y
chr3_128485962_C_A	c.636G>T	p.Lys212Asn	dVUS	≥3 of 5 in silico tools	1	-
chr3_128485979_C_T	c.619G>A	p.Asp207Asn	dVUS	≥3 of 5 in silico tools	1	-
chr3_128485987_C_T	c.611G>A	p.Arg204Gln	dVUS	≥3 of 5 in silico tools	1	Y
chr3_128486050_G_A	c.548C>T	p.Pro183Leu	dVUS	≥3 of 5 in silico tools	1	-
chr3_128486054_A_G	c.544T>C	p.Ser182Pro	dVUS	≥3 of 5 in silico tools	1	-
chr3_128486057_C_G	c.541G>C	p.Val181Leu	dVUS	≥3 of 5 in silico tools	1	-
chr3_128486062_T_C	c.536A>G	p.Lys179Arg	dVUS	≥3 of 5 in silico tools	1	Y
chr3_128486063_T_G	c.535A>C	p.Lys179Gln	dVUS	≥3 of 5 in silico tools	1	-
chr3_128486066_G_A	c.532C>T	p.Pro178Ser	dVUS	≥3 of 5 in silico tools	5	Y
chr3_128486071_G_T	c.527C>A	p.Thr176Lys	dVUS	≥3 of 5 in silico tools	1	-
chr3_128486090_G_A	c.508C>T	p.Leu170Phe	dVUS	≥3 of 5 in silico tools	2	-
chr3_128486101_G_C	c.497C>G	p.Ser166Cys	dVUS	≥3 of 5 in silico tools	4	-
chr3_128486104_T_C	c.494A>G	p.His165Arg	dVUS	≥3 of 5 in silico tools	3	-
chr3_128486119_G_T	c.479C>A	p.Thr160Asn	dVUS	≥3 of 5 in silico tools	1	-
chr3_128486162_C_A	c.436G>T	p.Gly146Cys	dVUS	≥3 of 5 in silico tools	1	-
chr3_128486182_G_A	c.416C>T	p.Ser139Phe	dVUS	≥3 of 5 in silico tools	2	-
chr3_128486185_A_G	c.413T>C	p.Leu138Pro	dVUS	≥3 of 5 in silico tools	22	Y

chr3_128486218_T_C	c.380A>G	p.His127Arg	dVUS	≥3 of 5 in silico tools	1	-
chr3_128486227_G_A	c.371C>T	p.Thr124Met	dVUS	≥3 of 5 in silico tools	3	Y
chr3_128486239_G_A	c.359C>T	p.Pro120Leu	dVUS	≥3 of 5 in silico tools	1	Y
chr3_128486240_G_A	c.358C>T	p.Pro120Ser	dVUS	≥3 of 5 in silico tools	11	Y
chr3_128486245_A_G	c.353T>C	p.Val118Ala	dVUS	≥3 of 5 in silico tools	1	-
chr3_128486258_T_C	c.340A>G	p.Asn114Asp	dVUS	≥3 of 5 in silico tools	6	-
chr3_128486260_T_C	c.338A>G	p.His113Arg	dVUS	≥3 of 5 in silico tools	1	-
chr3_128486269_G_A	c.329C>T	p.Ala110Val	dVUS	≥3 of 5 in silico tools	1	-
chr3_128486272_G_T	c.326C>A	p.Ala109Glu	dVUS	≥3 of 5 in silico tools	1	-
chr3_128486287_G_A	c.311C>T	p.Ala104Val	dVUS	≥3 of 5 in silico tools	1	-
chr3_128486293_T_A	c.305A>T	p.Lys102Ile	dVUS	≥3 of 5 in silico tools	1	-
chr3_128486299_C_A	c.299G>T	p.Gly100Val	dVUS	≥3 of 5 in silico tools	17	-
chr3_128486336_G_A	c.262C>T	p.His88Tyr	dVUS	≥3 of 5 in silico tools	16	-
chr3_128486346_C_T	c.252G>A	p.Met84Ile	dVUS	≥3 of 5 in silico tools	1	-
chr3_128486356_C_G	c.242G>C	p.Gly81Ala	dVUS	≥3 of 5 in silico tools	5	-
chr3_128486363_G_C	c.235C>G	p.Leu79Val	dVUS	≥3 of 5 in silico tools	1	Y
chr3_128486808_G_A	c.224C>T	p.Ala75Val	dVUS	≥3 of 5 in silico tools	1	-
chr3_128486809_C_T	c.223G>A	p.Ala75Thr	dVUS	≥3 of 5 in silico tools	3	-
chr3_128486827_G_A	c.205C>T	p.Arg69Cys	dVUS	≥3 of 5 in silico tools	1	-
chr3_128486839_G_A	c.193C>T	p.His65Tyr	dVUS	≥3 of 5 in silico tools	1	-
chr3_128486846_G_C	c.186C>G	p.Asn62Lys	dVUS	≥3 of 5 in silico tools	1	Y
chr3_128486846_G_T	c.186C>A	p.Asn62Lys	dVUS	≥3 of 5 in silico tools	1	-
chr3_128486850_G_A	c.182C>T	p.Ala61Val	dVUS	≥3 of 5 in silico tools	6	-
chr3_128486853_T_C	c.179A>G	p.Tyr60Cys	dVUS	≥3 of 5 in silico tools	1	-
chr3_128486856_T_C	c.176A>G	p.Tyr59Cys	dVUS	≥3 of 5 in silico tools	13	Y
chr3_128486857_A_T	c.175T>A	p.Tyr59Asn	dVUS	≥3 of 5 in silico tools	2	-
chr3_128486890_A_T	c.142T>A	p.Phe48Ile	dVUS	≥3 of 5 in silico tools	28	-
chr3_128486896_C_T	c.136G>A	p.Asp46Asn	dVUS	≥3 of 5 in silico tools	3	-
chr3_128486901_T_A	c.131A>T	p.Glu44Val	dVUS	≥3 of 5 in silico tools	1	-
chr3_128486908_G_C	c.124C>G	p.Pro42Ala	dVUS	≥3 of 5 in silico tools	1	-
chr3_128486939_G_C	c.93C>G	p.His31Gln	dVUS	≥3 of 5 in silico tools	1	-
chr3_128486940_T_C	c.92A>G	p.His31Arg	dVUS	≥3 of 5 in silico tools	1	-
chr3_128486941_G_A	c.91C>T	p.His31Tyr	dVUS	≥3 of 5 in silico tools	1	-
chr3_128486949_C_T	c.83G>A	p.Gly28Asp	dVUS	≥3 of 5 in silico tools	6	-
chr3_128486954_G_C	c.78C>G	p.His26Gln	dVUS	≥3 of 5 in silico tools	2	-
chr3_128486968_G_A	c.64C>T	p.Pro22Ser	dVUS	≥3 of 5 in silico tools	22	-
chr3_128486982_A_T	c.50T>A	p.Leu17Gln	dVUS	≥3 of 5 in silico tools	3	-

chr3_128486986_C_T	c.46G>A	p.Val16Met	dVUS	≥3 of 5 in silico tools	12	Y
chr3_128486992_G_A	c.40C>T	p.Pro14Ser	dVUS	≥3 of 5 in silico tools	26	Y
chr3_128487002_C_A	c.30G>T	p.Trp10Cys	dVUS	≥3 of 5 in silico tools	105	Y
chr3_128487006_C_T	c.26G>A	p.Arg9His	dVUS	≥3 of 5 in silico tools	1	-
chr3_128487018_G_A	c.14C>T	p.Pro5Leu	dVUS	≥3 of 5 in silico tools	1	-

MECOM dVUS

Unique ID	HGVS c.	HGVS p.	Pathogenicity	Reason for Pathogenicity Call	DiscovEHR (# of heterozygotes)	Found in gnomAD (Y/-)
chr3_169084926_A_G	c.3703T>C	p.Ser1235Pro	dVUS	≥3 of 5 in silico tools	1	-
chr3_169084931_A_G	c.3698T>C	p.Ile1233Thr	dVUS	≥3 of 5 in silico tools	1	-
chr3_169084955_C_G	c.3674G>C	p.Arg1225Thr	dVUS	≥3 of 5 in silico tools	28	-
chr3_169085021_A_G	c.3608T>C	p.Leu1203Pro	dVUS	≥3 of 5 in silico tools	5	Y
chr3_169085034_T_C	c.3595A>G	p.Met1199Val	dVUS	≥3 of 5 in silico tools	2	-
chr3_169085044_C_G	c.3586-1G>C	-	dVUS	≥3 of 5 in silico tools	1	-
chr3_169089010_G_A	c.3575C>T	p.Ser1192Phe	dVUS	≥3 of 5 in silico tools	7	-
chr3_169089136_A_G	c.3449T>C	p.Ile1150Thr	dVUS	≥3 of 5 in silico tools	1	-
chr3_169089142_T_A	c.3443A>T	p.Asp1148Val	dVUS	≥3 of 5 in silico tools	1	-
chr3_169089143_C_A	c.3442G>T	p.Asp1148Tyr	dVUS	≥3 of 5 in silico tools	1	-
chr3_169089998_A_C	c.3401+2T>G	-	dVUS	≥3 of 5 in silico tools	1	-
chr3_169093018_A_G	c.3104T>C	p.Ile1035Thr	dVUS	≥3 of 5 in silico tools	1	-
chr3_169093048_T_C	c.3074A>G	p.Asp1025Gly	dVUS	≥3 of 5 in silico tools	13	Y
chr3_169093079_A_G	c.3043T>C	p.Ser1015Pro	dVUS	≥3 of 5 in silico tools	1	-
chr3_169093103_C_A	c.3020-1G>T	-	dVUS	≥3 of 5 in silico tools	1	-
chr3_169093103_C_T	c.3020-1G>A	-	dVUS	≥3 of 5 in silico tools	2	-
chr3_169095096_T_A	c.2999A>T	p.His1000Leu	dVUS	≥3 of 5 in silico tools	1	-
chr3_169095139_A_G	c.2956T>C	p.Cys986Arg	dVUS	≥3 of 5 in silico tools	1	Y
chr3_169100921_C_G	c.2813G>C	p.Arg938Pro	dVUS	≥3 of 5 in silico tools	1	-
chr3_169102072_C_A	c.2759G>T	p.Arg920Leu	dVUS	≥3 of 5 in silico tools	1	-
chr3_169102072_C_G	c.2759G>C	p.Arg920Pro	dVUS	≥3 of 5 in silico tools	1	-
chr3_169102073_G_A	c.2758C>T	p.Arg920Cys	dVUS	≥3 of 5 in silico tools	14	Y
chr3_169102073_G_C	c.2758C>G	p.Arg920Gly	dVUS	≥3 of 5 in silico tools	1	-
chr3_169102216_A_G	c.2615T>C	p.Ile872Thr	dVUS	≥3 of 5 in silico tools	5	-
chr3_169102219_G_T	c.2612C>A	p.Ala871Asp	dVUS	≥3 of 5 in silico tools	1	-
chr3_169107925_C_A	c.2604+1G>T	-	dVUS	≥3 of 5 in silico tools	1	Y
chr3_169107925_C_T	c.2604+1G>A	-	dVUS	≥3 of 5 in silico tools	9	Y
chr3_169107965_T_C	c.2578-13A>G	-	dVUS	≥3 of 5 in silico tools	1	-
chr3_169112806_C_T	c.2558G>A	p.Gly853Glu	dVUS	≥3 of 5 in silico tools	3	Y
chr3_169115386_T_C	c.2486A>G	p.Tyr829Cys	dVUS	≥3 of 5 in silico tools	4	-
chr3_169115398_A_T	c.2474T>A	p.Met825Lys	dVUS	≥3 of 5 in silico tools	1	-
chr3_169115401_A_C	c.2471T>G	p.Phe824Cys	dVUS	≥3 of 5 in silico tools	4	Y
chr3_169115401_A_G	c.2471T>C	p.Phe824Ser	dVUS	≥3 of 5 in silico tools	1	Y
chr3_169115404_A_G	c.2468T>C	p.Phe823Ser	dVUS	≥3 of 5 in silico tools	59	Y
chr3_169115416_C_G	c.2456G>C	p.Arg819Thr	dVUS	≥3 of 5 in silico tools	1	-
chr3_169115644_T_C	c.2228A>G	p.Asp743Gly	dVUS	≥3 of 5 in silico tools	6	-
chr3_169115887_A_G	c.1985T>C	p.Ile662Thr	dVUS	≥3 of 5 in silico tools	1	-

chr3_169116121_T_A	c.1751A>T	p.Asp584Val	dVUS	≥3 of 5 in silico tools	2	-
chr3_169116241_G_T	c.1631C>A	p.Ala544Glu	dVUS	≥3 of 5 in silico tools	1	-
chr3_169116394_C_A	c.1478G>T	p.Gly493Val	dVUS	≥3 of 5 in silico tools	4	-
chr3_169116439_C_A	c.1433G>T	p.Gly478Val	dVUS	≥3 of 5 in silico tools	1	-
chr3_169116490_T_G	c.1382A>C	p.His461Pro	dVUS	≥3 of 5 in silico tools	1	-
chr3_169116646_T_C	c.1226A>G	p.Asp409Gly	dVUS	≥3 of 5 in silico tools	1	-
chr3_169116662_G_T	c.1210C>A	p.Gln404Lys	dVUS	≥3 of 5 in silico tools	1	-
chr3_169116685_C_T	c.1187G>A	p.Arg396His	dVUS	≥3 of 5 in silico tools	1	-
chr3_169121109_G_A	c.1079C>T	p.Ser360Leu	dVUS	≥3 of 5 in silico tools	1	-
chr3_169121116_C_G	c.1072G>C	p.Ala358Pro	dVUS	≥3 of 5 in silico tools	1	-
chr3_169121151_C_T	c.1037G>A	p.Arg346Gln	dVUS	≥3 of 5 in silico tools	4	-
chr3_169121152_G_A	c.1036C>T	p.Arg346Trp	dVUS	≥3 of 5 in silico tools	2	-
chr3_169121162_A_T	c.1026T>A	p.His342Gln	dVUS	≥3 of 5 in silico tools	1	-
chr3_169121172_C_T	c.1016G>A	p.Arg339His	dVUS	≥3 of 5 in silico tools	1	Y
chr3_169121181_C_T	c.1007G>A	p.Arg336Gln	dVUS	≥3 of 5 in silico tools	7	Y
chr3_169121202_G_A	c.986C>T	p.Thr329Met	dVUS	≥3 of 5 in silico tools	3	Y
chr3_169122599_T_C	c.959A>G	p.Glu320Gly	dVUS	≥3 of 5 in silico tools	1	-
chr3_169122603_A_G	c.955T>C	p.Tyr319His	dVUS	≥3 of 5 in silico tools	1	Y
chr3_169122636_G_A	c.922C>T	p.Arg308Cys	dVUS	≥3 of 5 in silico tools	2	Y
chr3_169127840_T_C	c.830+4A>G	-	dVUS	≥3 of 5 in silico tools	1	-
chr3_169127868_T_A	c.806A>T	p.Asp269Val	dVUS	≥3 of 5 in silico tools	1	Y
chr3_169127925_A_G	c.749T>C	p.Leu250Pro	dVUS	≥3 of 5 in silico tools	1	-
chr3_169128039_T_G	c.635A>C	p.Glu212Ala	dVUS	≥3 of 5 in silico tools	1	Y
chr3_169128040_C_T	c.634G>A	p.Glu212Lys	dVUS	≥3 of 5 in silico tools	32	Y
chr3_169128055_G_C	c.619C>G	p.Arg207Gly	dVUS	≥3 of 5 in silico tools	2	-
chr3_169131468_C_T	c.574G>A	p.Glu192Lys	dVUS	≥3 of 5 in silico tools	6	-
chr3_169381297_C_T	c.265G>A	p.Gly89Arg	dVUS	≥3 of 5 in silico tools	3	Y
chr3_169381344_T_C	c.218A>G	p.Asp73Gly	dVUS	≥3 of 5 in silico tools	1	-
chr3_169381374_G_C	c.188C>G	p.Pro63Arg	dVUS	≥3 of 5 in silico tools	37	Y
chr3_169381392_G_A	c.170C>T	p.Thr57Ile	dVUS	≥3 of 5 in silico tools	1	-

RUNX1 dVUS

Unique ID	HGVS c.	HGVS p.	Pathogenicity	Reason for Pathogenicity Call	DiscovEHR (# of heterozygotes)	Found in gnomAD (Y/-)
chr21_34792256_A_G	c.1322T>C	p.Leu441Pro	dVUS	≥3 of 5 in silico tools	2	-
chr21_34792283_C_T	c.1295G>A	p.Cys432Tyr	dVUS	≥3 of 5 in silico tools	1	Y
chr21_34792284_A_G	c.1294T>C	p.Cys432Arg	dVUS	≥3 of 5 in silico tools	1	-
chr21_34792310_C_G	c.1268G>C	p.Arg423Pro	dVUS	≥3 of 5 in silico tools	1	Y
chr21_34792325_A_C	c.1253T>G	p.Met418Arg	dVUS	≥3 of 5 in silico tools	4	Y
chr21_34792334_T_A	c.1244A>T	p.Gln415Leu	dVUS	≥3 of 5 in silico tools	1	-
chr21_34792337_T_C	c.1241A>G	p.Tyr414Cys	dVUS	≥3 of 5 in silico tools	1	-
chr21_34792364_A_G	c.1214T>C	p.Leu405Pro	dVUS	≥3 of 5 in silico tools	1	-
chr21_34792424_T_C	c.1154A>G	p.Tyr385Cys	dVUS	≥3 of 5 in silico tools	2	-
chr21_34792442_G_T	c.1136C>A	p.Thr379Asn	dVUS	≥3 of 5 in silico tools	1	-
chr21_34792443_T_C	c.1135A>G	p.Thr379Ala	dVUS	≥3 of 5 in silico tools	2	-
chr21_34792466_A_G	c.1112T>C	p.Met371Thr	dVUS	≥3 of 5 in silico tools	1	Y
chr21_34792474_C_A	c.1104G>T	p.Met368Ile	dVUS	≥3 of 5 in silico tools	1	-
chr21_34792485_C_G	c.1093G>C	p.Gly365Arg	dVUS	≥3 of 5 in silico tools	16	Y
chr21_34792485_C_T	c.1093G>A	p.Gly365Ser	dVUS	≥3 of 5 in silico tools	15	-
chr21_34792537_C_T	c.1041G>A	p.Met347Ile	dVUS	≥3 of 5 in silico tools	9	Y
chr21_34792544_G_C	c.1034C>G	p.Pro345Arg	dVUS	≥3 of 5 in silico tools	3	Y
chr21_34792568_G_T	c.1010C>A	p.Pro337His	dVUS	≥3 of 5 in silico tools	1	Y
chr21_34792571_A_G	c.1007T>C	p.Phe336Ser	dVUS	≥3 of 5 in silico tools	1	-
chr21_34792572_A_T	c.1006T>A	p.Phe336Ile	dVUS	≥3 of 5 in silico tools	3	-
chr21_34792574_T_C	c.1004A>G	p.Gln335Arg	dVUS	≥3 of 5 in silico tools	8	-
chr21_34792584_C_G	c.994G>C	p.Asp332His	dVUS	≥3 of 5 in silico tools	1	-
chr21_34792598_A_C	c.980T>G	p.Leu327Arg	dVUS	≥3 of 5 in silico tools	17	-
chr21_34799309_C_T	c.959G>A	p.Arg320Gln	dVUS	≥3 of 5 in silico tools	5	Y
chr21_34799351_C_T	c.917G>A	p.Arg306His	dVUS	≥3 of 5 in silico tools	1	Y
chr21_34799352_G_A	c.916C>T	p.Arg306Cys	dVUS	≥3 of 5 in silico tools	15	Y
chr21_34799369_G_A	c.899C>T	p.Thr300Met	dVUS	≥3 of 5 in silico tools	4	Y
chr21_34799435_G_A	c.833C>T	p.Pro278Leu	dVUS	≥3 of 5 in silico tools	1	-
chr21_34799462_T_C	c.806A>G	p.Asp269Gly	dVUS	≥3 of 5 in silico tools	2	-
chr21_34834425_G_C	c.790C>G	p.Gln264Glu	dVUS	≥3 of 5 in silico tools	17	-
chr21_34834428_G_A	c.787C>T	p.Pro263Ser	dVUS	≥3 of 5 in silico tools	9	Y
chr21_34834435_G_T	c.780C>A	p.Asn260Lys	dVUS	≥3 of 5 in silico tools	3	-
chr21_34834449_A_G	c.766T>C	p.Ser256Pro	dVUS	≥3 of 5 in silico tools	1	-
chr21_34834466_C_T	c.749G>A	p.Arg250His	dVUS	≥3 of 5 in silico tools	16	Y
chr21_34834467_G_A	c.748C>T	p.Arg250Cys	dVUS	≥3 of 5 in silico tools	23	Y
chr21_34834478_G_A	c.737C>T	p.Thr246Met	dVUS	≥3 of 5 in silico tools	17	Y

chr21_34834482_G_A	c.733C>T	p.Pro245Ser	dVUS	≥3 of 5 in silico tools	4	Y
chr21_34834487_G_A	c.728C>T	p.Pro243Leu	dVUS	≥3 of 5 in silico tools	3	-
chr21_34834503_C_T	c.712G>A	p.Val238Ile	dVUS	≥3 of 5 in silico tools	1	Y
chr21_34834518_G_A	c.697C>T	p.Arg233Cys	dVUS	≥3 of 5 in silico tools	1	Y
chr21_34834520_C_G	c.695G>C	p.Arg232Pro	dVUS	≥3 of 5 in silico tools	1	-
chr21_34834523_A_G	c.692T>C	p.Leu231Pro	dVUS	≥3 of 5 in silico tools	1	Y
chr21_34834524_G_T	c.691C>A	p.Leu231Met	dVUS	≥3 of 5 in silico tools	1	-
chr21_34834548_C_T	c.667G>A	p.Glu223Lys	dVUS	≥3 of 5 in silico tools	10	Y
chr21_34834554_A_T	c.661T>A	p.Phe221Ile	dVUS	≥3 of 5 in silico tools	1	-
chr21_34834557_A_C	c.658T>G	p.Ser220Ala	dVUS	≥3 of 5 in silico tools	2	-
chr21_34834558_C_G	c.657G>C	p.Leu219Phe	dVUS	≥3 of 5 in silico tools	4	Y
chr21_34834565_C_G	c.650G>C	p.Gly217Ala	dVUS	≥3 of 5 in silico tools	5	-
chr21_34834565_C_T	c.650G>A	p.Gly217Glu	dVUS	≥3 of 5 in silico tools	1	-
chr21_34834566_C_T	c.649G>A	p.Gly217Arg	dVUS	≥3 of 5 in silico tools	4	Y
chr21_34834570_C_A	c.645G>T	p.Lys215Asn	dVUS	≥3 of 5 in silico tools	1	-
chr21_34834574_G_A	c.641C>T	p.Thr214Ile	dVUS	≥3 of 5 in silico tools	24	Y
chr21_34834592_T_A	c.623A>T	p.Gln208Leu	dVUS	≥3 of 5 in silico tools	4	Y
chr21_34834595_C_T	c.620G>A	p.Arg207Gln	dVUS	≥3 of 5 in silico tools	4	-
chr21_34859495_C_T	c.592G>A	p.Asp198Asn	dVUS	≥3 of 5 in silico tools	1	-
chr21_34859510_T_C	c.577A>G	p.Ile193Val	dVUS	≥3 of 5 in silico tools	1	-
chr21_34859512_G_T	c.575C>A	p.Ala192Asp	dVUS	≥3 of 5 in silico tools	1	-
chr21_34859528_C_T	c.559G>A	p.Ala187Thr	dVUS	≥3 of 5 in silico tools	1	-
chr21_34859534_G_C	c.553C>G	p.Gln185Glu	dVUS	≥3 of 5 in silico tools	1	-
chr21_34859536_G_A	c.551C>T	p.Pro184Leu	dVUS	≥3 of 5 in silico tools	1	-
chr21_34859537_G_A	c.550C>T	p.Pro184Ser	dVUS	≥3 of 5 in silico tools	1	-
chr21_34859564_G_C	c.523C>G	p.Leu175Val	dVUS	≥3 of 5 in silico tools	1	Y
chr21_34880596_T_C	c.469A>G	p.Arg157Gly	dVUS	≥3 of 5 in silico tools	2	-
chr21_34880643_G_A	c.422C>T	p.Ser141Leu	dVUS	≥3 of 5 in silico tools	1	-
chr21_34880644_A_C	c.421T>G	p.Ser141Ala	dVUS	≥3 of 5 in silico tools	9	Y
chr21_34880651_T_A	c.414A>T	p.Glu138Asp	dVUS	≥3 of 5 in silico tools	10	Y
chr21_34880656_C_T	c.409G>A	p.Asp137Asn	dVUS	≥3 of 5 in silico tools	1	-
chr21_34880668_T_C	c.397A>G	p.Met133Val	dVUS	≥3 of 5 in silico tools	1	-
chr21_34880674_T_C	c.391A>G	p.Thr131Ala	dVUS	≥3 of 5 in silico tools	2	-
chr21_34880689_C_G	c.376G>C	p.Asp126His	dVUS	≥3 of 5 in silico tools	1	-
chr21_34880698_C_G	c.367G>C	p.Asp123His	dVUS	≥3 of 5 in silico tools	10	Y
chr21_34886857_G_A	c.337C>T	p.Pro113Ser	dVUS	≥3 of 5 in silico tools	1	-
chr21_34886862_G_A	c.332C>T	p.Thr111Ile	dVUS	≥3 of 5 in silico tools	1	-
chr21_34886862_G_T	c.332C>A	p.Thr111Asn	dVUS	≥3 of 5 in silico tools	2	-

chr21_34886869_T_C	c.325A>G	p.Asn109Asp	dVUS	≥3 of 5 in silico tools	1	-
chr21_34886883_G_A	c.311C>T	p.Thr104Met	dVUS	≥3 of 5 in silico tools	1	-
chr21_34886916_T_A	c.278A>T	p.Asp93Val	dVUS	≥3 of 5 in silico tools	1	-
chr21_34886920_T_C	c.274A>G	p.Thr92Ala	dVUS	≥3 of 5 in silico tools	1	-
chr21_34886923_G_A	c.271C>T	p.Arg91Cys	dVUS	≥3 of 5 in silico tools	1	-
chr21_34886935_C_A	c.259G>T	p.Gly87Cys	dVUS	≥3 of 5 in silico tools	19	Y
chr21_34886938_G_C	c.256C>G	p.Pro86Ala	dVUS	≥3 of 5 in silico tools	2	Y
chr21_34886955_T_G	c.239A>C	p.Glu80Ala	dVUS	≥3 of 5 in silico tools	4	Y
chr21_34886962_T_G	c.232A>C	p.Met78Leu	dVUS	≥3 of 5 in silico tools	4	-
chr21_34886983_G_C	c.211C>G	p.Leu71Val	dVUS	≥3 of 5 in silico tools	1	-
chr21_34886989_C_T	c.205G>A	p.Gly69Ser	dVUS	≥3 of 5 in silico tools	2	Y
chr21_34887001_C_G	c.193G>C	p.Ala65Pro	dVUS	≥3 of 5 in silico tools	1	-
chr21_34887024_G_C	c.170C>G	p.Pro57Arg	dVUS	≥3 of 5 in silico tools	1	-
chr21_34887039_A_T	c.155T>A	p.Met52Lys	dVUS	≥3 of 5 in silico tools	226	Y
chr21_34887048_G_A	c.146C>T	p.Pro49Leu	dVUS	≥3 of 5 in silico tools	4	Y
chr21_34887049_G_T	c.145C>A	p.Pro49Thr	dVUS	≥3 of 5 in silico tools	3	Y
chr21_34887067_G_A	c.127C>T	p.Pro43Ser	dVUS	≥3 of 5 in silico tools	1	-
chr21_34887072_G_T	c.122C>A	p.Thr41Lys	dVUS	≥3 of 5 in silico tools	1	-
chr21_34887079_G_T	c.115C>A	p.Arg39Ser	dVUS	≥3 of 5 in silico tools	1	-
chr21_34887103_G_A	c.98-7C>T	-	dVUS	≥3 of 5 in silico tools	1	-
chr21_34887109_G_A	c.98-13C>T	-	dVUS	≥3 of 5 in silico tools	1	-
chr21_35048844_C_T	c.56G>A	p.Arg19Lys	dVUS	≥3 of 5 in silico tools	8	Y
chr21_35048847_A_C	c.53T>G	p.Met18Arg	dVUS	≥3 of 5 in silico tools	1	-
chr21_35048865_G_A	c.35C>T	p.Ser12Leu	dVUS	≥3 of 5 in silico tools	5	Y
chr21_35048887_T_C	c.13A>G	p.Ser5Gly	dVUS	≥3 of 5 in silico tools	1	-
chr21_35048889_T_C	c.11A>G	p.Asp4Gly	dVUS	≥3 of 5 in silico tools	1	Y
chr21_35048959_C_T	c.-59-1G>A	-	dVUS	≥3 of 5 in silico tools	46	Y

***SRP72* dVUS**

Unique ID	HGVS c.	HGVS p.	Pathogenicity	Reason for Pathogenicity Call	DiscovEHR (# of heterozygotes)	Found in gnomAD (Y/-)
chr4_56467653_C_CG	c.25dup	p.Val9GlyfsTer9	dVUS	≥3 of 5 in silico tools	8	-
chr4_56467657_G_C	c.22G>C	p.Gly8Arg	dVUS	≥3 of 5 in silico tools	15	Y
chr4_56467657_G_T	c.22G>T	p.Gly8Trp	dVUS	≥3 of 5 in silico tools	18	-
chr4_56467658_G_T	c.23G>T	p.Gly8Val	dVUS	≥3 of 5 in silico tools	2	Y
chr4_56467664_C_T	c.29C>T	p.Ser10Leu	dVUS	≥3 of 5 in silico tools	4	Y
chr4_56467685_A_G	c.50A>G	p.Glu17Gly	dVUS	≥3 of 5 in silico tools	5	Y
chr4_56467711_G_T	c.76G>T	p.Asp26Tyr	dVUS	≥3 of 5 in silico tools	1	-
chr4_56467720_C_T	c.85C>T	p.Arg29Cys	dVUS	≥3 of 5 in silico tools	1	-
chr4_56467721_G_C	c.86G>C	p.Arg29Pro	dVUS	≥3 of 5 in silico tools	1	-
chr4_56467724_C_G	c.89C>G	p.Ala30Gly	dVUS	≥3 of 5 in silico tools	1	-
chr4_56469686_T_C	c.143T>C	p.Leu48Pro	dVUS	≥3 of 5 in silico tools	1	-
chr4_56469686_T_G	c.143T>G	p.Leu48Arg	dVUS	≥3 of 5 in silico tools	1	-
chr4_56469694_A_G	c.151A>G	p.Lys51Glu	dVUS	≥3 of 5 in silico tools	1	Y
chr4_56469706_C_G	c.163C>G	p.Leu55Val	dVUS	≥3 of 5 in silico tools	3	-
chr4_56469734_C_T	c.191C>T	p.Ala64Val	dVUS	≥3 of 5 in silico tools	45	Y
chr4_56471746_A_G	c.257A>G	p.Tyr86Cys	dVUS	≥3 of 5 in silico tools	1	-
chr4_56471751_G_A	c.262G>A	p.Glu88Lys	dVUS	≥3 of 5 in silico tools	1	-
chr4_56471841_G_A	c.352G>A	p.Val118Met	dVUS	≥3 of 5 in silico tools	2	-
chr4_56474053_G_T	c.355-1G>T	-	dVUS	≥3 of 5 in silico tools	12	-
chr4_56474060_C_T	c.361C>T	p.Arg121Cys	dVUS	≥3 of 5 in silico tools	1	-
chr4_56474061_G_A	c.362G>A	p.Arg121His	dVUS	≥3 of 5 in silico tools	4	-
chr4_56474072_T_C	c.373T>C	p.Tyr125His	dVUS	≥3 of 5 in silico tools	2	-
chr4_56474103_T_A	c.404T>A	p.Leu135His	dVUS	≥3 of 5 in silico tools	1	-
chr4_56474103_T_G	c.404T>G	p.Leu135Arg	dVUS	≥3 of 5 in silico tools	1	-
chr4_56474109_G_C	c.410G>C	p.Arg137Pro	dVUS	≥3 of 5 in silico tools	1	-
chr4_56474109_G_T	c.410G>T	p.Arg137Leu	dVUS	≥3 of 5 in silico tools	1	-
chr4_56474123_G_A	c.424G>A	p.Asp142Asn	dVUS	≥3 of 5 in silico tools	4	-
chr4_56474127_A_G	c.428A>G	p.Tyr143Cys	dVUS	≥3 of 5 in silico tools	1	-
chr4_56474132_G_A	c.433G>A	p.Glu145Lys	dVUS	≥3 of 5 in silico tools	2	-
chr4_56474133_A_G	c.434A>G	p.Glu145Gly	dVUS	≥3 of 5 in silico tools	2	Y
chr4_56474305_C_G	c.524C>G	p.Thr175Arg	dVUS	≥3 of 5 in silico tools	1	Y
chr4_56474319_T_C	c.538T>C	p.Tyr180His	dVUS	≥3 of 5 in silico tools	1	-
chr4_56474322_A_G	c.541A>G	p.Asn181Asp	dVUS	≥3 of 5 in silico tools	2	Y
chr4_56474344_G_C	c.563G>C	p.Gly188Ala	dVUS	≥3 of 5 in silico tools	1	Y
chr4_56474350_G_T	c.569G>T	p.Gly190Val	dVUS	≥3 of 5 in silico tools	2	Y
chr4_56476676_T_C	c.616T>C	p.Cys206Arg	dVUS	≥3 of 5 in silico tools	1	-
chr4_56476680_G_A	c.620G>A	p.Arg207His	dVUS	≥3 of 5 in silico tools	1	Y
chr4_56476698_A_C	c.638A>C	p.Asp213Ala	dVUS	≥3 of 5 in silico tools	1	-
chr4_56478412_G_A	c.676G>A	p.Ala226Thr	dVUS	≥3 of 5 in silico tools	1	-

chr4_56478421_C_G	c.685C>G	p.His229Asp	dVUS	≥3 of 5 in silico tools	1	-
chr4_56478425_G_A	c.689G>A	p.Gly230Asp	dVUS	≥3 of 5 in silico tools	1	-
chr4_56478432_G_A	c.696G>A	p.Met232Ile	dVUS	≥3 of 5 in silico tools	3	-
chr4_56478446_A_G	c.710A>G	p.Gln237Arg	dVUS	≥3 of 5 in silico tools	2	-
chr4_56478454_G_C	c.718G>C	p.Gly240Arg	dVUS	≥3 of 5 in silico tools	1	-
chr4_56478457_C_G	c.721C>G	p.Arg241Gly	dVUS	≥3 of 5 in silico tools	1	Y
chr4_56478485_A_G	c.749A>G	p.Asn250Ser	dVUS	≥3 of 5 in silico tools	1	Y
chr4_56478494_T_G	c.758T>G	p.Ile253Arg	dVUS	≥3 of 5 in silico tools	1	-
chr4_56478497_A_G	c.761A>G	p.Lys254Arg	dVUS	≥3 of 5 in silico tools	1	-
chr4_56478594_C_G	c.770C>G	p.Pro257Arg	dVUS	≥3 of 5 in silico tools	12	Y
chr4_56483138_G_GA	c.826-1_826insA	p.Asp276ArgfsTer6	dVUS	≥3 of 5 in silico tools	5	-
chr4_56483139_G_A	c.826G>A	p.Asp276Asn	dVUS	≥3 of 5 in silico tools	5	-
chr4_56483169_G_A	c.856G>A	p.Val286Met	dVUS	≥3 of 5 in silico tools	2	-
chr4_56483179_C_G	c.866C>G	p.Thr289Ser	dVUS	≥3 of 5 in silico tools	3	-
chr4_56483250_C_T	c.937C>T	p.Leu313Phe	dVUS	≥3 of 5 in silico tools	2	-
chr4_56483254_C_T	c.941C>T	p.Ala314Val	dVUS	≥3 of 5 in silico tools	5	Y
chr4_56483262_A_G	c.949A>G	p.Thr317Ala	dVUS	≥3 of 5 in silico tools	4	Y
chr4_56484743_A_G	c.965A>G	p.Gln322Arg	dVUS	≥3 of 5 in silico tools	1	-
chr4_56484748_C_T	c.970C>T	p.Arg324Cys	dVUS	≥3 of 5 in silico tools	7	Y
chr4_56484776_A_G	c.998A>G	p.Gln333Arg	dVUS	≥3 of 5 in silico tools	1	-
chr4_56484797_C_T	c.1019C>T	p.Pro340Leu	dVUS	≥3 of 5 in silico tools	3	-
chr4_56484806_T_C	c.1028T>C	p.Ile343Thr	dVUS	≥3 of 5 in silico tools	1	-
chr4_56484808_C_A	c.1030C>A	p.Gln344Lys	dVUS	≥3 of 5 in silico tools	1	-
chr4_56484826_C_G	c.1048C>G	p.Arg350Gly	dVUS	≥3 of 5 in silico tools	2	Y
chr4_56484826_C_T	c.1048C>T	p.Arg350Cys	dVUS	≥3 of 5 in silico tools	5	Y
chr4_56484864_G_C	c.1086G>C	p.Gln362His	dVUS	≥3 of 5 in silico tools	2	-
chr4_56486324_G_A	c.1087-1G>A	-	dVUS	≥3 of 5 in silico tools	8	-
chr4_56486353_C_G	c.1115C>G	p.Ala372Gly	dVUS	≥3 of 5 in silico tools	18	-
chr4_56486353_C_T	c.1115C>T	p.Ala372Val	dVUS	≥3 of 5 in silico tools	1	-
chr4_56486373_A_G	c.1135A>G	p.Met379Val	dVUS	≥3 of 5 in silico tools	72	Y
chr4_56489386_A_G	c.1225-2A>G	-	dVUS	≥3 of 5 in silico tools	3	-
chr4_56489407_T_C	c.1244T>C	p.Met415Thr	dVUS	≥3 of 5 in silico tools	1	-
chr4_56489408_G_A	c.1245G>A	p.Met415Ile	dVUS	≥3 of 5 in silico tools	1	-
chr4_56489445_G_T	c.1282G>T	p.Val428Phe	dVUS	≥3 of 5 in silico tools	1	-
chr4_56490382_A_T	c.1370A>T	p.Lys457Ile	dVUS	≥3 of 5 in silico tools	5	-
chr4_56490391_A_G	c.1379A>G	p.Tyr460Cys	dVUS	≥3 of 5 in silico tools	2	-
chr4_56490396_C_T	c.1384C>T	p.Arg462Trp	dVUS	≥3 of 5 in silico tools	12	-
chr4_56490583_T_G	c.1440T>G	p.Asp480Glu	dVUS	≥3 of 5 in silico tools	1	-
chr4_56490591_C_A	c.1448C>A	p.Thr483Asn	dVUS	≥3 of 5 in silico tools	9	Y
chr4_56490603_T_A	c.1460T>A	p.Leu487His	dVUS	≥3 of 5 in silico tools	1	Y
chr4_56490603_T_C	c.1460T>C	p.Leu487Pro	dVUS	≥3 of 5 in silico tools	6	Y

chr4_56490618_C_T	c.1475C>T	p.Ser492Leu	dVUS	≥3 of 5 in silico tools	5	-
chr4_56490627_A_G	c.1484A>G	p.Asp495Gly	dVUS	≥3 of 5 in silico tools	22	Y
chr4_56490646_G_A	c.1502+1G>A	-	dVUS	≥3 of 5 in silico tools	17	Y
chr4_56491435_A_C	c.1507A>C	p.Ser503Arg	dVUS	≥3 of 5 in silico tools	1	-
chr4_56491442_A_C	c.1514A>C	p.His505Pro	dVUS	≥3 of 5 in silico tools	1	-
chr4_56491447_C_T	c.1519C>T	p.Pro507Ser	dVUS	≥3 of 5 in silico tools	1	-
chr4_56491457_A_G	c.1529A>G	p.Asp510Gly	dVUS	≥3 of 5 in silico tools	1	Y
chr4_56491464_G_A	c.1536G>A	p.Met512Ile	dVUS	≥3 of 5 in silico tools	1	-
chr4_56491477_G_C	c.1549G>C	p.Asp517His	dVUS	≥3 of 5 in silico tools	1	-
chr4_56491481_T_C	c.1553T>C	p.Val518Ala	dVUS	≥3 of 5 in silico tools	1	-
chr4_56491484_A_G	c.1556A>G	p.Glu519Gly	dVUS	≥3 of 5 in silico tools	1	-
chr4_56491484_A_T	c.1556A>T	p.Glu519Val	dVUS	≥3 of 5 in silico tools	4	Y
chr4_56491519_C_T	c.1591C>T	p.Arg531Trp	dVUS	≥3 of 5 in silico tools	4	Y
chr4_56491544_G_C	c.1616G>C	p.Gly539Ala	dVUS	≥3 of 5 in silico tools	3	-
chr4_56491558_A_G	c.1630A>G	p.Lys544Glu	dVUS	≥3 of 5 in silico tools	1	-
chr4_56491559_A_T	c.1631A>T	p.Lys544Met	dVUS	≥3 of 5 in silico tools	1	-
chr4_56495395_G_A	c.1678+1G>A	-	dVUS	≥3 of 5 in silico tools	1	-
chr4_56500543_G_C	c.1686G>C	p.Leu562Phe	dVUS	≥3 of 5 in silico tools	19	-
chr4_56500596_T_C	c.1739T>C	p.Met580Thr	dVUS	≥3 of 5 in silico tools	1	Y
chr4_56500601_G_A	c.1744G>A	p.Glu582Lys	dVUS	≥3 of 5 in silico tools	1	-
chr4_56500610_T_C	c.1753T>C	p.Tyr585His	dVUS	≥3 of 5 in silico tools	1	Y
chr4_56500614_A_T	c.1757A>T	p.Tyr586Phe	dVUS	≥3 of 5 in silico tools	1	-
chr4_56500617_G_A	c.1760G>A	p.Arg587Gln	dVUS	≥3 of 5 in silico tools	7	Y
chr4_56500620_G_T	c.1763G>T	p.Gly588Val	dVUS	≥3 of 5 in silico tools	3	-
chr4_56500653_G_C	c.1796G>C	p.Gly599Ala	dVUS	≥3 of 5 in silico tools	1	-
chr4_56501688_G_A	c.1843G>A	p.Ala615Thr	dVUS	≥3 of 5 in silico tools	1	-
chr4_56501716_C_T	c.1871C>T	p.Thr624Ile	dVUS	≥3 of 5 in silico tools	1	Y
chr4_56501784_C_A	c.1939C>A	p.Gln647Lys	dVUS	≥3 of 5 in silico tools	1	-
chr4_56501833_A_T	c.1988A>T	p.Lys663Met	dVUS	≥3 of 5 in silico tools	1	-
