# Supplementary material for: Genome-first determination of the prevalence and penetrance of eight germline myeloid malignancy predisposition genes: a study of two population-based cohorts
Source: Leukemia. 2024 Nov 6;39(2):400–11. doi: 10.1038/s41375-024-02436-y (PMC11794151; doi:10.1038/s41375-024-02436-y)
Supplement: Supplementary file 2 — Supplementary File B [file 41375_2024_2436_MOESM2_ESM.pdf]

Supplemental Data

Supplemental Table S1: separate spreadsheet, File A

File B

Supplemental Table S2

ICD-9-CM Category	Chapter II (Neoplasms) ICD-9-CM Term	ICD-10-CM Category	Chapter II (Neoplasms) ICD-10-CM Term
**HEMATOLOGICAL MALIGNANCIES**			
200.0	Reticulosarcoma	C85.1	Unspecified B-cell lymphoma
200.1	Lymphosarcoma	C83.5	Lymphoblastic (diffuse) lymphoma
		C83.0	Small cell B-cell lymphoma
200.2	Burkitt's tumor of lymphoma	C83.7	Burkitt lymphoma
200.3	Marginal Zone Lymphoma	C88.4	Extra nodal marginal zone B-cell lymphoma of mucosa- associated lymphoid tissue [MALT lymphoma]
		C83.0	Small cell B-cell lymphoma
200.4	Mantle Cell Lymphoma	C83.1	Mantle cell lymphoma
200.5	Primary central nervous system lymphoma	C83.3	Diffuse large B-cell lymphoma
		C83.9	Non-follicular (diffuse) lymphoma, unspecified
200.6	Anaplastic large cell lymphoma	C83.3	Diffuse large B-cell lymphoma
		C84.6	Anaplastic large cell lymphoma, ALK-positive
		C84.7	Anaplastic large cell lymphoma, ALK-negative
		C86.6	Primary cutaneous CD30-positive T-cell lymphoma
200.7	Large cell lymphoma	C83.3	Diffuse large B-cell lymphoma
		C83.8	Other types of non-Hodgkin's lymphoma
		C85.2	Mediastinal (thymic) large B-cell lymphoma
		C83.9	Non-follicular (diffuse) lymphoma, unspecified
200.8	Other named variants of lymphosarcoma and Reticulosarcoma	C83.8	Other types of non-Hodgkin's lymphoma
201.0	Hodgkin's paragranuloma	C81.0	Nodular lymphocyte predominant Hodgkin lymphoma
201.1	Hodgkin's granuloma	C81.7	Other classical Hodgkin lymphoma
201.2	Hodgkin's sarcoma	C81.7	Other classical Hodgkin lymphoma
201.4	Hodgkin's disease, lymphocytic-histiocytic predominance	C81.0	Nodular lymphocyte predominant Hodgkin lymphoma
		C81.4	Lymphocyte-rich Hodgkin lymphoma
201.5	Hodgkin's disease, nodular sclerosis	C81.1	Nodular sclerosis classical Hodgkin lymphoma
201.6	Hodgkin's disease, mixed cellularity	C81.2	Mixed cellularity classical Hodgkin lymphoma
201.7	Hodgkin's disease, lymphocytic depletion	C81.3	Lymphocyte depleted classical Hodgkin lymphoma

201.9	Hodgkin's disease, unspecified type	C81.9	Hodgkin lymphoma, unspecified
202.0	Nodular lymphomas	C82.9	Follicular lymphoma, unspecified
		C82.0	Follicular lymphoma grade I
		C82.1	Follicular lymphoma grade II
		C82.2	Follicular lymphoma grade III, unspecified
		C82.3	Follicular lymphoma grade IIIa
		C82.4	Follicular lymphoma grade IIIb
		C82.5	Diffuse follicle center lymphoma
		C82.6	Cutaneous follicle center lymphoma
		C82.8	Other types of follicular lymphoma
202.1	Mycosis fungoides	C84.0	Mycosis fungoides
202.2	Sezary's disease	C84.1	Sezary's disease
202.3	Malignant histiocytosis	C96.A	Histiocytic sarcoma
202.4	Leukemic reticuloendotheliosis	C91.4	Hairy cell leukemia
202.5	Letterer-Siwe disease	C96.0	Multifocal and multisystemic (disseminated) Langerhans cell histiocytosis
		C96.6	Unifocal Langerhans-cell histiocytosis
202.6	Malignant mast-cell tumors	C96.22	Mast cell sarcoma
		C96.20	Malignant mast cell neoplasm, unspecified
		C96.21	Aggressive systemic mastocytosis
202.7	Peripheral T-cell lymphoma	C84.4	Peripheral T-cell lymphoma, not elsewhere classified
		C86.5	Angioimmunoblastic T-cell lymphoma
202.8	Other malignant lymphomas	C85.9	Non-Hodgkin lymphoma, unspecified
		C84.A	Cutaneous T-cell lymphoma, unspecified
		C84.Z	Other mature T/NK-cell lymphomas
		C84.9	Mature T/NK-cell lymphomas, unspecified
		C85.1	Unspecified B-cell lymphoma
		C85.8	Other specified types of non-Hodgkin lymphoma
		C86.0	Extranodal NK/T-cell lymphoma, nasal type
		C86.1	Hepatosplenic T-cell lymphoma
		C86.2	Enteropathy-type (intestinal) T-cell lymphoma
		C86.3	Subcutaneous panniculitis-like T-cell lymphoma
		C86.4	Blastic NK-cell lymphoma
202.9	Other and unspecified malignant neoplasm of lymphoid and histiocytic tissue	C96.Z	Other specified malignant neoplasms of lymphoid, hematopoietic, and related tissue
		C96.4	Sarcoma of dendritic cells (accessory cells)
203.0	Multiple myeloma	C90.0	Multiple myeloma
203.1	Plasma cell leukemia	C90.1	Plasma cell leukemia
203.8	Other immunoproliferative neoplasms	C90.2	Extramedullary plasmacytoma
		C90.3	Solitary plasmacytoma not having achieved remission
		C88.2	Heavy chain disease

		C88.3	Immunoproliferative small intestinal disease
		C88.9	Malignant immunoproliferative disease, unspecified
204.0	Acute lymphoid leukemia	C91.0	Acute lymphoblastic leukemia
204.1	Chronic lymphoid leukemia	C91.1	Chronic lymphocytic leukemia
204.2	Subacute lymphoid leukemia	C91.9	Lymphoid leukemia, unspecified
204.8	Other lymphoid leukemia	C91.Z	Other lymphoid leukemia
		C91.A	Mature B-cell leukemia Burkitt type
		C91.3	Prolymphocytic leukemia of B-cell type
		C91.6	Prolymphocytic leukemia of T-cell type
204.9	Unspecified lymphoid leukemia	C91.9	Lymphoid leukemia
205.0	Acute myeloid leukemia	C92.0	Acute myeloblastic leukemia
		C92.4	Acute promyelocytic leukemia
		C92.6	Acute myeloid leukemia with 11q23 abnormality
		C92.A	Acute myeloid leukemia with multilineage dysplasia
		C94.4	Acute panmyelosis with myelofibrosis
		C94.8	Other specified leukemias
205.1	Chronic myeloid leukemia	D47.1	Chronic myeloproliferative disease
		C92.1	Chronic myeloid leukemia, BCR/ABL-positive
		C92.2	Atypical chronic myeloid leukemia, BCR/ABL-negative
		C95.1	Chronic leukemia of unspecified cell type
205.2	Subacute myeloid leukemia	C92.9	Myeloid leukemia, unspecified
205.3	Myeloid sarcoma	C92.3	Myeloid sarcoma
205.8	Other myeloid leukemia	C92.Z	Other myeloid leukemia
205.9	Unspecified myeloid leukemia	C92.9	Myeloid leukemia
206.0	Acute monocytic leukemia	C93.0	Acute monoblastic/monocytic leukemia
		C92.5	Acute myelomonocytic leukemia
206.1	Chronic monocytic leukemia	C93.1	Chronic myelomonocytic leukemia
		C93.3	Juvenile myelomonocytic leukemia
206.2	Subacute monocytic leukemia	C93.9	Monocytic leukemia, unspecified
206.8	Other monocytic leukemia	C93.9	Monocytic leukemia, unspecified
206.9	Unspecified monocytic leukemia	C93.9	Monocytic leukemia, unspecified
207.0	Acute erythremia and erythroleukemia	C94.0	Acute erythroid leukemia
207.1	Chronic erythremia	D45	Polycythemia vera
207.2	Megakaryocytic leukemia	C94.2	Acute megakaryoblastic leukemia
207.8	Other specified leukemia	C94.8	Other specified leukemias
		C94.3	Mast cell leukemia
208.0	Acute leukemia	C95.0	Acute leukemia of unspecified cell type
208.1	Chronic leukemia	C95.1	Chronic leukemia of unspecified cell type
208.2	Subacute leukemia	C95.9	Leukemia, unspecified
208.8	Other leukemia of unspecified cell type	C95.9	Leukemia, unspecified
208.9	Unspecified leukemia	C95.9	Leukemia, unspecified
238.71	Essential thrombocythemia	D47.3	Essential (hemorrhagic) thrombocythemia
238.72	Low grade myelodysplastic syndrome lesions	D46.4	Refractory anemia, unspecified
		D46.0	Refractory anemia without ring sideroblasts, so stated
		D46.1	Refractory anemia with ring sideroblasts

		D46.20	Refractory anemia with excess of blasts, unspecified
		D46.21	Refractory anemia with excess of blasts 1
		D46.22	Refractory anemia with excess of blasts 2
		D46.A	Refractory cytopenia with multilineage dysplasia
		D46.B	Refractory cytopenia with multilineage dysplasia and ring sideroblasts
238.73	High grade myelodysplastic syndrome lesions	D46.20	Refractory anemia with excess of blasts, unspecified
		D46.21	Refractory anemia with excess of blasts 1
		D46.22	Refractory anemia with excess of blasts 2
238.74	Myelodysplastic syndrome with 5q deletion	D46.C	Myelodysplastic syndrome with isolated del (5q) chromosomal abnormality
238.75	Myelodysplastic syndrome, unspecified	D46.9	Myelodysplastic syndrome, unspecified
		D46.Z	Other myelodysplastic syndromes
238.76	Myelofibrosis with myeloid metaplasia	D47.4	Osteomyelofibrosis
238.79	Other lymphatic and hematopoietic tissues	D47.Z9	Other specified neoplasms of uncertain behavior of lymphoid, hematopoietic and related tissue
		D47.9	Neoplasm of uncertain behavior of lymphoid, hematopoietic and related tissue, unspecified
		C86.6	Primary cutaneous CD30-positive T-cell proliferations
273.3	Macroglobulinemia	C88.0	Waldenstrom’s macroglobulinemia
V42.81	Bone marrow replaced by transplant	Z94.81	Bone marrow transplant status
V42.82	Peripheral stem cells replaced by transplant	Z94.84	Stem cell transplant status
**MYELOID MALIGNANCIES (SUBSET)**			
205.0	Acute myeloid leukemia	C92.0	Acute myeloblastic leukemia
		C92.4	Acute promyelocytic leukemia
		C92.6	Acute myeloid leukemia with 11q23 abnormality
		C92.A	Acute myeloid leukemia with multilineage dysplasia
		C94.4	Acute panmyelosis with myelofibrosis
		C94.8	Other specified leukemias
205.1	Chronic myeloid leukemia	D47.1	Chronic myeloproliferative disease
		C92.1	Chronic myeloid leukemia, BCR/ABL-positive
		C92.2	Atypical chronic myeloid leukemia, BCR/ABL-negative
		C95.1	Chronic leukemia of unspecified cell type
205.2	Subacute myeloid leukemia	C92.9	Myeloid leukemia, unspecified
205.3	Myeloid sarcoma	C92.3	Myeloid sarcoma
205.8	Other myeloid leukemia	C92.Z	Other myeloid leukemia
205.9	Unspecified myeloid leukemia	C92.9	Myeloid leukemia
206.0	Acute monocytic leukemia	C93.0	Acute monoblastic/monocytic leukemia
		C92.5	Acute myelomonocytic leukemia
206.1	Chronic monocytic leukemia	C93.1	Chronic myelomonocytic leukemia
		C93.3	Juvenile myelomonocytic leukemia

206.2	Subacute monocytic leukemia	C93.9	Monocytic leukemia, unspecified
206.8	Other monocytic leukemia	C93.9	Monocytic leukemia, unspecified
206.9	Unspecified monocytic leukemia	C93.9	Monocytic leukemia, unspecified
207.0	Acute erythremia and erythroleukemia	C94.0	Acute erythroid leukemia
207.2	Megakaryocytic leukemia	C94.2	Acute megakaryoblastic leukemia
207.8	Other specified leukemia	C94.8	Other specified leukemias
		C94.3	Mast cell leukemia
238.72	Low grade myelodysplastic syndrome lesions	D46.4	Refractory anemia, unspecified
		D46.0	Refractory anemia without ring sideroblasts, so stated
		D46.1	Refractory anemia with ring sideroblasts
		D46.20	Refractory anemia with excess of blasts, unspecified
		D46.21	Refractory anemia with excess of blasts 1
		D46.22	Refractory anemia with excess of blasts 2
		D46.A	Refractory cytopenia with multilineage dysplasia
		D46.B	Refractory cytopenia with multilineage dysplasia and ring sideroblasts
238.73	High grade myelodysplastic syndrome lesions	D46.20	Refractory anemia with excess of blasts, unspecified
		D46.21	Refractory anemia with excess of blasts 1
		D46.22	Refractory anemia with excess of blasts 2
238.74	Myelodysplastic syndrome with 5q deletion	D46.C	Myelodysplastic syndrome with isolated del (5q) chromosomal abnormality
238.75	Myelodysplastic syndrome, unspecified	D46.9	Myelodysplastic syndrome, unspecified
		D46.Z	Other myelodysplastic syndromes

Supplemental Table S3

**A**	P/LP Variants				Heterozygotes	
	DiscovEHR	UKBB	In Common	Total	DiscovEHR	UKBB
CEBPA	3	6	1	8	4	22
DDX41	21	57	11	67	196	815
ETV6	4	12	1	15	4	17
GATA2	5	5	1	9	7	6
MECOM	14	33	6	41	41	85
RUNX1	9	8	2	15	11	8
SRP72	12	36	5	43	20	96
Total	68	157	27	198	283	1049

**B**	DiscovEHR	
	dVUS	Heterozygotes
ANKRD26	109	468
CEBPA	10	15
DDX41	77	261
ETV6	33	52
GATA2	114	655
MECOM	67	306
RUNX1	101	667
SRP72	109	472
Total	620	2896

Supplemental Table S4

			n	Ratio (95% CI)	p
**Total (all genes)**	DiscovEHR	Prevalence	283	1:602 (1:497-1:621)	0.0018
		Penetrance	11	1:26 (1:15-1:46)	0.039
	UKBB	Prevalence	1049	1:448 (1:421-1:476)	0.0022
		Penetrance	55	1:19 (1:15-1:25)	0.052
CEBPA	DiscovEHR	Prevalence	4	1:42,626 (1:15,550-1:133,063)	2.4×10^{-5}
		Penetrance	0	0	0
	UKBB	Prevalence	22	1:21,345 (1:14,097-32,321)	4.7×10^{-5}
		Penetrance	1	1:22 (1:4-1:421)	0.45
DDX41	DiscovEHR	Prevalence	196	1:870 (1:756-1:1000)	0.0012
		Penetrance	5	1:39 (1:17-1:91)	0.026
	UKBB	Prevalence	815	1:576 (1:538-617)	0.0017
		Penetrance	43	1:19 (1:14-1:25)	0.052
ETV6	DiscovEHR	Prevalence	4	1:42,626 (1:15,550-1:133,063)	2.4×10^{-5}
		Penetrance	1	1:4 (1:1.3-1:76)	0.25
	UKBB	Prevalence	17	1:27,623 (1:17,248-1:44,241)	3.6×10^{-5}
		Penetrance	3	1:6 (1:2-1:21)	0.17
GATA2	DiscovEHR	Prevalence	7	1:24,358 (1:11,799-1:50,283)	4.1×10^{-5}
		Penetrance	2	1:4 (1:1.4-1:20)	0.28
	UKBB	Prevalence	6	1:78,266 (1:35,870-170,771)	1.3×10^{-5}
		Penetrance	0	0	0
MECOM	DiscovEHR	Prevalence	41	1:4159 (1:3066-1:5641)	0.00024
		Penetrance	0	0	0
	UKBB	Prevalence	85	1:5525 (1:4469-1:6830)	0.00018
		Penetrance	1	1:85 (1:14-1:1628)	0.012
RUNX1	DiscovEHR	Prevalence	11	1:15,500 (1:8656-1:27,758)	6.4×10^{-5}
		Penetrance	3	1:4 (1:2-1:14)	0.27
	UKBB	Prevalence	8	1:58,699 (1:29,745-1:115,840)	1.7×10^{-5}
		Penetrance	5	1:2 (1:1.2-1:3)	0.63
SRP72	DiscovEHR	Prevalence	20	1:8525 (1:5519-1:13,169)	0.00012
		Penetrance	0	0	0
	UKBB	Prevalence	96	1:4892 (1:4008-1:5973)	0.00020
		Penetrance	2	1:48 (1:12-1:276)	0.042
Hem TFs	DiscovEHR	Prevalence	67	1:2545 (1:3344-1:6351)	0.00022
		Penetrance	6	1:11 (1:5-1:24)	0.090
	UKBB	Prevalence	138	1:3403 (1:2881-1:4022)	0.00029
		Penetrance	10	1:14 (1:8-1:25)	0.072
GATA2, *ETV6*, *RUNX1*	DiscovEHR	Prevalence	22	1:7750 (1:5119-1:11,735)	0.00013
		Penetrance	4	1:4 (1:2-1:8)	0.27
	UKBB	Prevalence	31	1:15,148 (1:10,673-1:21,501)	6.6×10^{-5}
		Penetrance	8	1:4 (1:2-1:7)	0.26

Supplemental Table S5

			n (heterozygotes)	Frequency	%
**Total (all genes)**	P/LP	DiscovEHR	283	0.000166	0.17
		UKBB	1049	0.00223	0.22
CEBPA	P/LP	DiscovEHR	4	$2.35x10^{-5}$	0.0024
		UKBB	22	$4.68x10^{-5}$	0.0047
DDX41	P/LP	DiscovEHR	196	0.00115	0.12
		UKBB	815	0.00174	0.17
ETV6	P/LP	DiscovEHR	4	$2.35x10^{-5}$	0.002
		UKBB	17	$3.62x10^{-5}$	0.0036
GATA2	P/LP	DiscovEHR	7	$4.11x10^{-5}$	0.0041
		UKBB	6	$1.28x10^{-5}$	0.0013
MECOM	P/LP	DiscovEHR	41	0.000240	0.024
		UKBB	85	0.000181	0.018
RUNX1	P/LP	DiscovEHR	11	$6.45x10^{-5}$	0.0064
		UKBB	8	$1.70x10^{-5}$	0.0017
SRP72	P/LP	DiscovEHR	20	0.000117	0.012
		UKBB	96	0.000204	0.020
Hem TFs	P/LP	DiscovEHR	67	0.000393	0.039
		UKBB	138	0.000294	0.029
GATA2, *ETV6*, *RUNX1*	P/LP	DiscovEHR	22	0.000129	0.013
		UKBB	31	$6.60x10^{-5}$	0.0066

Supplemental Table S6

A. DiscovEHR

CEBPA	*DDX41*	*ETV6*	*GATA2*	*MECOM*	*RUNX1*	*SRP72*
none	AML	MDS	AML	none	MDS	none
	MDS		MDS		AML	
	CLL		CMML		CMML	
	DLBCL				Waldenström macroglobulinemia	
	HL					
	Mantle Cell Lymphoma					
	AML					

B. UKBB

CEBPA	*DDX41*	*ETV6*	*GATA2*	*MECOM*	*RUNX1*	*SRP72*
AML	AML	AML	none	AML	AML	CLL
	MDS	MDS			MDS	HL
	NHL	CMML			CML	
	MM					
	DLBCL					
	ET					
	CLL					
	Malignant histiocytosis / dendritic cell sarcoma					

Supplemental Table S7

A	DiscovEHR					
	All Individuals		Non-Heterozygotes		Heterozygotes	
	Mean (sd)	Range	Mean (sd)	Range	Mean (sd)	Range
Current Age, mean (sd)	58.4 (19)	1.8-90+	58.4 (19)	1.8-90+	56.9 (19)	5.1-90+
Age at Sample Collection	52.5 (18)	0.01-90+	52.6 (18)	0.01-90+	51.1 (19)	2.3-90+
Age at Hematological Malignancy Diagnosis	62.9 (16)	0.01-90+	62.9 (16)	0.01-90+	64.5 (15)	34-90+
Age at Myeloid Malignancy Diagnosis	67.7 (15)	0.01-90+	67.7 (15)	0.01-90+	67.2 (14)	45-90+

B	UKBB					
	All Individuals		Non-Heterozygotes		Heterozygotes	
	Mean (sd)	Range	Mean (sd)	Range	Mean (sd)	Range
Current Age, mean (sd)	70.0 (8.1)	40-88	70.0 (8.1)	40-88	70.2 (8.1)	47-85
Age at Sample Collection	56.5 (8.1)	37-73	56.5 (8.1)	37-73	56.9 (8.2)	40-70
Age at Hematological Malignancy Diagnosis	60.4 (12)	4.7-82	60.4 (12)	4.7-82	66.6 (8.8)	41-79
Age at Myeloid Malignancy Diagnosis	62.12 (11)	12-81	62.0 (11)	12-81	68.3 (6.9)	48-79

Supplemental Table Legends

Supplemental Table S1: Spreadsheet (attached separately, File A), variants identified in all cohorts, two tabs per gene- one for pathogenic/likely pathogenic (P/LP) for DiscovEHR, UKBB and gnomAD; and one for variants of uncertain significance with evidence of deleteriousness (dVUS) in DiscovEHR. Variants level details provided.

Supplemental Table S2: ICD-9CM and -10CM codes used in phenotype analysis. Information obtained from https://seer.cancer.gov/tools/conversion/. Hematological malignancy (HM) category for analysis includes all codes listed here. Myeloid malignancy (MM) group is a subset of HM and contains those listed in the MM section.

Supplemental Table S3: A) Frequency of variants for pathogenic/likely pathogenic (P/LP) in DiscovEHR and UKBB and corresponding number of heterozygote individuals for all eight genes studied. B) Frequency of variants of uncertain significance with evidence of deleteriousness (dVUS) in DiscovEHR and corresponding heterozygote individuals.

Supplemental Table S4: Prevalence ratio, penetrance of hematological malignancy ratio, 95% confidence intervals (CI) and p values for myeloid malignancy predisposition genes (gMMP) in DiscovEHR and UKBB. Hem TFs: hematopoietic transcription factor genes- *CEBPA*, *GATA2*, *ETV6*, *MECOM*, *RUNX1*.

Supplemental Table S5: Frequencies and percentage of heterozygotes with a unique pathogenic/likely pathogenic (P/LP) for individual gMMP genes. DiscovEHR, n=170,503; UKBB, n=469,595. Hem TFs: hematopoietic transcription factor genes- *CEBPA*, *GATA2*, *ETV6*, *MECOM*, *RUNX1*.

Supplemental Table S6: Hematological malignancies identified in each cohort by gene. AML: acute myeloid leukemia, MDS: myelodysplastic syndrome, CLL chronic lymphocytic leukemia, DLBCL: diffuse large B-cell lymphoma, HL: Hodgkin lymphoma, CMML: chronic myelomonocytic leukemia, NHL: non-Hodgkin lymphoma, MM: multiple myeloma, ET: essential thrombocythemia (chronic myeloproliferative neoplasm), CML: chronic myeloid leukemia.

Supplemental Table S7: Current age, age at sample collection, and age at malignancy for all individuals, non-heterozygotes and heterozygotes in A) DiscovEHR and B) UKBB

Supplemental Figure S1

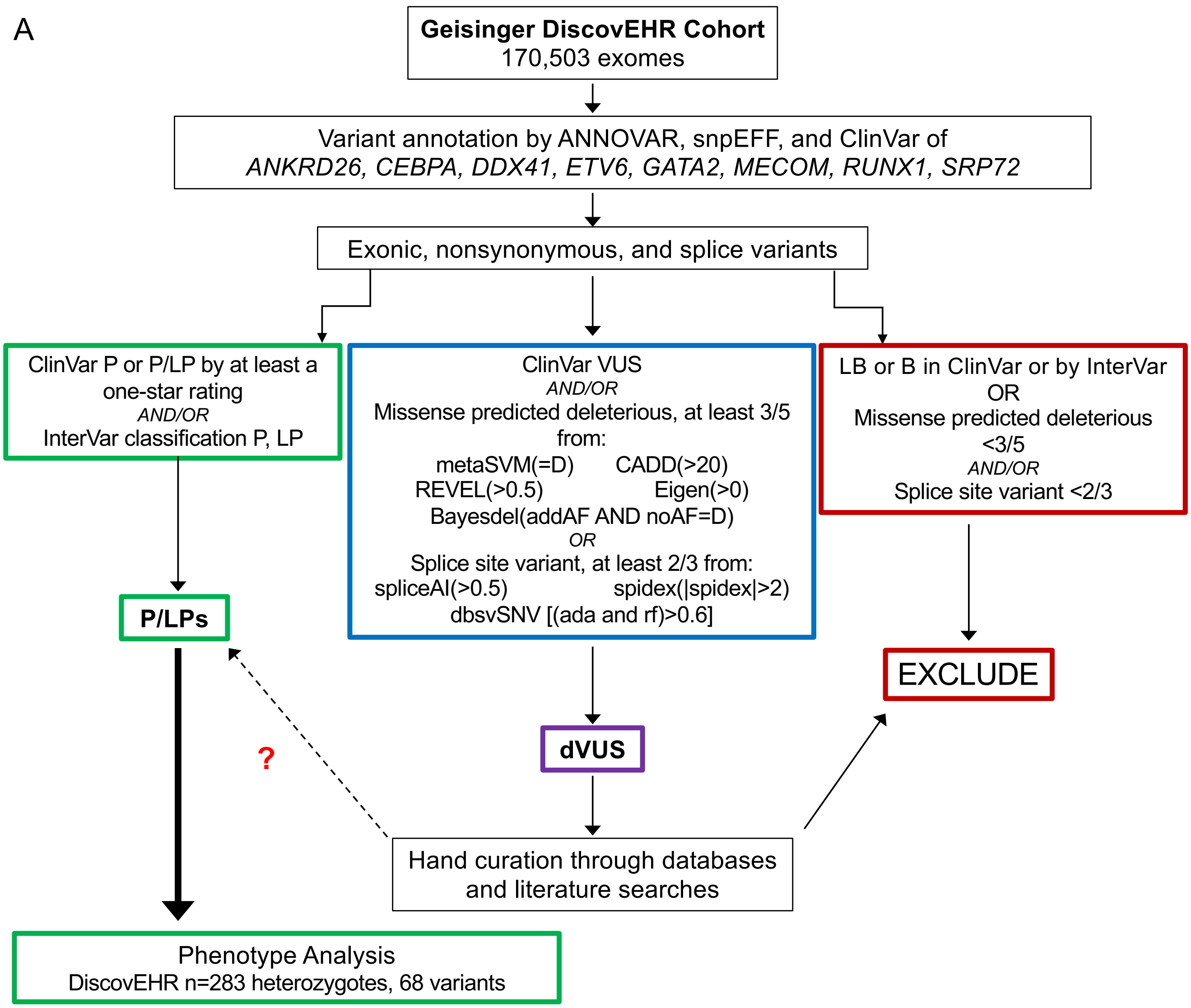

B

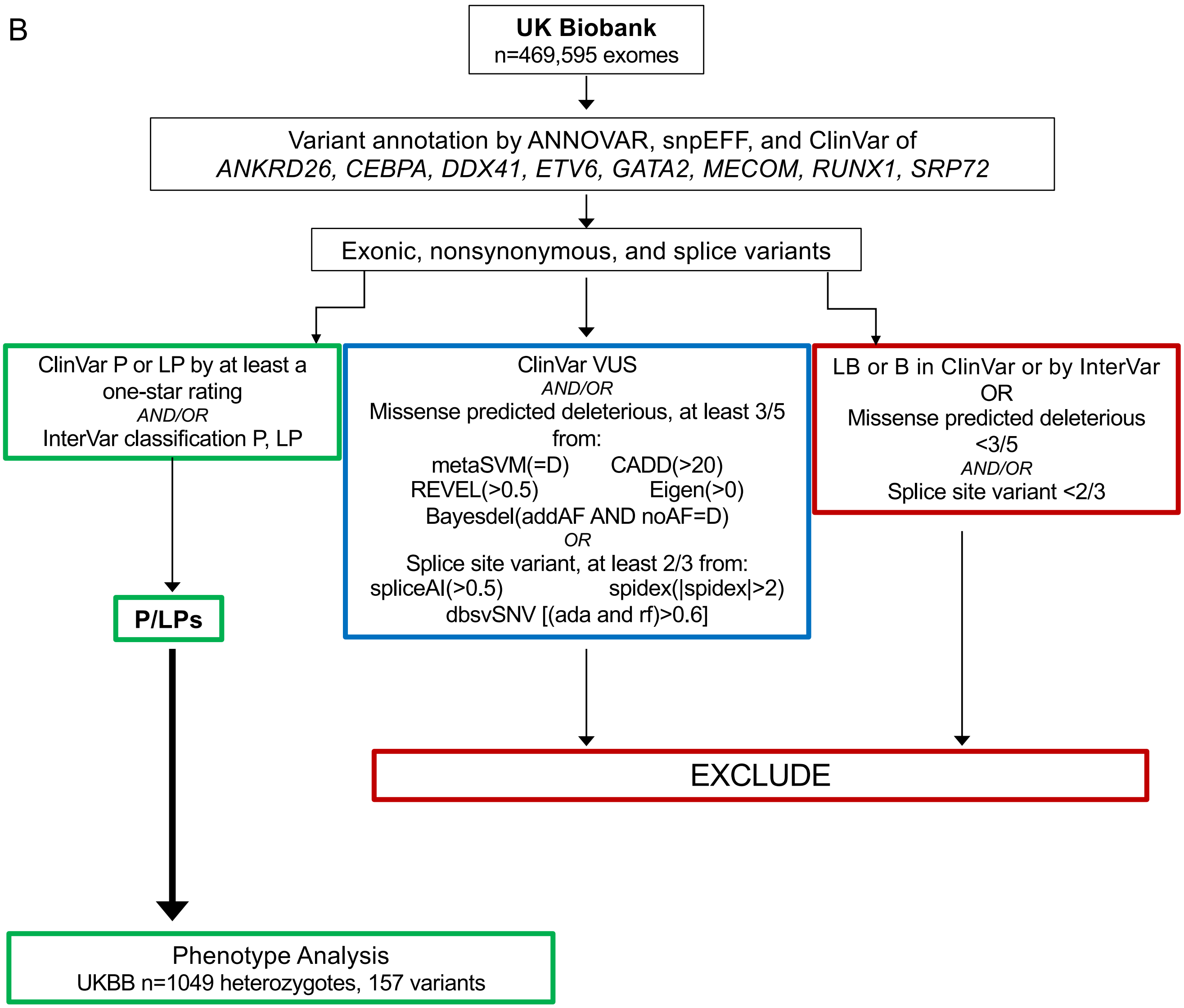
UK Biobank
n=469,595 exomes
Variant annotation by ANNOVAR, snpEFF, and ClinVar of
ANKRD26, CEBPA, DDX41, ETV6, GATA2, MECOM, RUNX1, SRP72
Exonic, nonsynonymous, and splice variants
ClinVar P or LP by at least a one-star rating
AND/OR
InterVar classification P, LP
ClinVar VUS
AND/OR
Missense predicted deleterious, at least 3/5 from:
metaSVM(=D)
CADD(>20)
REVEL(>0.5)
Eigen(>0)
Bayesdel(addAF AND noAF=D)
OR
Splice site variant, at least 2/3 from:
spliceAI(>0.5)
spidex(|spidex|>2)
dbsvSNV [(ada and rf)>0.6]
LB or B in ClinVar or by InterVar
OR
Missense predicted deleterious <3/5
AND/OR
Splice site variant <2/3
P/LPs
EXCLUDE
Phenotype Analysis
UKBB n=1049 heterozygotes, 157 variants

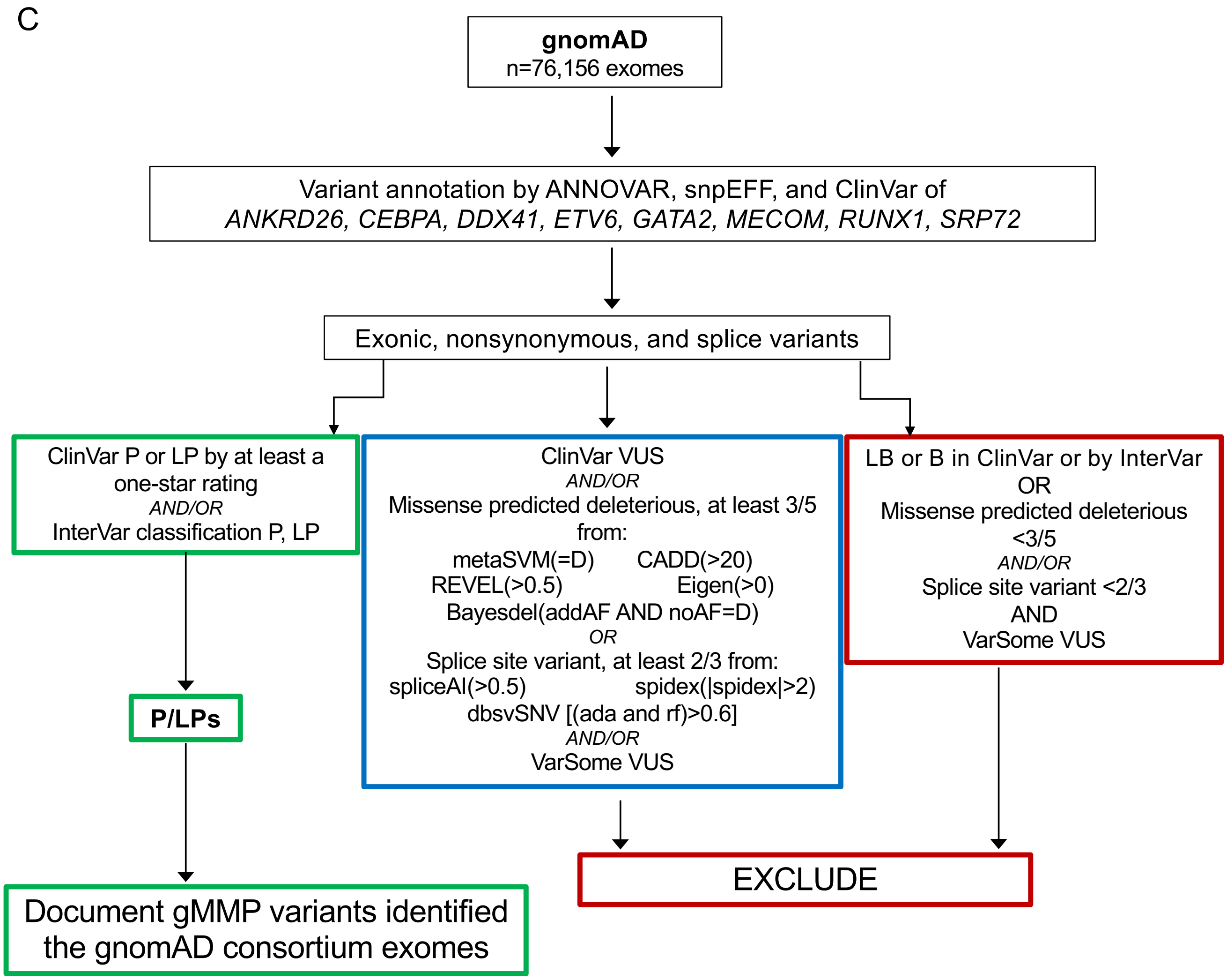
C
gnomAD
n=76,156 exomes
Variant annotation by ANNOVAR, snpEFF, and ClinVar of
ANKRD26, CEBPA, DDX41, ETV6, GATA2, MECOM, RUNX1, SRP72
Exonic, nonsynonymous, and splice variants
ClinVar P or LP by at least a one-star rating
AND/OR
InterVar classification P, LP
ClinVar VUS
AND/OR
Missense predicted deleterious, at least 3/5 from:
metaSVM(=D) CADD(>20)
REVEL(>0.5) Eigen(>0)
Bayesdel(addAF AND noAF=D)
OR
Splice site variant, at least 2/3 from:
spliceAI(>0.5) spidex(|spidex|>2)
dbsvSNV [(ada and rf)>0.6]
AND/OR
VarSome VUS
LB or B in ClinVar or by InterVar
OR
Missense predicted deleterious <3/5
AND/OR
Splice site variant <2/3
AND
VarSome VUS
P/LPs
EXCLUDE
Document gMMP variants identified the gnomAD consortium exomes

Supplemental Figure S2

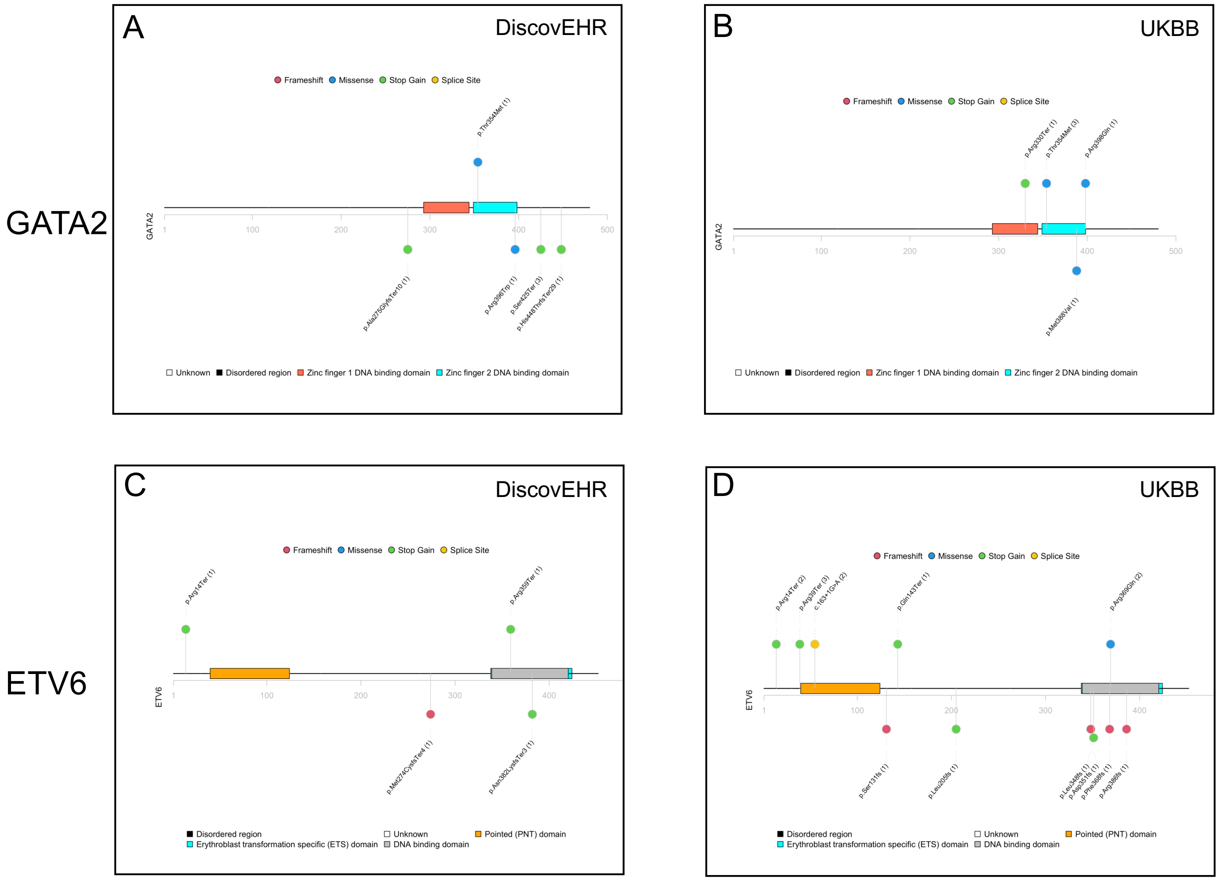

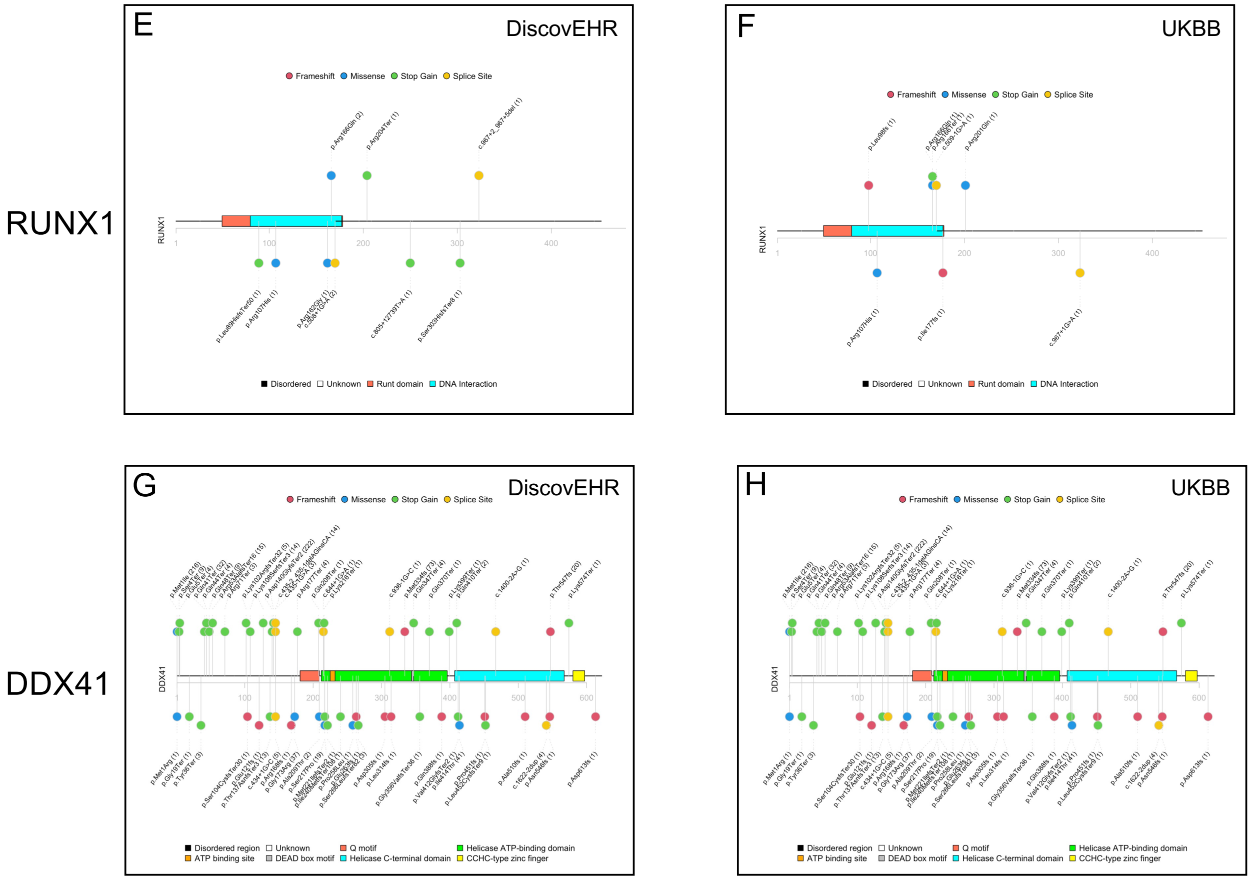
RUNX1
DDX41
E
DiscovEHR
F
UKBB
G
DiscovEHR
H
UKBB
Frameshift Missense Stop Gain Splice Site
Disordered Unknown Runt domain DNA Interaction
Disordered region Unknown Q motif Helicase ATP-binding domain
ATP binding site DEAD box motif Helicase C-terminal domain CCHC-type zinc finger

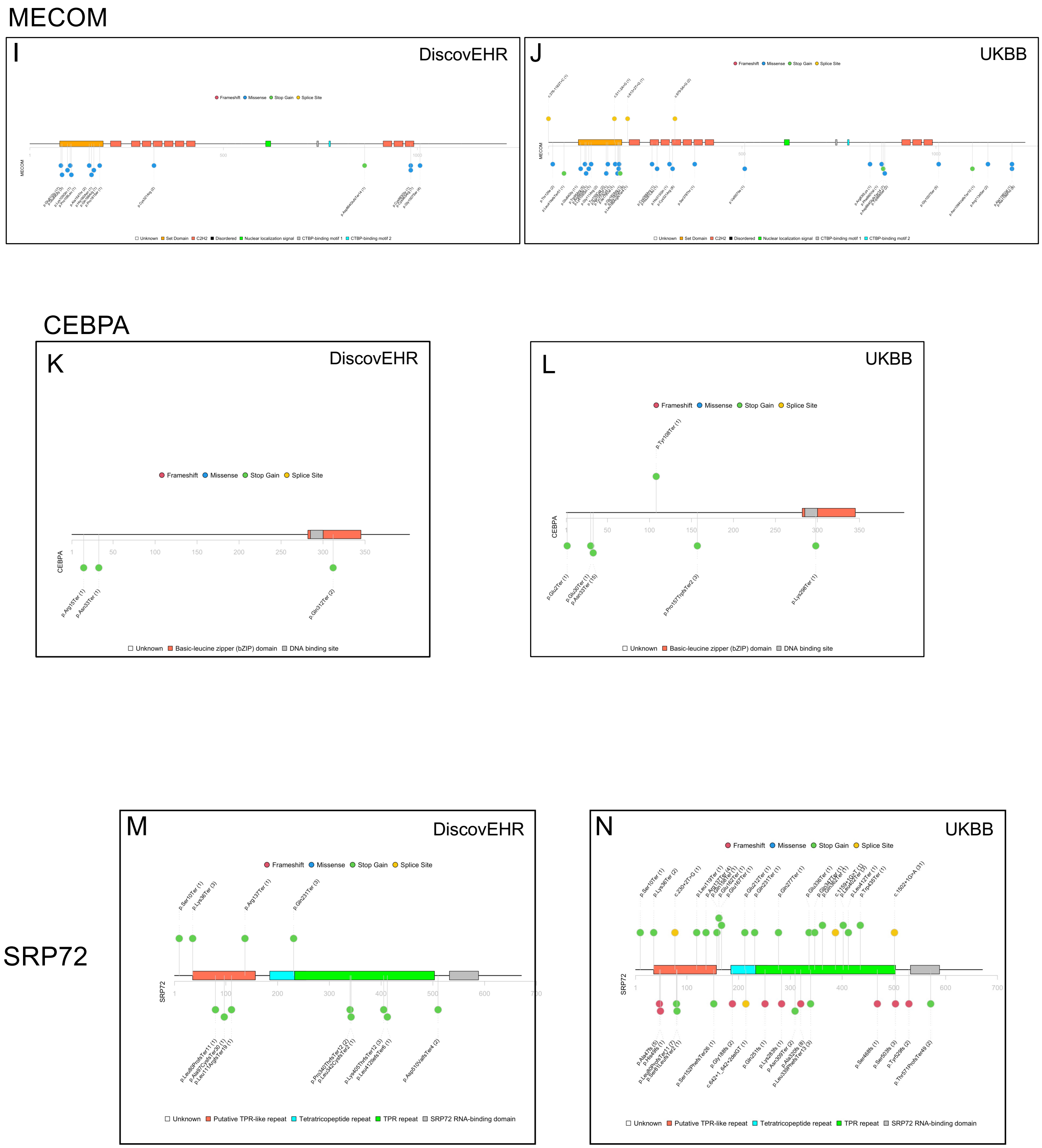
MECOM
I
DiscovEHR
Frameshift Missense Stop Gain Splice Site
J
UKBB
CEBPA
K
DiscovEHR
Frameshift Missense Stop Gain Splice Site
p.Arg15Ter (1)
p.Asn33Ter (1)
p.Gln312Ter (2)
Unknown Basic-leucine zipper (bZIP) domain DNA binding site
L
UKBB
p.Tyr108Ter (1)
p.Glu2Ter (1)
p.Pro157TrpfsTer2 (3)
p.Lys298Ter (1)
SRP72
M
DiscovEHR
p.Ser10Ter (1)
p.Lys36Ter (3)
p.Arg137Ter (1)
p.Gln231Ter (3)
p.Asn510ValfsTer4 (2)
Unknown Putative TPR-like repeat Tetratricopeptide repeat TPR repeat SRP72 RNA-binding domain
N
UKBB
p.Ser10Ter (1)
p.Lys36Ter (2)
p.Gln277Ter (1)
c.1502+1G>A (31)
p.Ser468fs (1)
p.Ser503fs (3)
p.Tyr529fs (2)
p.Thr557ProfsTer49 (2)
Unknown Putative TPR-like repeat Tetratricopeptide repeat TPR repeat SRP72 RNA-binding domain

Supplemental Figure S3

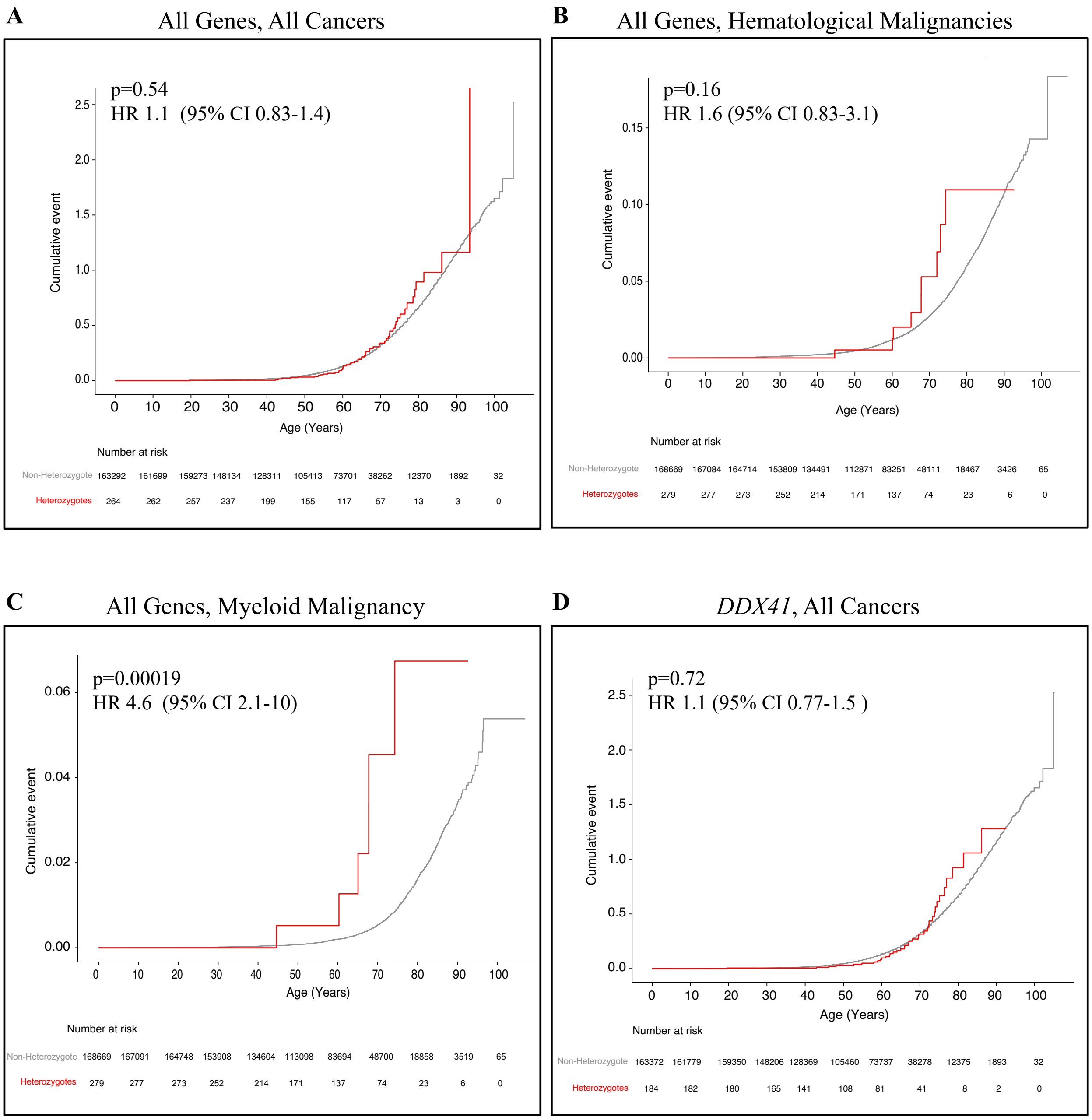

Supplemental Figure S4

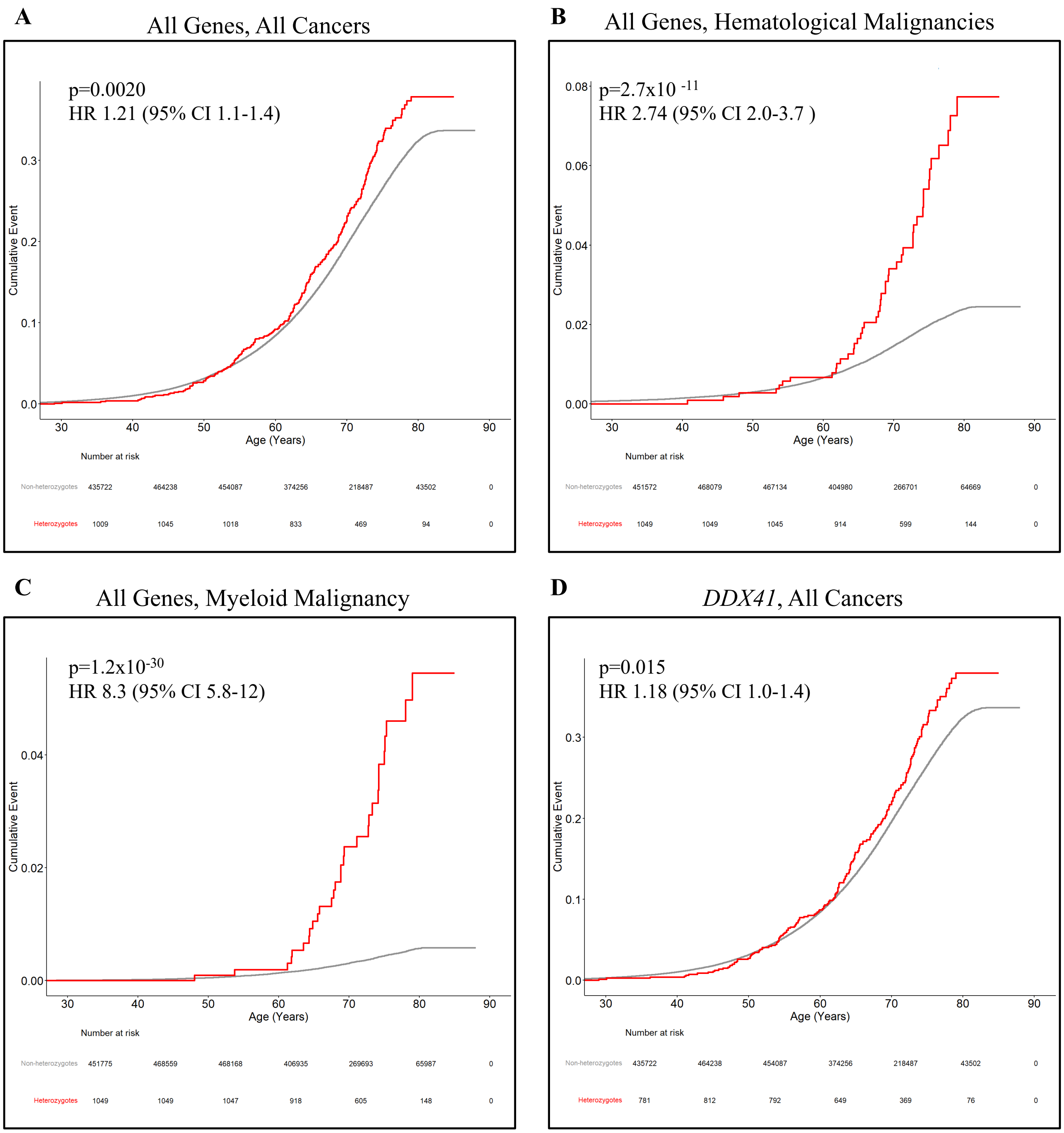

Supplemental Figure Legends

Supplemental Figure S1: Schematic of Methodology. (A) For the population-based cohort, DiscovEHR, variants were classified as shown in the schema. Individuals with pathogenic/likely pathogenic (P/LP) variants underwent cancer phenotype analysis. Variants of uncertain significance with evidence of deleteriousness (dVUS) were hand curated to determine if literature or database evidence could upgrade these variants to P/LP. No dVUS were re-classified as P/LP after hand curation. (B) UK Biobank exomes were evaluated for the presence of P/LP variants and cancer phenotype analyzed. (C) gnomAD P/LP variants were documented.

Supplemental Figure S2: Lolliplots of pathogenic/likely pathogenic variants identified in DiscovEHR (A, C, E, G, I, K, M) and UKBB (B, D, F, H, J, L, N).

Supplemental Figure S3: Time-dependent cumulative risk, time to cancer development for heterozygotes of gMMP genes (red) compared with non-heterozygotes (gray) in the DiscovEHR cohort. (A) all cancers, (B) hematological malignancies, (C) myeloid malignancies, (D) *DDX41*, all cancers (E) *RUNX1*, *ETV6* and *GATA2* all cancers. Log-rank p values compared the heterozygote curve to non-heterozygote curve.

Supplemental Figure S4: Time-dependent cumulative risk, time to cancer development for heterozygotes of gMMP genes (red) compared with non-heterozygotes (gray) in the UKBB cohort. (A) all cancers, (B) hematological malignancies, (C) myeloid malignancies, (D) *DDX41*, all cancers (E) *RUNX1*, *ETV6* and *GATA2* all cancers. Log-rank p values compared the heterozygote curve to non-heterozygote curve.
